# Pragmatic Clinical Trial Assessing Response to Neoadjuvant Docetaxel and Trastuzumab in Nigerian Women With Human Epidermal Growth Factor Receptor 2–Positive Breast Cancer (ARETTA)

**DOI:** 10.1200/GO-25-00287

**Published:** 2026-05-29

**Authors:** Atara Ntekim, Abiodun Popoola, Anthonia Sowunmi, Olalekan Olasehinde, Ayodele Sanni, Abiola Ibraheem, Yonglan Zheng, Toshio F. Yoshimatsu, Gideon T. Dosunmu, Olasubomi Omoleye, Ayorinde Folasire, Adenike Adeniji-Sofoluwe, Adeleye Omisore, Alabi Adewumi, Thomas Olajide, Razak Lawal, Akinwunmi Komolafe, Foluso Omodele, Mustapha Ajani, Obaro Michael, Babajide Balogun, Olagoke Korede Ale, Nicholas Irurhe, Sharif Folorunso, Ademola Oyekan, Olayinka Kotila, Toyin Aniagwu, Abayomi Odetunde, Siljander Ilona, Ajibola Olufadekemi Ajani, Adekunle Oshin, Adedayo Onitilo, Chioma Asuzu, Adetola Daramola, Fatimah Abdulkareem, Onyinye Offor Balogun, Theodore Karrison, Peace Babalola, Dezheng Huo, Christopher O. Olopade, John Obafunwa, Oladosu Ojengbede, Olufunmilayo I. Olopade

**Affiliations:** ^1^ Department of Radiation Oncology, College of Medicine, University of Ibadan, Ibadan, Nigeria; ^2^ Department of Radiation Oncology, Lagos State University College of Medicine, Lagos, Nigeria; ^3^ Department of Radiation Oncology, College of Medicine, University of Lagos, Lagos, Nigeria; ^4^ Department of Surgery, College of Medicine, Obafemi Awolowo University, Ile-Ife, Nigeria; ^5^ Department of Pathology, Lagos State University College of Medicine, Lagos, Nigeria; ^6^ Department of Medicine, University of Illinois College of Medicine, Chicago, IL; ^7^ Department of Medicine, The University of Chicago, Chicago, IL; ^8^ Department of Radiology, College of Medicine, University of Ibadan, Ibadan, Nigeria; ^9^ Department of Radiology, College of Medicine, Obafemi Awolowo University, Ile-Ife, Nigeria; ^10^ Department of Surgery, Lagos University Teaching Hospital, Lagos, Nigeria; ^11^ Department of Morbid Anatomy and Forensic Medicine, Obafemi Awolowo University (OAU), Ile-Ife, Nigeria; ^12^ Department of Surgery, College of Medicine, Lagos State University, Ikeja, Nigeria; ^13^ Department of Pathology, College of Medicine, University of Ibadan, Ibadan, Nigeria; ^14^ Department of Pharmacology and Therapeutics, University of Ibadan, Ibadan, Nigeria; ^15^ Department of Radiology, Lagos State University Teaching Hospital Ikeja, Lagos, Nigeria; ^16^ Department of Medicine, College of Medicine, University of Lagos, Lagos, Nigeria; ^17^ Department of Radiology, College of Medicine, University of Lagos, Lagos, Nigeria; ^18^ Department of Pharmaceutical Chemistry, University of Ibadan, Ibadan, Nigeria; ^19^ Department of Nursing Education, University College Hospital, Ibadan, Nigeria; ^20^ Genetic and Bioethics Research Unit IMRAT, College of Medicine University of Ibadan, Ibadan, Nigeria; ^21^ Healthy Life for All Foundation, Ibadan, Nigeria; ^22^ Marshfield Clinic, Marshfield, WI; ^23^ Department of Anatomic Pathology, University of Lagos, Lagos, Nigeria; ^24^ Department of Radiation Oncology, Weill Cornell College of Medicine, New York, NY; ^25^ Department of Public Health Sciences, The University of Chicago, Chicago, IL; ^26^ Centre for Population and Reproductive Health, College of Medicine, Ibadan, Nigeria

## Abstract

**PURPOSE:**

Breast cancer is the leading cause of death in Nigerian women. Clinical trials are required to provide evidence for treatment. We aimed to generate efficacy data to support the shift in local practice of neoadjuvant therapy for breast cancer.

**METHODS:**

A one-stage, phase II feasibility, single-arm study design was used. Treatment-naïve patients with clinically nonmetastatic human epidermal growth factor receptor 2 (HER2)–positive breast cancer received four cycles of neoadjuvant docetaxel with subcutaneous trastuzumab (T + scH). Patients with complete clinical response underwent surgery. Patients with stable disease/partial response received three further cycles of 5-fluorouracil, epirubicin, and cyclophosphamide + scH before surgery. Responders completed 18 scH cycles. The primary end point was pathologic complete response (pCR). The secondary end points were toxicity and invasive disease-free survival.

**RESULTS:**

A total of 53 female patients age 18-70 years were enrolled. The median age of the 47 evaluable female patients was 50 years. pCR was achieved in 25 of 47 patients (53% [95% CI, 38.1 to 67.9]; *P* < .001). Forty-two percent of patients were estrogen receptor+ and 40% were progesterone receptor+ (95% CI, 20.3 to 66.5 and 21.5 to 69.2, respectively). All were HER2+, with 40% stage II and 60% stage III disease. Three patients (6.4%) experienced grade 3 myelosuppression, one (2.1%) experienced grade 3 diarrhea, two (4.3%) had a grade 4 adverse event, and two developed hepatitis while on the study medications. The reporting of disease-free and overall survival awaits future analysis.

**CONCLUSION:**

This phase II study showed that neoadjuvant chemotherapy with T + scH resulted in pCR of 53%, surpassing the planned cutoff for success of 40%. This is comparable with the rates and other efficacy end points that follow data from phase II international trials. This regimen may be useful in the absence of pertuzumab and warrants further investigation.

## INTRODUCTION

Breast cancer is a heterogeneous disease and prototype of health disparities worldwide. An estimated three million women will be diagnosed with breast cancer by 2040, with the fastest growth rate among premenopausal women in low- and middle-income countries (LMICs).^1^ Although breast cancer mortality has decreased since the 1990s in high-income countries, mortality rates are beginning to rise in many LMICs as women live longer.^2^ The increasing mortality gap within and across countries within the same region is striking. Differences in tumor biology, genome architecture, and inequities in health care delivery patterns have been reported to contribute to the gap in breast cancer mortality.^3,4^ To meet the Sustainable Development Goals for 2030, the United Nations has emphasized the importance of innovation, science, and technology in improving health and wellness in all populations.^5^ The WHO recently released a new Global Breast Cancer Initiative Framework, providing a roadmap to attain the target of saving 2.5 million lives from breast cancer by 2040.^6^

**CONTEXT**

**Key Objective**
Despite significant improvements in the treatment of human epidermal growth factor receptor 2 (HER2)–positive breast cancer, no clinical trials have evaluated the efficacy of chemotherapy combined with HER2-targeted therapy for early-stage HER2-positive breast cancer in sub-Saharan Africa.
**Knowledge Generated**
Optimal treatment with HER2 blockades can result in a response rate among indigenous Black women with HER2 positive breast cancer, similar to other populations, thus improving the outcome of breast cancer treatment. This pragmatic clinical trial addressed the unmet clinical need to translate the findings from decades of genetic epidemiologic research on young-onset breast cancer into meaningful benefits for Nigerian women.
**Relevance**
The lessons learned can now be applied to design further studies to optimize the standard of care for HER2-positive breast cancer to reduce premature deaths from breast cancer in Nigeria. The need to improve facilities and make essential medicines available for breast cancer management is also highlighted.

The overrepresentation of aggressive breast cancer subtypes delineated by estrogen receptor (ER), progesterone receptor (PR), and human epidermal growth factor receptor 2 (HER2) status across the diaspora have been described.^7,8^ Although novel targeted treatments are becoming increasingly expensive, most women in low-resource settings have poor access to quality diagnostic tools and optimal cancer care. Gains in survival achieved through improvements in early detection and prompt multimodality treatment in Europe and North America have widened global disparities in outcomes between the poor and rich countries.^9-11^

HER2-positive breast cancer is currently the most curable molecular subtype owing to HER2-directed therapy, such as trastuzumab and neoadjuvant chemotherapy. Pathologic complete response (pCR), disease-free survival (DFS), and overall survival (OS) were significantly improved compared with chemotherapy alone.^12,13^ The use of neoadjuvant chemotherapy alone without HER2-directed therapy is still a common practice in Nigeria.^14^ A study reported that only approximately 14% of eligible Nigerian patients with HER2-positive breast cancer received trastuzumab.^15^ To address this gap, we conducted a multi-institutional, biomarker-informed, clinical trial. The primary objective of this study was to assess the effectiveness of adding subcutaneous (SC) trastuzumab (T + scH) to standard chemotherapy for early-stage HER2-positive breast cancer.

## METHODS

### Study Design

The ARETTA study was a one-stage, phase II, single-arm clinical trial in women with nonmetastatic HER2-positive breast cancer. All patients received four cycles of neoadjuvant docetaxel (75 mg/m^2^) + trastuzumab (600 mg) SC given every 3 weeks. Docetaxel dosing was started at 75 mg/m^2^ and increased to 100 mg/m^2^, if tolerated. Breast ultrasound (USS) was performed at diagnosis and after every two cycles of the neoadjuvant phase. Patients with a complete clinical response (CCR) to USS after four cycles underwent surgery. Those with partial response or stable disease (operable) received 5-fluorouracil (5-FU) (600 mg/m^2^), epirubicin (90 mg/m^2^), and cyclophosphamide (600 mg/m^2^) (FEC) every 3 weeks for an additional three cycles before surgery. All responders received 18 cycles of trastuzumab SC every 3 weeks. Adjuvant treatment included hormonal therapy as indicated. Radiation therapy was administered according to the institutional practice.

This study was conducted across four Academic Hospitals in Nigeria, and the protocol was approved by the institutional review boards (IRBs) of all study sites. The study was conducted in accordance with the principles of the Declaration of Helsinki and ICH-GCP. Written informed consent was obtained from all participants before their enrollment in the study. This study has been registered at ClinicalTrials.gov (identifier: NCT03879577).

### Participants

To be eligible, participants were required to be aged between 18 and 70 years, with histologically confirmed HER2-positive breast cancer (3+ by immunochemistry). The eligible stages included anatomic stages IIA–IIIC (American Joint Committee on Cancer 2009). Other inclusion criteria were biopsy-accessible tumors, tumors that were clinically considered nonmetastatic, chemotherapy-naïve, and an Eastern Cooperative Oncology Group (ECOG) performance status of 0-1. Essential baseline laboratory data required adequate hematologic, renal, and hepatic function. A baseline left ventricular ejection fraction of ≥55% was also necessary.

The exclusion criteria included pregnant or lactating women, women not using effective contraception, postmenopausal women not amenorrheic for at least 12 months, serious uncontrolled infections, participation in investigational drug studies within 4 weeks before the study, and other serious medical conditions that could compromise participation, such as uncontrolled hypertension, diabetes, psychiatric illnesses limiting compliance, and HIV infection. Patients living with HIV were excluded because HIV-positive patients may experience more interruptions in chemotherapy, often because of infections or low CD4 counts, which may lead to lower compliance. The patients provided written consent to participate in the study by signing an IRB-approved consent form.

### Procedures

A central review of the immunohistochemistry (IHC) slides was conducted within 48 hours by the pathology subcommittee of the clinical trial. HER2 status was scored and interpreted according to the ASCO 2018 guidelines as follows: 0 (negative), 1+ (negative), 2+ (equivocal/borderline), and 3+ (positive).

Only patients with a score of 3+ were included in the study. Patients with 2+ were excluded because there were no facilities for fluorescent in situ hybridization (FISH) or chromogenic in situ hybridization testing.

Echocardiography (ECHO) was performed at the time of screening and/or baseline, every 12 weeks during the neoadjuvant phase, and every 3 months while on trastuzumab alone. Thereafter, ECHO was performed every 6 months for the first 2 years after trastuzumab treatment. Treatment with trastuzumab was to be withheld for a minimum of 3 weeks (one cycle) if left ventricular ejection fraction (LVEF) was below 50% and or ≥15% points decreased from baseline. If LVEF returns to 50% or higher, treatment can be resumed. If the LVEF has not improved or has declined further after a repeat assessment within approximately 3-7 weeks (two cycles), trastuzumab should be discontinued.

Patients who achieved a CCR after four cycles underwent surgery within 4 weeks of the last dose. Partial responders or patients with stable disease received three additional cycles of FEC (5-FU, 600 mg/m^2^; epirubicin, 90 mg/m^2^; and cyclophosphamide, 600 mg/m^2^ + scH) every 3 weeks before surgery. Trastuzumab administration was not interrupted during surgery. A total of 18 trastuzumab doses were administered. Additionally, premenopausal patients received goserelin every 3 months for 2 years. Patients with disease progression or stable (inoperable) disease were excluded from the study.

The chemotherapy regimen involved tailored dose modifications based on the National Cancer Institute (NCI) Common Terminology Criteria for Adverse Events version 5.0. Adverse events, including cardiac issues, were recorded using the NCI Common Toxicity Criteria, version 5.0. Cardiac health was closely monitored using electrocardiography and ECHO at specified treatment milestones.

### Outcomes

The primary outcome measure of the study was the overall pCR. Patients who achieved pCR with the addition of FEC + scH were included in the primary end point analysis and were counted as successes. Those who were inoperable or had progressed were counted as nonresponders. All patients were followed up for long-term outcomes. The primary outcome measure was centrally assessed by a pathology working group. pCR was analyzed using the ypT0 ypN0 definition (invasive or noninvasive residual disease in the breast or nodes).

The secondary outcomes of this study included the safety profile and 10-year invasive DFS (iDFS). The iDFS end points will be reported in future analyses. An interim safety analysis was performed after the first 20 patients underwent surgery. The Data Safety Monitoring Board reviewed this interim report to ensure that there were no logistical or safety issues warranting protocol discontinuation, such as an unacceptable rate of noncompliance with the study protocol or adverse events.

### Statistical Analysis

The target sample size for this study was calculated based on the expected pCR. An inefficient treatment was hypothesized to elicit 20% or fewer positive responses (p0 = 0.20), while a successful treatment regimen was expected to elicit at least 40% positive responses (p1 = 0.40). These estimates were based on reported pCR rates of 46.8% and 56.3% for stage II and III Brazilian patients with breast cancer treated with neoadjuvant trastuzumab and chemotherapy, respectively.^16^ To achieve a power of 90% at a one-sided significance level of α = 0.05, 47 patients were required for evaluation. If 14 or fewer responses were observed, the treatment regimen was rejected. The planned cutoff for success was a pCR of 40%. Accounting for a 10% nonevaluable rate, this study aimed to enroll 53 patients.

The primary analysis was based on the evaluation of the pCR rates. The pCR rates in different subgroups were compared using the Fisher exact test. Adverse events were summarized descriptively. After long-term follow-up, Kaplan-Meier curves will be generated for iDFS and prognostic factors will be assessed by Cox regression modeling.^17^ Statistical analyses were performed using the Stata version 17 by StataCorp LLC, College Station, TX.

### Role of the Funding Source

The sponsors supplied the study drugs and provided financial support but imposed no restrictions on the investigators with respect to study design, data collection, data analysis, data interpretation, and writing of the report. The corresponding author had full access to all data and had the final responsibility for the decision to submit for publication.

## RESULTS

**FIG 1:**
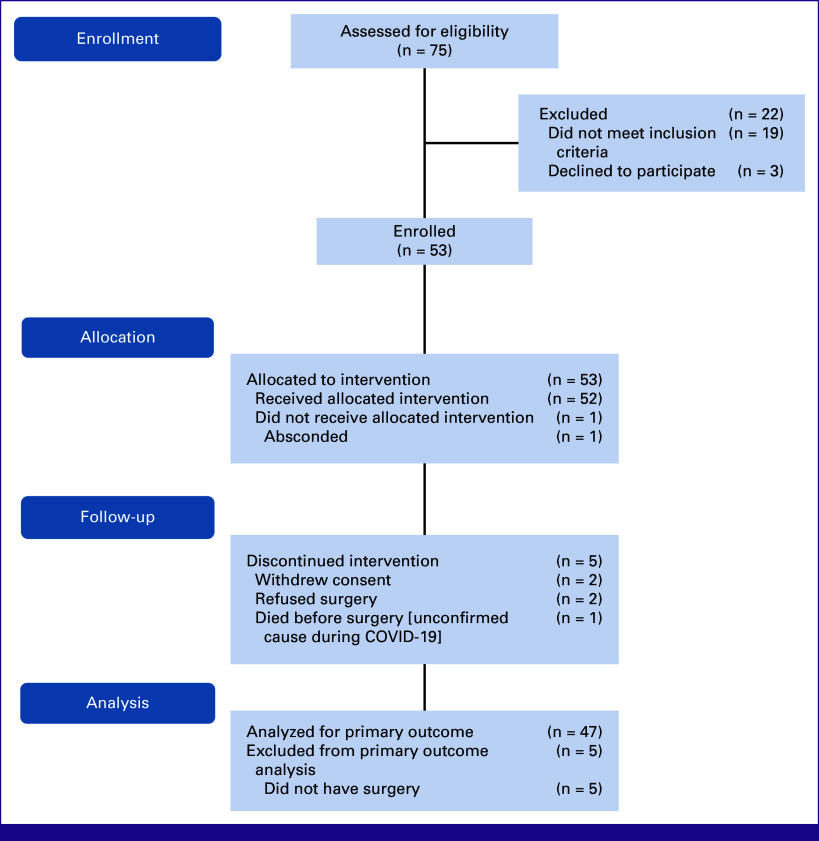
Participants enrollment diagram. Adapted from Hopewell et al.^33^

Between April 7, 2020, and August 5, 2022, we enrolled 53 female patients age 18-70 years. One participant did not receive any treatment, leaving a total of 52 patients. However, only 47 participants were evaluable because of two consent withdrawals, two surgery refusals, and one death from an unconfirmed cause before surgery during the COVID-19 pandemic. The study flowchart, using the adapted CONSORT format, is shown in Figure 1. The baseline demographic and clinical characteristics of the patients are shown in Table 1. The mean age of the patients was 47.6 years (range, 25-70 years). There were few comorbidities, mainly well-controlled hypertension. Forty-two percent of the patients were ER+ and 40% PR+. All the patients had an ECOG performance status score of 0. The mean BMI was 28 (range, 17-41). Forty percent of the patients were anatomic stage II and 60% were stage III. Fifty-three percent of the cancers were infiltrating ductal, 28% ductal/lobular, 15% invasive lobular, and 4% papillary or other.

**TABLE 1:** Baseline Demographic and Clinical Characteristics (N = 47)

Characteristic	No.	No. Missing (If >0)
Age (mean, SD, range)	47.6, 10.6, 25-70	
BMI (mean, SD, range)	28, 5.3, 17-41	
BSA (mean, SD, range)	1.80, 0.18, 1.46-2.14	
Primary school, No. (%)	5 (10.6)	
Secondary school, No. (%)	15 (31.9)	
Vocational/technical school, No. (%)	1 (2.1)	
Polytechnic, OND, or some college, No. (%)	7 (14.9)	
Bachelor's degree, No. (%)	14 (30)	
Postgraduate degree, No. (%)	5 (10.6)	
Married, No. (%)	30 (63.8)	
Separated, No. (%)	9 (19.2)	
Never married, No. (%)	4 (8.5)	
Widowed, No. (%)	4 (8.5)	
Christian, No. (%)	41 (87.2)	
Muslim, No. (%)	6 (12.8)	
Yoruba, No. (%)	32 (68.1)	
Ibo, No. (%)	9 (19.2)	
Other, No. (%)	6 (12.8)	
ER positive, No. (%)	19 (42.2)	2
PR positive, No. (%)	18 (40)	2
HER2 positive, No. (%)	47 (100)	
Histology, No. (%)		
Ductal/lobular	13 (27.7)	
Infiltrating ductal	25 (53.2)	
Invasive lobular	7 (14.9)	
Papillary	1 (2.1)	
Other	1 (2.1)	
Stage, No. (%)		2
IIA	8 (17.8)	
IIB	10 (22.2)	
IIIA	13 (28.9)	
IIIB	9 (20)	
IIIC	5 (11.1)	

Abbreviations: BSA, body surface area; ER, estrogen receptor; HER2, human epidermal growth factor receptor 2; OND ordinary national diploma; PR, progesterone receptor; SD, standard deviation.

Of the 47 evaluable patients, 25 achieved pCR, whereas 22 did not. Thus, the number of pCRs required to reject the null hypothesis (H_0_: pCR = 20%) was exceeded, with an observed pCR rate of 53.2% ([95% CI, 38.1 to 67.9]; *P* < .001). The obtained pCR surpassed the planned cutoff for trial success of 40%. Six patients (12.8%) achieved pCR with docetaxel + trastuzumab, and the 19 remaining pathologic complete responders received FEC as well.

The pCR according to ER status, PR status, lymph nodes (based on physical examination), and histology is tabulated in Table 2. The association between cancer stage and pCR was not significant (*P* = .075). The variation in pCR according to tumor grade was also not statistically significant (*P* = .91). During neoadjuvant therapy, there were no reported grade 4 or 5 toxicities. Six patients (12.8%) experienced grade 3 myelosuppression (anemia, neutropenia, WBC decrease, or platelet count decrease) during neoadjuvant or adjuvant treatment. One patient (2.1%) experienced grade 3 diarrhea during neoadjuvant therapy, and two patients (4.2%) developed hepatitis while receiving trastuzumab medications during the adjuvant phase. One of the two patients with hepatitis tested positive for hepatitis B virus (HBV) and was administered appropriate antiviral therapy. Reduced left ventricular ejection fraction (20%-38%) with no symptoms was observed in five patients (10.6%), and pericardial effusion (mild) was observed in one patient (2.1%). None of the ejection fractions were <50% (Table 3). All patients recovered and completed treatment according to the protocol. No treatment-related discontinuation was observed. One patient died at home from an unconfirmed cause before surgery during the COVID-19 pandemic, and an autopsy could not be performed to confirm the cause of death. Reports on exploratory end points such as iDFS and OS await future analysis.

**TABLE 2:** Pathologic Complete Response by Hormone Receptor Status, Lymph Nodes, Histology, Stage, and Grade

Variable	No.	pCR Rate, No. (%), [95% CI]	*P*
ER status			
Negative	26	16 (61.5), [40.6 to 79.8]	.24
Positive	19	8 (42.1), [20.3 to 66.5]	
PR status			
Negative	27	16 (59.3), [38.8 to 77.6]	.37
Positive	18	8 (44.4), [21.5 to 69.2]	
Lymph nodes			
Normal	29	16 (55.2), [35.7 to 73.6]	.77
Abnormal	18	9 (50), [26 to 74]	
Histology			
Infiltrating ductal	25	11 (44), [24.4 to 65.1]	.18
Invasive lobular	7	6 (85.7), [42.1 to 99.6]	
Ductal/lobular	13	6 (46.2), [19.2 to 74.9]	
Papillary	1	1 (100), [2.5 to 100]	
Other	1	1 (100), [2.5 to 100]	
Clinical stage			
IIA	8	3 (37.5), [8.5 to 75.5]	.75
IIB	10	10 (30), [6.7 to 65.2]	
IIIA	13	10 (76.9), [46.2 to 95]	
IIIB	9	6 (66.7), [29.9 to 92.5]	
IIIC	5	1 (20), [0.5 to 71.6]	
Grade			
I	5	3 (60), [14.7 to 94.7]	.91
II	24	12 (50), [29.1 to 70.9]	
III	17	10 (58.8), [32.9 to 81.6]	

Abbreviations: ER, estrogen receptor; pCR, pathologic complete response; PR, progesterone receptor.

**TABLE 3:** Toxicity Profile (neoadjuvant phase)

Toxicity	No. of Patients	Percentage of Patients
Grade 4 or 5 toxicities	0	0
Grade 3 myelosuppression (anemia, neutropenia, WBC or platelet decrease)	6	12.8
Grade 3 diarrhea	1	2.1
Hepatitis (on study medications)	2	4.2
Reduced left ventricular ejection fraction (20%-38%)	5	10.6
Pericardial effusion (mild)	1	2.1
Ejection fraction <50%	0	0

## DISCUSSION

The ARETTA 1.0 study evaluated the impact of biomarker-driven therapy on pCR rates in Nigerian women with HER2-positive clinically nonmetastatic breast cancer. This trial demonstrated the efficacy and feasibility of combining chemotherapy with HER2-targeted therapy, using trastuzumab alone. The trial achieved a pCR rate of 53% ([95% CI, 38.1 to 67.9]; *P* < .001), which surpassed the planned cutoff for success of 40%. In a phase II study of the TECHNO trial, a pCR of 39% of 217 enrolled participants was achieved following three weekly cycles of cyclophosphamide, epirubicin, docetaxel, and trastuzumab.^18^ In another randomized study report from the MD Anderson Cancer Center trial, patients with HER2-positive breast cancer received paclitaxel followed by FEC with or without concurrent trastuzumab. The pathologic complete response rate increased from 26% to 65% (*P* = .02) after trastuzumab treatment. There were few differences in toxicity rates.^19,20^

The application of anthracyclines in early-stage HER2-positive breast cancer has been restricted in recent years, owing to potential long-term cardiac toxicity.^21,22^ In a previous report, cardiac function was assessed in an adjuvant study with anthracycline-based chemotherapy, and trastuzumab showed an increased incidence of cardiac adverse events in the combination arm; however, the overall risk-benefit assessment favored the combination with trastuzumab.^23^ In the phase II randomized TRYPHAENA cardiac safety trial, various chemo backbones with trastuzumab and pertuzumab, acceptable cardiac safety, supported concurrent anthracycline-containing and anthracycline-free choices. Specifically, only one of 223 patients (0.4%) who received trastuzumab and pertuzumab in combination with standard chemotherapy concurrently developed symptomatic left ventricular systolic dysfunction during the neoadjuvant treatment period.^24^ Although contemporary practice is in favor of anthracycline-free approaches, we planned to treat our patient with neoadjuvant docetaxel and trastuzumab. In our protocol, docetaxel and trastuzumab were administered followed by FEC if the patient did not achieve CCR after the first phase of treatment. The goal of this strategy was to evaluate the feasibility of treatment de-escalation, balancing efficacy and quality of life against the potential for increased toxicity associated with more aggressive regimens. In the event of an unsatisfactory response, especially in our setting with predominantly locally advanced disease, FEC was added sequentially to optimize chemotherapy treatment, as dual HER2-receptor blockade with pertuzumab was not readily available. Under this plan, trastuzumab administration could not be interrupted until after the FEC chemotherapy. To ameliorate cardiotoxicity, only three courses of FEC were administered following the regimen used in one of the TRYPHAENA arms, which had a combination of trastuzumab and pertuzumab with reported low cardiotoxicity. We presumed that the risk-benefit ratio would favor this combination.

The use of dual HER2 blockade with docetaxel alone docetaxel, trastuzumab and pertuzumab (DTP) resulted in a pCR of 46%.^24^ Our corresponding figure with docetaxel and trastuzumab alone showed 12.8% pCR, showing the advantage of dual blockade. Dual HER2 blockade with trastuzumab and pertuzumab with anthracycline (FEC × 3 → DTP) and non–anthracycline-containing regimen consisting of docetaxel, cyclophoasphamide, trastuzumab and pertuzumab (DCTP) achieved pCR rates of 57%-66%.^24^ Our regimen resulted in a pCR of 53%, which is still acceptable, especially when 60% of our patients had locally advanced diseases and used trastuzumab alone. The SC fixed dose of trastuzumab was well tolerated and accepted. With this experience, the use of the SC fixed-dose pertuzumab-trastuzumab combination, when widely available, will improve local practice. The minimal staging imaging procedures used were chest radiography and ultrasonography of the abdomen and pelvis, as computed tomography (CT) facilities were not uniformly available. The trial also revealed facility limitations. These barriers continue to hinder comprehensive cancer care in Nigeria.^25^

The ARETTA 1.0 findings generated a hypothesis that can guide phase III study designs on the efficacy of HER2 targeted therapies in patients with HER2-positive breast cancer in Nigeria to compare outcomes with other populations. Achieving pCR is associated with better long-term outcomes including improved OS.^26^ An initial pCR rate of approximately 13% after 12 weeks of docetaxel and trastuzumab treatment suggested that de-escalation was not practical. This finding reinforces previous findings that multiagent chemotherapy, rather than a single agent, is associated with higher pCR rates.^27^

The treatment regimen was well tolerated (Table 3). This aligns with known adverse effects of the FEC regimen.^28^ Despite concurrent use of FEC and trastuzumab, no instances of symptomatic heart toxicity were observed. These findings suggest that with careful patient selection and vigilant cardiac monitoring, inclusion of FEC with concurrent trastuzumab is feasible. Therefore, this trastuzumab-only regimen may be important for Sub-Saharan Africa where pertuzumab or other HER2 blockers are not readily available. The development of hepatic toxicity during the adjuvant phase of trastuzumab treatment in two participants adds to the rare occurrence of hepatic toxicity observed with trastuzumab use in breast cancer. A search of the literature on trastuzumab-induced hepatotoxicity identified only four such reports.^29^ Given the two hepatitis cases, we recommend baseline HBV screening and antiviral prophylaxis where indicated. Baseline screening for HBV for all patients with cancer is recommended using triple screening methods for HBV, following the recommendations of the US CDC.^30^ The ASCO Clinical Oncology guidelines recommend that HBsAg-positive and anti–HBc-positive patients should receive antiviral prophylaxis during systemic therapy and for 12 months after treatment.^31^ The strength of this study lies in its pragmatic design and successful implementation, despite the challenges of the COVID-19 pandemic.

The limitations of this study include its nonrandomized nature and modest sample size, which limit definitive comparative conclusions. HER2 testing was limited to IHC 3+, which excludes those with equivocal results that might have tested HER2 positives by the FISH technique, potential misstaging because of limited imaging access to CT-based staging and the exclusion of HIV-positive patients in the setting where HIV epidemiology is relevant, as well as short time-to-reporting for iDFS/OS as survival outcome reports await future analyses.

Toward the advancement of oncology care in Nigeria, future directions should include using CT as a minimum staging modality and exploring the use of liquid biopsies to transform patient monitoring.^32^ The use of dual HER2 blockade with trastuzumab and pertuzumab to study efficacy and toxicity in the study population is warranted.

In conclusion, this trial demonstrated that neoadjuvant docetaxel plus T + scH with adaptive FEC achieved a pCR rate of 53% in Nigerian female patients with HER2-positive breast cancer. This regimen was safe, highlighting its hypothesis-generating potential for further studies on HER2 targeted therapy in sub-Saharan Africa. Although the results compare favorably with similar trastuzumab-only regimen studies in high-income countries, the absence of pertuzumab limits direct comparison with other reports. Future directions include phase III studies, as well as integrating dual HER2 blockade where available, strengthening the diagnostic and staging capacity, and incorporating routine HBV screening.

## Data Availability

A data sharing statement provided by the authors is available with this article at DOI https://doi.org/10.1200/GO-25-00287.

## PREPRINT VERSION

Preprint version available at: http://dx.doi.org/10.2139/ssrn.5058460.
