# Supplementary material for: Pragmatic Clinical Trial Assessing Response to Neoadjuvant Docetaxel and Trastuzumab in Nigerian Women With Human Epidermal Growth Factor Receptor 2–Positive Breast Cancer (ARETTA)
Source: JCO Glob Oncol. 2026 May 29;12:e2500287. doi: 10.1200/GO-25-00287 (PMC13268114; doi:10.1200/GO-25-00287)
Supplement: Supplementary file 2 [file go-12-e2500287-s002.pdf]

<u>A</u>ssessing <u>RE</u>sponse to neoadjuvant <u>T</u>axotere and <u>T</u>r<u>A</u>stuzumab in Nigerian women with HER2-positive breast cancer (ARETTA)

Document type	Oncology Synopsis
EUDRACT number	Not applicable
Study phase	II
Document status	Final
Release date	June 29, 2020
Revisions	Version 2

SPONSOR: University of Chicago Center for Global Health (UC CGH)

Participating Centers:

University College Hospital, Ibadan, Nigeria (UCH Ibadan)
Lagos University Teaching Hospital (LUTH) Idi-Araba Lagos
Lagos State University Teaching Hospital, Ikeja, Lagos State, Nigeria (LASUTH Lagos)
Obafemi Awolowo University Teaching Hospitals Complex, Ile Ife, Nigeria (OAUTHC Ile Ife)
University of Chicago Medical Center, Chicago, IL, USA (UCMC Chicago)

Signatures:

Investigator

I have read and agree to the protocol '**Assessing REsponse rate to neoadjuvant Taxotere and TrAstuzumab in Nigerian women with HER2-positive breast cancer** '(Version____, dated__________). I am aware of my responsibilities as an Investigator under the guidelines of GCP, local regulations (as applicable) and the study protocol. I agree to conduct the study according to these responsibilities and to appropriately direct and assist the staff under my control, who will be involved in the study.

Signatures:

Investigator

I have read and agree to the protocol '**Assessing REsponse rate to neoadjuvant Taxotere and TrAstuzumab in Nigerian women with HER2-positive breast cancer** '(Version_____, dated_). I am aware of my responsibilities as an Investigator under the guidelines of GCP, local regulations (as applicable) and the study protocol. I agree to conduct the study according to these responsibilities and to appropriately direct and assist the staff under my control, who will be involved in the study.

Table of Contents

Participating Centers 2
List of abbreviations 22
Glossary of terms 25
Protocol summary 28
1 Introduction 31
1.1 Breast cancer overview 31
1.2 Breast cancer burden in Nigeria and Sub-Saharan Africa 31
1.3 Treatment options for breast cancer 33
1.4 Neoadjuvant chemotherapy 33
1.5 Introduction to investigational treatment(s) and other study treatment(s) 34
1.5.1 Docetaxel (TXT) 34
1.5.2 5-Fluorouracil, Epirubicin, Cyclophosphamide (FEC) 35
1.5.3. Herceptin (Trastuzumab) (H) 35
1.5.5 Tamoxifen (TAM), letrozole (LET) and Luteinizing Hormone Releasing Hormone (LHRH) agonist. 36
2 Rationale 36
2.1 Study rationale and purpose 36
2.2 Rationale for choice of drug and schedule 38
2.2.1 Docetaxel (TXT) and Trastuzumab 38
3. Objectives and endpoints 39
4. Study design 45
4.1 Overview 45
4.2 Definition of end of study 47
4.3 Early study termination 47
5. Patient population 47
5.1 Inclusion criteria 48
5.2 Exclusion criteria 48
5.3 Recruitment process 49
6. Treatment 50

6.1 Study treatment 50
6.2 Dosing guidelines 51
6.3 Ultrasound evaluation of response 52
6.4 Dosing regimen 52
6.5 Treatment duration 52
6.6 Pretreatment considerations: 53
6.7 Guidelines for continuation of treatment 54
6.8 Dose modification and dose delay 54
AST or ALT 58
AST or ALT and Bilirubin 58
6.9 Disease progression during study 61
6.10 Follow-up for toxicities 61
6.11 Anticipated risks and safety concerns of the study treatments 61
6.12 Concomitant medications 61
6.13 Prohibited concomitant therapy 61
6.14 Patient numbering 62
6.14.1 Patient numbering 62
6.14.2 Treatment assignment 62
6.14.3 Treatment blinding 62
6.15 Study drug preparation and dispensation 62
6.16 Drug supply and storage 62
6.17 Study drug accountability and compliance 63
7. Study flow and visits schedule 63
7.1 Screening 63
Bone scan 68
CT scan of brain 69
CT scan of chest, abdomen, pelvis 69
7.1.2 HLF Data Submission 72
7.1.4 Patient demographics and other baseline characteristics 74
7.2 Treatment period 74
7.3 End of treatment visit including study completion and premature withdrawal 74
7.4 Criteria for premature patient withdrawal 74

7.4.1 Replacement policy ... 75
7.5 Follow up period ... 75
7.5.1 30 - day safety follow up period ... 75
7.5.2 Survival follow up period ... 75
7.6 Assessment types ... 75
7.6.1 Efficacy assessments ... 75
7.6.2 Safety and tolerability assessments ... 76
7.7 Patient reported outcomes ... 78
7.7.1 The EORTC Quality of Life questionnaire ... 78
8. Safety monitoring and reporting ... 78
8.1 Adverse events ... 78
8.1.1 Definitions ... 78
8.2 Serious adverse event ... 80
8.2.1 Definition ... 80
8.2.2 Reporting ... 80
8.3 Pregnancies ... 81
8.4 Routine Adverse Event Reporting ... 81
8.5 Serious Adverse Event Reporting to the Coordinating Center ... 83
8.6 Serious and Unexpected Adverse Event reporting by the Coordinating Center ... 83
8.7 Warnings and precautions ... 84
9. Statistical methods and data analysis ... 84
9.1 Analysis sets ... 84
9.1.1 Full Analysis Set (FAS) ... 84
9.1.2 Safety Set ... 84
9.2 Patient demographics and other baseline characteristics ... 84
9.3 Treatment (study treatment, concomitant therapies, compliance) ... 85
9.4 Primary objective ... 85
9.5 Primary Outcome Measure ... 85
9.6 Handling the missing values; censoring and discontinuations ... 86
9.7 Secondary objectives ... 86
9.8 Secondary efficacy objectives ... 86
9.8.1 Invasive disease-free survival (iDFS) ... 86

9.8.2 Subgroup analysis ... 87
9.9 Safety objectives ... 87
9.9.1 Analysis set and grouping for the analysis ... 87
9.9.2 Adverse events (AEs) ... 87
9.9.3 Laboratory abnormalities ... 88
9.9.4 Other safety data ... 88
9.10 Patient-reported outcomes ... 89
10 Biospecimens management and biomarker analysis ... 89
10.1 **Biospecimens** ... 89
10.2 **Pathology consideration** ... 90
10.3 **Biomarkers, correlative and special studies** ... 90
11 STUDY MANAGEMENT AND REGULATORY AFFAIRS ... 91
11.1 Multicenter Guidelines ... 91
11.2 Institutional Review Board (IRB) Approval and Consent ... 91
11.3 Required Documentation ... 92
11.4 Data and Safety Monitoring ... 92
11.5 Data Safety Monitoring Board ... 94
11.6 Auditing ... 94
11.7 Amendments to the Protocol ... 94
11.8 Annual IRB Renewals, Continuing Review and Final Reports ... 95
11.9 Record Retention ... 95
11.10 Obligations of Study Site Investigators ... 95
11.11 **Study finance and sponsorship** ... 96
11.12 **Steering Committee** ... 96
11.13 **Coordinating Center and Sponsor** ... 97
12 References (hard copy available upon request) ... 97
APPENDICES ... 105
APPENDIX A. ECOG Performance Status ... 105
APPENDIX B. Response Evaluation Criteria In Solid Tumors, RECIST (adapted from Version 1.1) ... 106
APPENDIX C. Quality of Life (QoL) Assessment ... 107
APPENDIX D. Responsibilities of the Protocol Steering Committee Chair (Protocol

Chair) and Coordinating Center 112

APPENDIX E. Asymptomatic decline in LVEF: Algorithm for Continuation and Discontinuation of HER2-Targeted Study Medication 113

APPENDIX F. Criteria for New York Heart Association Functional Classification 114

APPENDIX G. Nomogram for the Determination of Body Surface Area 115

APPENDIX H. ARETTA study advert 116

LIST OF FIGURES

Figure 1-1 Spectrum of breast cancer subtypes across populations… 32
Figure 4-1 Study Schema Part 1: Neoadjuvant Treatment… 46
Figure 4-2 Study Schema Part 2: Adjuvant Treatment.. 47

LIST OF TABLES

Table 3-1 Objectives and related endpoints 40
Table 6-1 Dose and treatment schedule 52
Table 6-2 Recommendations for dose modification for docetaxel or FEC by event........... 57
Table 6-3 Recommendations for dose modification by the appearance of the adverse drug reaction 60
Table 7-1 Visit evaluation schedule (Neo-Adjuvant) (Part 1). Protocol Section 7.2........... 64
Table 7-2 Visit evaluation schedule (Part 2) (Adjuvant Therapy) for Complete/Partial Responders/SD (operable) Protocol Section 7.2… 68
Table 7-3 Visit evaluation schedule- End of treatment visit and follow up......................... 70
Table 8-1 Reporting Conventions for Left Ventricular Systolic Dysfunction/Heart Failure... 82

List of abbreviations

AC	Adriamycin/cyclophosphamide
AE	Adverse Event
AI	Aromatase inhibitor
AJCC	American Joint Committee on Cancer
ALT	Alanine aminotransferase/glutamic pyruvic transaminase/GPT
ANC	Absolute neutrophil count
AST	Aspartate aminotransferase/glutamic oxaloacetic transaminase/GOT
ATC	Anthracycline
BHGI	Breast Health Global Initiative
BRCA1	Breast cancer gene 1
BUN	Blood urea nitrogen
CAF (FAC)	Cyclophosphamide, doxorubicin, 5-fluorouracil
CBC	Complete Blood Count
CCR	Core clinical report
CCSR	Core clinical study report
CDP/CIS-DDP	Cis-diamminedichloroplatinum (Cisplatin)
CI	Confidence interval
CISH	Chromogenic *in situ* hybridization
CKD EPI	Chronic Kidney Disease Epidemiology Collaboration
CMF	Cyclophosphamide, methotrexate, 5-fluorouracil
C1D1	Cycle 1, day 1
CPM	Cyclophosphamide
CRF	Case Report/Record Form; the term CRF can be applied to either EDC or paper hard copy
CSP	Clinical study protocol
CSR	Clinical study report
CSR addendum	An addendum to clinical study report (CSR) that captures all the additional information that is not included in the CSR
CTC	Common Toxicity Criteria
CTCAE	Common Terminology Criteria for Adverse Events_v 4.0
CTPF	Clinical trial pregnancy form
CYP	Cytochrome
DAR	Dosage administration record
DBC	Differential blood count
DF	Disease-free
DHFR	Dihydrofolate reductase

DOR	Duration of response
DQF	Data query form
DS&E	Drug safety and epidemiology
DTX/DOC	Docetaxel (Taxotere)
D5W	Dextrose 5% in water solution
EC	Epirubicin
ECG	Electrocardiogram
ECHO	Echocardiography
ECOG PS	Eastern Cooperative Oncology Group performance status
EOT	End of Treatment
EORTC	European Organization for Research and Treatment of Cancer
QLQ-30	EORTC quality of life questionnaire
E&U+CR	Electrolytes, urea and creatinine
ER	Estrogen receptor
FAS	Full analysis set
FCSR	Final clinical study report
FDA	Food and Drug administration
FEC	5-Fluorouracil, Epirubucin and Cyclophosphamide
FISH	Fluorescence *in situ* hybridization
F/U	Follow up period
GCP	Good clinical practice
H	(Herceptin) trastuzumab
HLF	Healthy Life for all Foundation
HER2	Human epidermal growth factor receptor 2
HIV	Human immunodeficiency virus
HPF	High power field (in microscopy)
HR+	Hormone receptors positive
i.v. or IV	Intravenous
ICF	Informed consent form
ICH	International Conference on Harmonization
IEC	Independent Ethics Committee
IHC	Immunohistochemistry
IRB	Institutional Review Board
IUCD	Intrauterine contraceptive device
LABC	Locally advanced breast cancer
LET	Letrozole
LFT	Liver function tests
LHRH	Luteinizing hormone-releasing hormone
LLN	Lower limit of normal
LN	Lymph node
LPLV	Last patient last visit
LVEF	Left ventricular ejection fraction

MedDRA	Medical dictionary for regulatory activities
MTS	Metastasis
NAFDAC	National Agency for Food and Drug Administration and Control
NCAIDs	Non-steroidal anti-inflammatory drugs
NCCN	National Comprehensive Cancer Network (An alliance of 26 cancer centers in the USA)
NCI	National Cancer Institute
NIBMR	Novartis Institute for Biomedical Research Inc
NS	Normal saline solution
ORR	Objective response rate
OS	Overall survival
pCR	Pathologic complete response
piCR	Pathologic incomplete response
PD	Progressive disease
PI	Principal investigator
PFS	Progression-free survival
p.o.	*per os*/by mouth/orally
PHI	Protected Health Information
PK	Pharmacokinetics
PLT	Platelet
PPS	Per-protocol set
Pt(s)	Patient(s)
PgR	Progesterone receptor
PRF	Patient registration form
QoL	Quality of life
REB	Research Ethics Board
RECIST	Response Evaluation Criteria in Solid Tumors
RR	Response rate
SAE	Serious adverse event
s.c. or SC	Sub-cutaneous
SD	Stable disease
SEC	Specific safety event categories
SGOT	Serum glutamic-oxaloacetic transaminase
SGPT	Serum glutamic pyruvate transaminase
SIADH	Syndrome of inappropriate anti-diuretic hormone
SOC	Standard of care
SOP	Standard operating procedure

SUSAR	Suspected unexpected serious adverse reactions
TAM	Tamoxifen
TTP	Time to progression
UCMC	University of Chicago Medical Center
UC CGH	University of Chicago Center for Global Health
ULN	Upper limit of the normal range
USS	Ultrasound scanning
WBC	White blood count

Glossary of terms

Term	Definition
Assessment	A procedure used to generate data required by the study
Cycles	Number and timing or recommended repetitions of therapy are usually expressed as number of days (e.g. q28 days)
Complete Response (CR)	Defined by RECIST criteria disappearance of all lesions
Dose intensity	Computed as the ratio of total dose received and actual duration. The relative dose intensity computed as the ratio of dose intensity and planned dose received/planned duration
Enrollment	Point/time of patient entry into the study; the point at which informed consent must be obtained (i.e. prior to starting any of the procedures described in the protocol)
Human epidermal growth factor receptor (HER2) positive tumor	Determined by at least one HER2 test (IHC, FISH, CISH) according to the FDA USA and manufacturer (DAKO Inc) and ASCO-CAP HER2 Test 2007 Guideline and 2013-2014 Recommendations. www.asco.org/guidelines/her2
Hormone receptor (HR) positive tumor	Determined by IHC test of Estrogen Receptor (ER) and Progesterone Receptor (PgR) statuses according to the FDA USA and manufacturer (DAKO Inc) and ASCO-CAP ER/PgR Guidelines http://www.instituteforquality.org/asco-cap-guideline-recommendations-immunohistochemical-testing-estrogen-and-progesterone-receptors
Investigational treatment	Drug whose properties are being tested in the study as well as their associated placebo and active treatment controls (when applicable). This also includes approved drugs used outside of their indication/approved dosage, or that are tested in a fixed combination. Investigational treatment generally does not include other study treatments administered as concomitant background therapy required or allowed by the protocol when used according to approved indication/dosage

Objective Response Rate (ORR)	The proportion of patients with an overall response of CR or PR, where tumor response is based on change in tumor diameter on USS after 4 cycles of three-weekly docetaxel in combination with HER-2 targeted therapy
Other study treatment	Any drug administered to the patient as part of the required study procedures that was not included in the investigational treatment
Palliative treatment	Palliative treatment focuses on improving the quality of life of patients with serious illnesses. It helps not only to reduce pain in patient's body or treatment side effects, but also in mind and spirit. Palliative care may be combined with curative treatment. Palliative care team includes palliative care doctors, nurses, and social workers, spiritual advisors, dietitians, occupational therapists, pharmacists, physical therapists, respiratory therapists, and volunteers
Patient Number (Patient No.) Subject Number (Subject No.)	A unique identifying number assigned to each patient/subject/healthy volunteer who enrolls in the study
Partial Response (PR)	≥ 30% decrease in the longest diameter of target lesions, with the baseline measurements taken as the reference
Partial Response inoperable (PRi)	Disease that meets criteria for partial response following ultrasound assessment after 4 courses of docetaxel but still surgically assessed as inoperable based on residual tumour size
Partial Response operable (PRo)	Disease that meets criteria for PR and is surgically assessed as operable based on residual tumour size
Pathologic Complete Response (pCR)	Histo-pathological examination of surgical specimen describes no gross or microscopic disease
Pathological incomplete response (piCR)	If there is residual or microscopic disease on histo-pathological examination of surgical specimen
Performance status	Measure of cancer patients' general well being and activities of daily life to determine whether they can receive chemotherapy, whether dose adjustment is necessary, and as a measure for the required intensity of palliative care. It is also used as a measure of quality of life. 0 = normal activity, asymptomatic; 1 = symptomatic, fully ambulatory; 2 = symptomatic, in bed < 50% of time; 3 = symptomatic, in bed > 50% of time; 4 = 100% bedridden
Pharmacokinetics (PK)	The process by which a drug is absorbed, distributed, metabolized, and eliminated by the body

Premature patient withdrawal	Point/time when the patient exits from the study prior to the planned completion of all study treatment administration and/or assessments; at this time all study treatment administration is discontinued and no further assessments are planned, unless the patient will be followed for progression and/or survival
Progression Free Survival (PFS)	Defined as time from first dose of study drug treatment to progression or death due to any cause, as assessed by investigators
State of menopause	Considered post-menopausal and not having child bearing potential women if they have had 12 months of natural (spontaneous) amenorrhea with an appropriate clinical profile (e.g. age appropriate, history of vasomotor symptoms) or have had surgical bilateral oophorectomy (with or without hysterectomy) or tubal ligation at least six weeks before entering the study. In the case of oophorectomy alone, only when the reproductive status of the woman has been confirmed by follow up hormone level assessment is she considered not of child bearing potential
Response Rate (RR)	Proportion of pts with change (shrinkage of at least 30%) in tumor diameter detected by breast ultrasonography
Stage related to study timeline	A major subdivision of the study timeline; begins and ends with major study milestones such as enrollment, randomization, completion of treatment, etc.
Safety assessment	The measures used to assess the toxicity level e.g. blood tests, ECHO
Stop study participation	Point/time at which the patient came in for a final evaluation visit or when study treatment was discontinued whichever is later
Study treatment	Includes any drug or combination of drugs in any study arm administered to the patient (subject) as part of the required study procedures, including placebo and active drug run-ins.<br>In specific examples, it is important to judge investigational treatment component relationship relative to a study treatment combination; study treatment in this case refers to the investigational and non-investigational treatments in combination
Study treatment discontinuation	Point/time when patient permanently stops taking study treatment for any reason; may or may not also be the point/time of premature patient withdrawal
Systemic toxicity	Adverse effects caused by a substance that affects the body in a general rather than local manner

Variable	Item or field used in the data analysis; derived directly or indirectly from data collected using specified assessments at specified time points
Vital signs	Defined by measuring the heart rate, blood pressure and temperature

Protocol summary

Protocol number	IRB 18-1178<br>CABC123D4567
Title	Assessing REsponse to neoadjuvant Taxotere and TrAstuzumab in Nigerian women with HER2-positive breast cancer (ARETTA)
Clinical phase	II
Investigation type	Drug
Study type	Interventional
Purpose and rationale	This investigator-initiated study was co-developed with the Ibadan multidisciplinary breast cancer group to examine pathologic complete response rate to neoadjuvant chemotherapy in combination with HER2 blockage therapy among Nigerian women with HER2-positive breast cancer. Our objective is to examine what proportion of patients will achieve complete pathological response with docetaxel + subcutaneous Herceptin, potentially supplemented with FEC, and be spared toxicity of additional chemotherapy.
Primary objective and key secondary objective	To test the efficacy and toxicity of three-weekly docetaxel chemotherapy and HER2 blockage with SC Herceptin (TscH) in Nigerian women with early stage HER2-positive breast cancer. The primary endpoint is complete pathologic response (pcR) after four cycles (12 weeks) of T + scH. Responding patients with stable disease or partial response after TscH X 4 will receive neo-adjuvant non-cross resistant treatment with FEC +scH X 3. All patients who achieve pCR will receive Herceptin SC post surgery to complete one year. A key secondary objective is to assess invasive disease-free survival (iDFS) at one year from enrollment.
Additional secondary objectives	To determine the pattern of response and mechanism of resistance to three-weekly TH based on genomic biomarkers including hormone receptors (ER and PgR) . To determine the pharmacokinetics of Herceptin SC. To assess the quality of life (QoL) of the patients during and after treatment. To determine the cardiac toxicity associated with TscH with or without FEC +scH in Nigerian patients with breast cancer.

Study design	This is a one-stage phase II study with a single arm design. The primary endpoint is rate of complete pathologic response (pCR). All patients will receive 4 cycles of T+ scH in the neo-adjuvant setting. Those with stable disease or partial response and operable disease will receive FEC+scHP for an additional 3 doses in the neoadjuvant setting. After surgery, all patients who achieve pCR will receive one year of Herceptin SC to complete 18 cycles. Patients with pathological incomplete response may have additional chemotherapy at the discretion of the managing physician. However, patients who are inoperable or have progressive disease after evaluation by ultrasound following 4 cycles of T+ scH will go off study treatment, as will those who are inoperable or have progressive disease after FEC. All enrolled patients will be followed for overall survival.<br>Adjuvant treatment will include hormonal therapy for hormone receptor positive disease. Radiation therapy will be administered per standard of care. All patients will have active follow up every three months and have routine blood tests and physical examination for the first two years after all treatment is completed and every six months for 10 years. CT scan and bone scan will be done as clinically indicated to evaluate recurrence. Toxicity and QoL will be assessed.
Study population	The study will be carried out among histologically confirmed HER2-positive breast cancer patients in Nigeria who are chemotherapy/hormonal treatment naïve
Inclusion criteria	Histologically confirmed carcinoma of the breast, AJCC stages IIA-IIIC. Females 18-70 years, with consent for the study. See Section 5.1 for complete list.
Exclusion criteria	Pregnancy, HER2-negative disease, patients with distant metastasis (brain or visceral disease). See Section 5.2 for complete list.
Investigational and reference therapy	Docetaxel, Trastuzumab (Herceptin SC) [TH] are the investigational treatments while 5-Flurouracil, Epirubicin, Cyclophosphamide, Tamoxifen/Letrozole, LHRH agonist (Zoladex) are adjunct standard therapy
Efficacy assessments	Complete pathologic response (pCR), breast USS response, invasive disease-free survival (iDFS)
Safety assessments	Systemic toxicity according to Common Terminology Criteria for Adverse Events (CTCAE) version 5.0, ECHO, vital signs, CBC, LFT, E&U+ CR
Other assessments	QoL using validated questionnaires. Genomic analysis of residual tumor and serum biomarkers including cfDNA.
Statistical analysis	All patients who signed consent and initiated neoadjuvant therapy will be included in the analysis. Demographics and baseline features will be presented using descriptive statistics. Based on current review of

	the literature in other populations, for this study we expect an inefficient treatment regimen of T+scH to elicit 20% or fewer pCR (p_0 = 0.20) and a successful treatment regimen to have at least 40% pCR at surgery (p_1 = 0.40). The patients with stable disease or partial response who attain pCR after additional FEC X 3 will be counted as successes, whereas those who are declared inoperable or have PD will be considered failures. A one-sided binomial test will be performed to test the null hypothesis of a 20% pCR rate vs. a 40% alternative. Secondary analyes will compare pCR rates by hormone receptor status, lymph node status, and breast cancer subtype using Fisher's exact test. iDFS will be defined as the time from enrollment to first occurrence of any of the following events: recurrence of ipsilateral invasive breast tumor, recurrence of ipsilateral locoregional invasive disease, distant recurrence, contralateral invasive breast cancer, or death from any cause. iDFS will be estimated by the Kaplan-Meier method; prognostic factors will be identified using Cox regression. The frequency of toxicity by type and grade will be summarized. Mixed effects regression models will be fit to analyze QoL.
Key words	Breast cancer, Docetaxel, Herceptin, neoadjuvant treatment, response, Nigeria

1 Introduction

1.1 Breast cancer overview

Breast cancer accounts for 30% of globally diagnosed cancers and 16% of cancer related deaths. The incidence of breast cancer is higher in high-income countries when compared to low and middle-income countries; however the mortality rate is similar. This is due to the availability of better treatment in the high-income countries (WHO, 2009). In Nigeria, breast cancer is the most common female malignancy with a majority of cases presenting late with advanced disease - either locally advanced breast cancer or metastatic disease (Parkin et al, 2006.

Breast cancer is a heterogeneous disease with different cancer molecular subtypes and phenotypes (Perou et al, 2000) each of which requires a different treatment regimen and has different clinical outcomes (Andre et al, 2006). The most common subtypes have been defined by immunohistochemical assays of tumor specimens and have been classified by hormone receptors (HRs) positive or negative and human epidermal growth factor receptor 2 (HER2) positive or negative statuses. More recent work has begun to elucidate the genomic abnormalities in breast cancer subtypes and to correlate these subtypes with response to neo-adjuvant chemotherapy (Kim et al, 2010; Huber et al, 2009; Caudle et al, 2012). Patients with molecularly-defined luminal A breast cancer (largely HER2-negative and hormone receptors-positive (HER2−/HRs+) appear to have considerably better prognosis compared to all other groups while those with HER2+ subtypes show good response to trastuzumab.

The use of molecularly targeted therapy has dramatically impacted breast cancer death rates in the US but global disparities in breast cancer outcomes continue to widen. In their most recent analysis, DeSantis et al noted the remarkable strides in breast cancer screening and treatment with a decrease in breast cancer mortality by 36% from 1989 to 2012; representing 249,000 breast cancer deaths averted in US women. Examples of treatment breakthroughs include HER2-targeted therapies, which benefit all women with HER2-positive tumors (DeSantis et al, 2015).

1.2 Breast cancer burden in Nigeria and Sub-Saharan Africa

Research on the breast cancer burden in Africa has been rather limited due to incomplete data collection and lack of population-based registries. Nonetheless, data from the Ibadan Cancer Registry, one of the sites reporting for GLOBOCAN suggest that the incidence of breast cancer has been on the rise (GLOBOCAN, 2014; Ferlay et al, 2015; Anyanwu S, 2008). This could be attributed to increased life expectancy, improved infectious disease control as well as changes in lifestyle, diet, and physical activity level. The peak incidence age of breast cancer in West Africa (Nigeria) is at least a decade earlier compared to Caucasians (Adebamowo et al, 2000). This may be due to more aggressive histologies, which are common among younger age groups especially in black populations. The mean age of women with breast cancer reported from Ibadan is 48 years (Ihekwaba et al, 1992; Forae et al, 2014). Most breast cancer patients present with advanced disease and more than 80% of the Nigerian breast cancer patients present with locally advanced disease: stage III-IV, large tumor size, diffuse tumor,

multifocal, fungating, of high grade, with high number of LN involvement, MTS and necrosis (Forae et al, 2014). The reason for this late presentation is multifactorial in nature ranging from delayed diagnosis and religious belief to ill-informed perceptions about breast cancer (Odusanya et al, 2001; Pruitt et al, 2014). Socioeconomic problems cause many patients to seek cheaper herbal and spiritual treatment options, and fear of mastectomy often leads to late presentations (Ayaji et al, 2002; Busolo & Woodgate, 2014).

Our study in Nigeria and Senegal (West Africa) reported that the proportions of ER-positive, PgR-positive, and HER2-positive tumors were 24%, 20%, and 17%, respectively (Huo et al, 2009). Others have reported even higher proportions for the expression of ER (54%), PgR (50%), and HER2 (31%) in sub-Saharan breast cancer cases (Nwafor & Keshinro, 2015) while 20-30% of breast cancers are HER2-positive (Agboola et al., 2012).

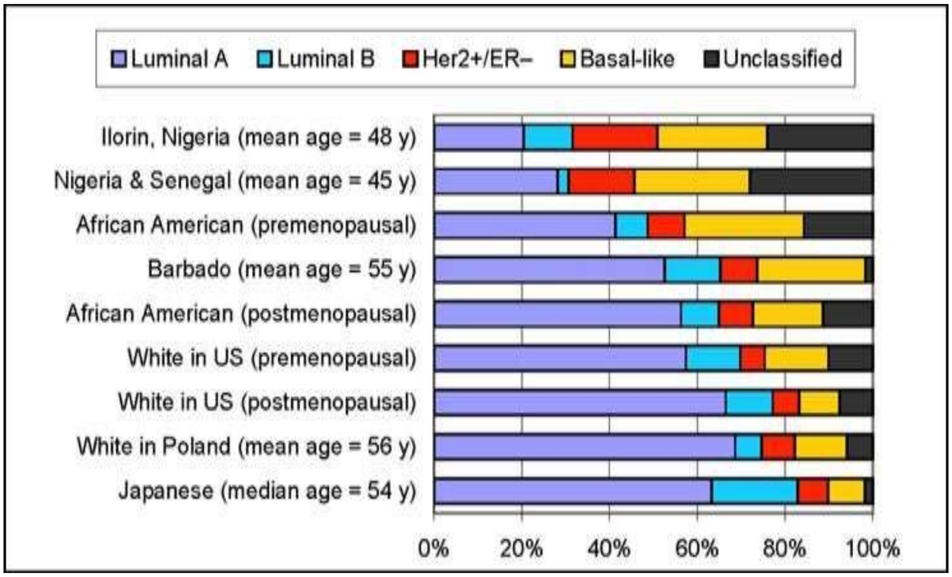

Figure 1.1. Spectrum of breast cancer subtypes across populations (Olopade et al, 2008; Huo et al, 2009)

Research over the past decade has revealed that the ratios of different molecular subtypes of breast cancer differ across populations (Figure 1.1). Results from the Carolina Breast Cancer Study (CBCS) showed that 39 percent of premenopausal African American women diagnosed with breast cancer had basal-like disease, compared with 14 percent of postmenopausal African American women and 16 percent of non-African American women of any age (Carey et al, 2006). A study from the California Cancer Registry also concluded that young African American women have a higher incidence of triple-negative breast cancer (TNBC) and also identified a smaller but significantly increased prevalence in Hispanic women (Bauer et al, 2007). Regardless of the stage at diagnosis, women with TNBC showed poorer survival than those with other breast cancers. Non-Hispanic black women had the poorest survival of any comparable group with a 5-year relative survival of 14%, compared to 36% of non-Hispanic white women and 37% for Hispanic women.

1.3 Treatment options for breast cancer

Chemotherapy can be considered for patients with a tumor size > 1 cm or any size with high risk features such as high histological grade, HER2-positive, ER-negative, lympho-vascular invasion, node positive, ER-/PR-/HER2- triple negative disease and young age at onset (Mauri et al 2005; Mayer et al, 2015; Partridge et al, 2014; Gnant et al, 2015; Kaufmann et al, 2012). Chemotherapy also improves relapse-free interval when combined with endocrine therapy (in those indicated) especially in young premenopausal women (Taylor W, 2010).

Chemotherapy is commonly combined with surgery and radiotherapy. Although HER2 positive breast cancer is an aggressive form of the disease with poor prognosis, the use of targeted therapies in the form of HER blockage has greatly improved the prognosis of this disease (Soon et al. 2008). This is the current recommendation by most treatment guidelines.

1.4 Rationale for studying microbiome

The necessity for less harsh treatment alternatives and preventative measures against breast cancer has long been a growing area of interest and investigation. Screening for early detection is one of the primary standards of prevention [1], and while there is strong merit in the prevention of later stage diagnosis with regular screening, there are very few resources in place for individuals from low socioeconomic statuses (SES) who do not have access to reliable health care and routine examinations [2]. In the past few decades research has focused on the impact of modifiable lifestyle factors such as diet and exercise. There has been an increasing body of literature suggesting that nutritive compounds are capable of altering the epigenome, thereby reversing the malignant expression of genes responsible for cancer cell progression. Most recently, the microbiome has become an intriguing topic with regard to cancer treatment and prevention as a modifiable lifestyle factor that may be regulated through diet; however, the literature is severely lacking with regard to its epigenetic impact on breast cancer.

The Human Microbiome Project demonstrated that race/ethnicity was strongly associated with the microbiome in healthy individuals [3], and data examining a small group of colorectal cancer patients has shown that fecal microbiomes differ by racial/ethnic group [4]. Whether differences in the gut microbiome of black and white women contribute to the disparities observed in both incidence of the various breast cancer phenotypes, as well as mortality rates, remains unstudied. We have begun the process of collecting stool samples of breast cancer patients under our existing IRB approved bio-banking protocol, and once sufficient data is gathered we will begin generating additional hypotheses for testing in conjunction with the one following. Since there is compelling evidence that certain short chain fatty acids (SCFA) have the ability to regulate epigenetic genes in colorectal cancer, we suspect that they have inhibitory effects on aberrantly expressed genes in breast cancer as well. One of our eventual goals is to elucidate the mechanisms by which SCFA-producing microbes regulate epigenetic processes that regulate breast cancer progression.

We hope to determine how the incorporation of more leafy greens and fruits in the human diet impacts the gut microbiome and how that impact affects dysregulated epigenetic processes in cancer. In this project we seek to gather preliminary data and determine if SCFA are capable of modifying epigenetic genes known to aid in breast cancer cell growth in either Caucasian or African American cell lines. The data gathered from this project will provide additional rationales to study the gut microbiome in depth with regard to breast cancer prevention. We also intend to provide evidence in support of microbial transplantation in a phase II clinical trial. In addition, these data will provide additional explanations for the effectiveness of a healthy diet in the human organism. We hypothesize that SCFA are capable of promoting breast cancer cell death through epigenetic modulation of genes

responsible for breast cancer cell progression. We will perform a microbiome-wide association study (MWAS) to determine the microbial associations related to patient dietary patterns and SCFA profiles.

1.5 Neoadjuvant chemotherapy

The primary focus of neoadjuvant chemotherapy is to downsize tumors for mastectomy and therefore it is now the standard of care for selected patients in countries at all levels of resources (Lodge et al, 2011). Neoadjuvant chemotherapy can also be considered in high-risk patients such as young age or advanced disease where assessment of disease response to chemotherapy can be evaluated to determine mechanisms of resistance. Although neoadjuvant therapy does not appear to change relapse-free or overall survival rates it provides a means of *in vivo* chemo-sensitivity testing and diminishes the tumor size in most patients, which in turn increases the probability of successful breast conserving surgery in patients whose tumors are inoperable initially (Anderson et al, 2011). Randomized clinical trials of chemotherapy demonstrate similar long-term outcomes when patients are given the same treatment preoperatively compared to postoperatively (Rastogi et al. 2008).

Guidelines for the treatment of locally advanced breast cancer (LABC), with particular reference to resource-poor countries, have been published (El Saghir et al, 2011). The Breast Health Global Initiative (BHGI) summit in 2007 (http://portal.bhgi.org/Pages/Default.aspx) focused on the management and implementation of primary systemic therapy in the form of neoadjuvant chemotherapy in locally advanced breast cancer and two important points were highlighted (BHGI). Firstly, all trials demonstrating the efficacy of neoadjuvant chemotherapy in breast cancer have been conducted in developed countries. Secondly, there is a paucity of literature on the impact of neoadjuvant chemotherapy for LABC in developing countries such as Nigeria and, although scientific advances drive management guidelines, the implementation is limited by local resources and expertise.

Although newer agents are being studied in resource-rich nations, neoadjuvant chemotherapy for LABC remains predominantly anthracycline-based, particularly in resource-poor nations. In spite of high response rates to anthracycline-based regimes, only a small fraction of patients achieve pathologic complete response (pCR); more than 60% of patients with LABC continue to die of metastatic breast cancer; and disease-free survival (DFS) rates remain modest at only about 30% at 5 years (Arowolo et al., 2013). Clearly, further refinement of treatment strategies and introduction of more effective and less toxic chemotherapeutic agents are warranted. It is conceivable that a less toxic regimen given for longer duration might lead

to more complete pathologic responses and improved quality of life as has been demonstrated in patients with hormone receptor positive tumors treated with prolonged duration of hormonal therapy. The identification of effective agents that can be used alone or in combination with other agents is uniformly accepted as an appropriate drug development strategy in breast cancer.

In the first breast cancer clinical trial ever conducted in Nigeria, we tested single agent capecitabine in chemotherapy naïve patients with LABC. The study was designed to demonstrate feasibility of a phase II trial involving an oral chemotherapeutic agent such as capecitabine in a resource-poor nation like Nigeria. A total of 16 patients were recruited from August 2007 to April 2010. After the first 3 cycles of therapy, pCR were seen in 5 of 16 patients (31%; 95% CI 11% - 59%). Of the remaining 11 patients, 8 had no response (NR) or stable disease (SD), and 3 had progressive disease (PD). OCR at the end of 8 cycles was 44% (95% CI 20% - 70%). Clinical response and radiologic response by ultrasonomammography were highly concordant (Spearman correlation 0.70). The most common adverse effect was Grade 1 handfoot syndrome, which was seen in 75% of patients. Despite several limitations, we carried out this phase II feasibility study of neoadjuvant capecitabine for locally advanced breast cancer in Nigeria but the study terminated early due to poor accrual. Nearly 90% of patients were screen failures because they already had metastatic disease (Arowolo et al, 2013). As a nation, Nigeria will heavily invest in its health infrastructure including the needed infrastructure for effective cancer control for early detection and treatment of common cancers. This study is designed to test the feasibility of establishing a robust platform for multi-institutional biomarker driven oncology clinical trials as a way to improve quality of cancer care in Nigeria. Data from this clinical trial and subsequent phase III study should inform cancer control policies in Nigeria.

1.6 Introduction to investigational treatment(s) and other study treatment(s)

1.6.1 Docetaxel (TXT)

Docetaxel (Taxotere, TXT) is a semisynthetic member of the taxoid class of antineoplastic agents. It is effective in the treatment of patients with advanced (locally advanced or metastatic) breast cancer. Reported objective response rates for docetaxel 100 mg/m^2 ranged from 54% to 69% and 53% to 82% as first-line monotherapy or combination therapy, respectively. Objective response rates of 23% to 65% and 30% to 81% have been reported for docetaxel as second-line monotherapy or combination therapy (Figgit and Wiseman 2000).

The antitumor activity of docetaxel is based on binding to tubulin and stabilization of non-functional microtubule bundles, thereby blocking normal mitotic spindle development and subsequent cell division (Ringel, 1991). A dose of 100 mg/m^2 is below the critical threshold for nonlinear pharmacokinetics (about 250 mg/m^2). Docetaxel elimination is mediated by hepatic metabolism, followed by biliary excretion of its metabolites. Only a minor fraction is excreted unchanged in urine (< 15%). Of note, docetaxel is predominantly metabolized in the liver by cytochrome P450 (CYP) isoenzyme CYP3A4 and, to a lesser extent, by CYP3A5. The pharmacokinetics of docetaxel may also be altered *in vivo* as a result of interactions with compounds that are substrates, inducers, or inhibitors of CYP3A4, CYP3A5 and glycoprotein

(Baker et al 2009).The toxicity of docetaxel includes myelosuppression, arthralgia and myalgia. Neurotoxicity is a dose limiting toxicity and can manifest as peripheral neuropathy. These toxicities do not lead to stoppage of treatment, except if severe (Seidman AD et al, 1998).

1.6.2 5-Fluorouracil, Epirubicin, Cyclophosphamide (FEC)

The combination of 5-Fluorouracil, Epirubicin, Cyclophosphamide (FEC) is one of the chemotherapy regimens approved for the treatment of breast cancer. It is approved as an adjuvant treatment following treatment with taxanes. The NCCN guideline also approves FEC regimen in combination with trastuzumab and pertuzumab for patients with HER2-positive breast cancer in both neoadjuvant and adjuvant settings (NCCN for breast cancer 2016). A 21-day schedule (fluorouracil 500 mg/m^2, epirubicin 75 mg/m^2, and cyclophosphamide 500 mg/m^2) given for 3 doses is recommended following administration of a taxane with trastuzumab/pertuzumab (Buzdar et al, 2013: NCCN, 2016). The main toxicity associated with this regimen is neutropenia, fatigue and cardiotoxicity though not life threatening.

1.5.3. Herceptin (Trastuzumab) (H)

Trastuzumab (Herceptin), a humanized monoclonal antibody against the extracellular domain of HER2, has been shown to benefit patients with HER2 positive metastatic breast cancer when administered weekly or every three weeks, alone or in combination with chemotherapy. The pharmacokinetics of trastuzumab is characterized by low systemic clearance, low volume of distribution and a very long half-life (28 days). The elimination pathways have not yet been defined and the clinical relevance of trastuzumab kinetic variability is unknown. No drug-drug interactions have been reported. Dosage regimens of trastuzumab are similar in the adjuvant setting (postoperative) and in metastatic disease (Levêque et al, 2008). Trastuzumab is not associated with the adverse events that typically occur with chemotherapy (alopecia, myelosuppression, and severe nausea and vomiting) with the exception of hypersensitivity, which has been reported mainly and occasionally with the first infusion, furthermore cardiotoxicity (principally congestive heart failure) is the most important adverse effect of trastuzumab. Cardiotoxicity has been reported in 1.4% of women who received the drug as a single agent for metastatic disease. This adverse effect is higher if combined with anthracyclines than with taxanes (Piccart-Gebhart et al, 2005). Refer to the trastuzumab Package Insert for additional information.

The usual route of administration for trastuzumab is through intravenous infusion. However, there is the recently EU-approved subcutaneous formulation of trastuzumab - Herceptin SC (Hourcade-Potelleret et al, 2014; Roche, 2015), which will be tested in this study. The subcutaneous formulation of trastuzumab has been reported to have a pharmacokinetic profile and efficacy non-inferior to the intravenous administration, with a similar safety profile and has been reported to be preferred by patients due to its convenience (Ismael et al. 2012, Pivot et al. 2013). A fixed subcutaneous dose of 600mg given every three weeks for 18 cycles given concurently or sequentialy with chemotherapy has been reported to be safe and tolerable (Gligorov et al. 2017). In the aforementioned studies, indigenous black populations in Africa

were not included, yet they are the ones most likely to benefit from highly effective cancer medicines. This is a Phase II feasibility study to evaluate the efficacy and toxicity of four cycles every 3 weeks of docetaxel + trastuzumab (TH) in previously untreated women with non-metastatic breast cancer.

1.5.5 Tamoxifen (TAM), letrozole (LET) and Luteinizing Hormone Releasing Hormone (LHRH) agonist.

Tamoxifen is an anti-estrogen used for treatment of hormone positive breast cancer and the treatment of anovulatory infertility. It is an estrogen receptor antagonist (selective estrogen receptor modulator).

Letrozole is an aromatase inhibitor (AI) indicated in the first line treatment of hormone positive breast cancer in post-menopausal women. Both drugs have been FDA approved and are in use for the treatment of indicated conditions. Refer to drug information package insert for further information.

Luteinizing hormone-releasing hormone (LHRH) agonist is used for endocrine manipulation to medically suppress the function of the ovaries in premenopausal patients with advanced breast cancer. LHRH agonist has direct antitumor effects leading to decrease in the circulating estrogen concentrations to postmenopausal levels and is an alternative to surgical oophorectomy. Recent findings by two randomized, phase 3 trials, the tamoxifen and Exemestane Trial (TEXT) and the Suppression of Ovarian Function Trial (SOFT), involving premenopausal women with hormone-receptor–positive breast cancer, showed that ovarian suppression by oophorectomy (surgical or radiation) or LHRH agonist plus TAM or AI is considered as the new effective standard treatment (Francis et al, 2015; Pagani et al, 2014).

Recent studies including Gynecologic Oncology Group Protocol-0199 (GOG-0199) results indicate that risk-reducing salpingo-oophorectomy (RRSO) lowers mortality from ovarian/tubal and breast cancers not only among *BRCA1/2* mutation carriers but has potential benefits among high-risk noncarriers. In addition, women who had a bilateral oophorectomy and who used hormonal therapy had nearly a threefold lower risk of incident coronary heart disease than women who had not used hormonal therapy after oophorectomy or those who never used hormonal therapy (Francis et al, 2015; Sherman et al, 2014). Our longterm goal is to optimize therapy for the predominantly young women who develop aggressive breast cancer in Nigeria and develop national guidelines for optimal management of these patients.

2 Rationale

2.1 Study rationale and purpose

Racial/ethnic disparities in breast cancer mortality continue to widen and biomarker intensive clinical trials rarely interrogate breast cancer in underserved populations of African ancestry. Through genome, exome, and RNA sequencing, we recently examined the molecular features of breast cancers using 194 patients from Nigeria and 1,037 patients from The Cancer Genome Atlas (TCGA). Relative to Black and White cohorts in TCGA, we saw substantial

genomic differences and Nigerian tumors are characterized by increased homologous recombination deficiency signature, pervasive *TP53* mutations and greater structural variation — indicating aggressive biology. This dataset provided novel insights into potential molecular mechanisms underlying outcomes disparities and lays a foundation for deployment of precision therapeutics in underserved populations (Pitt et al 2018).

Nigeria is long overdue for implementation of this clinical trial as there is no standard of care for HER2 targeted therapy in Nigeria. In collaboration with Roche Global and the Investigative team of Nigerian Oncologists, the study will determine the efficacy and toxicity of 4 cycles of three-weekly neo-adjuvant docetaxel chemotherapy in conjunction with Herceptin SC followed by one year of Herceptin SC in Nigerian women with HER2-positive breast cancer. Of note, no one can afford HER2 targeted therapy in Nigeria or even standard chemotherapy without access to health insurance.

The study considers three weekly docetaxel as recommended backbone to be combined with Herceptin SC with manageable toxicity rate. This combination has been shown to be efficacious with manageable toxicities in other populations. One of the objectives for this biomarker intensive phase II feasibility study is to establish a platform for clinical trials in Nigeria and identify genomic biomarkers of response and toxicity to protocol driven and optimal HER2 targeted therapy in Nigerian women. Nigerian patients receive sub-optimal therapy even when they can afford the treatments. Three-weekly taxane has been reported to be well tolerated by elderly patients with breast cancer and one of our objectives is to use this clinical trial to improve management of chemotherapy toxicities by training treating oncologists and nurses about optimal care of patients undergoing treatment for cancer (Beuselinck et al., 2010).

Without optimal chemotherapy, breast cancer mortality in the Nigerian population will remain high. Ensuring the development of cancer research that meets the needs of people who live in low resource settings is integral to alleviating the cancer burden. There is a view that a focus on phase II and III cancer clinical trials in sub-Saharan Africa can give African researchers the opportunity to "learn by doing", to foster sustainable clinical research capacity through international collaboration between African partners, the developed countries and regional collaboration between African research institutes (RAND 2015). Research collaborations between African and non-African partners hold the potential to have a significant impact on the development of clinical research capacity in sub-Saharan Africa. This study is a collaboration between the University of Chicago and multiple centers in Nigeria.

Among the study population, there are no data on the response rate, toxicity and quality of life during treatment with any cancer therapy and none for docetaxel or HER2 targeted therapies. Docetaxel can be used in elderly patients and is well tolerated in patients with mild cardiac pathology in which the commonly used doxorubicin-based combination may be contraindicated. Docetaxel has been successfully combined with Herceptin IV (trastuzumab) in patients with HER2-positive breast cancer without potentiating the cardiotoxic effect of

Herceptin (Burris III, 2004). In this study, FEC will be given to patients with operable stable disease or partial response (in an attempt to induce pCR), as well as those with progressive disease or inoperable stable disease after four cycles of TscH. However, patients with pCR after TH can be spared the cardiotoxicity associated with doxorubicin-based chemotherapy. With this schedule, we believe we can identify a subset of patients with excellent response to TscH who will then complete one year of Herceptin SC. Patients will be evaluated for toxicity and clinical improvements before each cycle, but a breast ultrasound (USS) to assess and document response using RECIST criteria will be performed every 6 weeks during Taxetere + Herceptin SC, and every 6 weeks during FEC and at 4th week after last dose of FEC for those indicated.

2.2 Rationale for choice of drug and schedule

2.2.1 Docetaxel (TXT) and Trastuzumab

There is no standard of care for Nigerian patients with HER2 positive breast cancer because the Nigerian Health System has not been able to provide access to essential cancer medicines for the population. While HER2 blockade is standard of care in developed countries, this study will aim to establish the efficacy of Docetaxel in combination with HER2 targeted therapy in Nigerian patients.

Docetaxel is an acceptable alternative regimen for patients with poor response to CMF (Cyclophosphamide, Methotrexate, 5-Fluorouracil) and Adriamycin and Cyclophosphamide (AC) chemotherapy. In a randomized open-label phase III study to compare the pharmacokinetics, efficacy and safety of subcutaneous (SC) Herceptin (trastuzumab) with intravenous (IV) Herceptin (trastuzumab) administered in women with HER2-positive early breast cancer, the study demonstrated non-inferiority of pharmacokinetics and efficacy for Herceptin SC *versus* Herceptin IV (Ismail et al., 2012). Overall, the safety profiles of the Herceptin SC and IV formulations were reported as comparable and consistent with the known safety profile of Herceptin. Therefore, Herceptin 600 mg three-weekly dose administered subcutaneously in approximately 5 minutes provides a valid treatment alternative to the three-weekly intravenous Herceptin regimen (Roche 2012). Subcutaneous Herceptin is in routine use in Europe and marketed in Nigeria, and therefore is a more convenient way to administer the drug. In an international, randomised PrefHer study to assess patients' preference for either subcutaneous or intravenous trastuzumab, patient preference and safety results from PrefHer, combined with the known non-inferior efficacy and pharmacokinetic and safety profile data, suggest that a fixed dose of 600 mg trastuzumab administered subcutaneously every 3 weeks is a validated, well tolerated treatment option for HER2-positive breast cancer, and is the preferred treatment by patients (Pivot et al., 2013).

Dual HER2 blockade has been tested to improve overall outcomes in HER2 postive breast cancer but this has not been accepted as standard of care in every country as the results are mixed. The added toxicity of dual blockade with potential added financial toxicity makes it hard to justify dual blockade as the first clinical trial to be conducted in a low resource setting

like Nigeria. Furthermore, there is new evidence that patients who achieve pCR after THP can be spared the added toxicities associated with doxorubicin based chemotherapy. Hence, this study will consider a backbone of Taxotere chemotherapy X 4 cycles + Herceptin SC in the neoadjuvant setting to identify the genomic biomarkers of response that would be carried to a phase III study to determine the most efficient and cost effective combination neoadjuvant treatment for the majority of HER2 positive patients in Nigeria, where there is no population based screening and the majority of patients present with clinically palpable masses.

In summary, this phase II feasibility study will test the efficacy of Taxetere + Herceptin SC X 4 cycles in the neoadjuvant setting and one year of Herceptin SC as adjuvant therapy for HER2 positive breast cancer among Nigerian women. For responsders who do not have complete clinical response after 4 cyclers of Taxetere, they will receive an additional 3 cycles of neoadjuvant non-cross resistant FEC + Herceptin SC. This study will be used to test an emerging platform for future biomarker based multi institutional oncology clinical trials in Nigeria and across Sub-Saharan Africa.

3. Objectives and endpoints

Objectives and related endpoints are described in Table 3-1 below.

Table 3-1 Objectives and related endpoints

Objectives	Endpoints and Analyses	Analysis
**Primary and key secondary**		Refer to Section 9.4
To determine the efficacy of docetaxel + Herceptin SC every three weeks for four cycles with or without FEC + Herceptin SC for three cycles (with Tamoxifen/Letrozole added to ER/PR Positive patients) in Nigerian women with HER2-positive breast cancer<br><br>To determine the toxicity of every three-weeks docetaxel + SC Herceptin with or without FEC + Herceptin SC for three cycles (with Tamoxifen/Letrozole added to ER/PR Positive patients) in Nigerian women with HER2-positive breast cancer	1. Percentage of participants achieving pathological complete response (pCR) at surgery is the primary end point. [Time Frame: Approximately 4 – 6 months from commencement of chemotherapy. (Surgery will be performed within 2 weeks after Cycle 4 of T+scH for those with complete clinical response or after additional 3 cycles of FEC for those with stable disease or partial clinical response to T+scH.)]<br>2. Percentage of participants achieving pCR by hormone receptor status [Time Frame: Approximately 4 – 6 months from commencement of chemotherapy. (Surgery will be performed within 2 weeks after Cycle 4 of T+scH for those with complete clinical response or after additional 3 cycles of FEC for those with stable disease or partial clinical response to T+scH.)]<br>3. Percentage of participants achieving pCR by lymph node status [Time Frame: Approximately 4 – 6 months from commencement of chemotherapy. (Surgery will be performed within 2 weeks after Cycle 4 of T+scH for those with complete clinical response or after additional 3 cycles of FEC for those with stable disease or partial clinical response to T+scH.)]<br>4. Percentage of participants achieving pCR by breast cancer subtype [Time Frame: Approximately 4 – 6 months from commencement of chemotherapy. (Surgery will be performed within 2 weeks after Cycle 4 of T+scH for those with complete clinical response or after additional 3 cycles of FEC for those with	

Objectives	Endpoints and Analyses	Analysis
	stable disease or partial clinical response to T+scH.)]<br>Note: Pathological complete response in the breast is defined as the absence of invasive cells at microscopic examination of the primary tumor and lymph nodes at surgery. Any remaining in-situ lesions are permissible. Participants with invalid/missing pCR assessments will be defined as non-responders.<br>5. Percentage of participants experiencing grades 3 and 4 haematological, gastro-intestinal, neurological and cardiovascular toxicities. Time Frame: at any point during serial assessment from commencement of chemotherapy and Herceptin SC till 10 years or withdrawal or death.<br>Clinical and laboratory adverse events (AEs) will be reported according to the National Cancer Institute-Common Terminology Criteria for Adverse Events (NCI-CTCAE) version 4.0. In addition, cardiovascular side effects will be reported according to prespecified criteria (NCI-CTCAE and New York Heart Association [NYHA]).<br>Left ventricular ejection fraction (LVEF) will be assessed using echocardiogram (ECHO) scans. Cardiac reassessment throughout the study will be performed using the same technique as at baseline.	
**Secondary**		Refer to Section 9.5.2
To evaluate other response-related endpoints: duration of response (DOR), clinical response, progressive disease during neoadjuvant treatment, breast-conserving surgery, invasive	1. Percentage of participants achieving clinical response (CR and PR) during neo-adjuvant period by breast ultrasound [Time Frame: Approximately 4 – 6 months from commencement of chemotherapy. (Surgery will be performed	

Objectives	Endpoints and Analyses	Analysis
disease-free survival	within 2 weeks after Cycle 4 of T+scH for those with complete clinical response or after additional 3 cycles of FEC for those with stable disease or partial clinical response to T+scH.)]<br>2. Percentage of pParticipants with progressive disease during neo-adjuvant treatment period [Time Frame: Baseline up to Cycle 4 (assessed at Baseline, Day 1 of Cycles 1-4 Pre-Surgery)<br>3. NB: Tumor assessments based upon the Response Evaluation Criteria in Solid Tumors (RECIST) criteria - version 1.0. The clinical response at each cycle up to the last assessment prior to surgery will be derived for: i) the primary breast lesion; (ii) across secondary breast lesions, (iii) across all breast lesions (iv) across axillary nodes (v) across supraclavicular nodes and (vi) across all nodes (vii) across all lesions (overall) using the following algorithm: CR: if measurement of '0' is noted at a given cycle as compared to baseline measurement which is >0 at screening; PR: if measurement is at least 30% decreased compared to baseline levels. (Reference= baseline size or sum of sizes). Clinical responders are participants who have achieved CR or PR during the neo-adjuvant treatment. Primary breast tumor clinical response is based on primary breast tumor assessment. Overall response is derived based on the sum total of breast tumors and all nodes examined. PD: if lesion is at least a 20 % increased from measurements at baseline. Percentage of participants achieving breast conserving surgery for whom mastectomy was planned [Time Frame: Approximately 4 – 6 months from commencement of chemotherapy. (Surgery will be performed within 2 weeks after Cycle 4 of T+scH for those with complete clinical response or after additional 3 cycles of FEC	

**Objectives**	**Endpoints and Analyses**	**Analysis**
	for those with stable disease or partial clinical response to T+scH.)]Breast Conserving Surgery (BCS) defined as quadrantectomy, lumpectomy, no surgery, axillary surgical resection or other method of avoiding mastectomy.<br><br>4. Invasive Disease Free Survival (iDFS) [Time Frame: from first dose of study drug treatment up to a maximum of 10 years. iDFS defined as the time from enrollment to the first documentation of recurrence of ipsilateral invasive breast tumor, recurrence of ipsilateral locoregional invasive disease, distant recurrence, contralateral invasive breast cancer or death due to any cause, as assessed by investigators. iDFS will be determined using Kaplan-Meier estimates.<br>5. DOR, defined as time from first response to disease progression or death.	
To determine the cardiac toxicity associated with TscH with or without FEC +scH in breast cancer patients<br><br>To determine the pharmacokinetic profile of Herceptin SC given in combination with Docetaxel	The percentage of participants with Heart failure (NYHA Class III or IV or as confirmed by a cardiologist) and a decrease in LVEF of at least 10 EF points from baseline and to below 50%.<br><br>Blood concentrations of Herceptin SC at multiple time points using the peak exposure C_{max} and the extent of exposure AUC $_{0\text{-}21d}$ from day 0 to day 21 will be determined.	

**Objectives**	**Endpoints and Analyses**	**Analysis**
To determine the pharmacokinetic profile of Herceptin SC given in combination with FEC following poor response to TH	Time-to-peak drug plasma concentration T_{max}, drug concentration at the end of the dosage interval C_{trough}	
To evaluate the quality of life (QoL) of patients on every three-weeks docetaxel (± FEC) with Herceptin over time	The various domains of QoL over time and the changes from baseline using the validated (by the European Organization for Research and Treatment of Cancer (EORTC)) QoL instrument (global and breast module). Analysis will be conducted to investigate the changes in HRQLover time.	
To explore mechanisms of resistance to HER2 targeted therapy in Nigerian women.	Genomic analysis of serial blood/serum specimens and evaluation of tumor biopsy specimens for genomic biomarkers of primary resistance to chemotherapy. Tumor subtypes/tumor biology will be correlated with patient's characteristics, response to treatment and iDFS	

4. Study design

4.1 Overview

This is a one-stage phase II study with a single arm design. It will be conducted in women with HER2-positive breast cancer with stages IIA to IIIC (defined by AJCC 2009 classification) of all hormonal statuses. All patients will receive 4 cycles of docetaxel + Herceptin SC. Breast ultrasound will be done after every 2 cycles (every 6 weeks) during neoadjuvant chemotherapy. Those with complete clinical response following breast ultrasound assessment after 4 cycles of docetaxel + Herceptin SC will undergo surgery. At surgery, those with pCR will not have further chemotherapy and they will receive one year of Herceptin SC to complete 18 cycles. Those with partial response or stable disease (operable) will be switched to have FEC+ Herceptin SC for additional 3 cycles in the neoadjuvant setting before surgery and continue to complete 18 cycles of Herceptin SC after surgery within one year. Patients with stable disease (inoperable) after salvage FEC + Herceptin SC or progressing patients before salvage FEC + Herceptin SC will go off study treatment and be followed for survival end point. Adjuvant treatment will include hormonal therapy for hormone receptor positive disease. Radiation therapy will be administered per standard of care.

The primary Endpoint is pCR in the one-stage phase II design. All patients will have active follow up every three months and have routine blood tests and physical examination for the first two years and every six months for 10 years. CT scan and bone scan will be done as clinically indicated to evaluate recurrence. (Figures 4-1, 4-2)

The response evaluation will be adapted from RECIST criteria (Response Evaluation Criteria In Solid Tumors, version 1.1) using breast ultrasound as follows:

1. Complete (Clinical) Response (CR): disappearance of breast lesion and lymph node, confirmed (after 4 cycles of TH or after 3 cycles of FEC + H). (Surgery should take place approximately 4 weeks after last dose of chemotherapy.)
2. Partial Response (PR): ≥30% decrease in the longest diameters of target breast /lymph node lesions, with the baseline measurements taken as the reference, confirmed at 16th week.
3. Partial Response inoperable (PRi): Disease that meets criteria for partial response following ultrasound assessment after 4 courses of TH and 3 additional courses of FEC + H but still surgically assessed as inoperable based on residual tumour size.
4. Partial Response operable (PRo): Disease that meets criteria for PR and is surgically assessed as operable based on residual tumour size.
5. Stable Disease (SD): neither PR nor PD criteria are met (may be operable [SDo] or inoperable [SDi]).
6. Progressive Disease (PD) ≥ 20% increase in the longest diameters of target lesions, with the longest diameters recorded when treatment started taken as the

reference, appearance of new lesions, or unequivocal progression of non-target lesions.

7. Pathologic Complete Response (pCR): If histo-pathological examination of surgical specimen (breast and lymph nodes) does not describe any gross or microscopic disease. Presence of insitu disease is permissible.

Each patient will be assigned one of the following response categories: 1) complete pathologic response, 2) partial response, 3) stable disease, 4) progressive disease, 5) early death from malignant disease, 6) early death from toxicity, 7) early death because of other cause, or 8) unknown (not assessable, insufficient data). Note: categories 2-4 are ultrasound findings for patients who do not have pCR or were deemed inoperable. Patients with a Complete Clinical Response who are found not to have a pCR will be categorized as partial responders.

All premenopausal patients will receive LHRH agonist for two years for contraception and fertility preservation. Hormone-receptor positive patients will receive hormonal therapy with tamoxifen or letrozole after surgery, radiotherapy and LHRH agonist according to the expression of hormone receptors ER and PgR (see glossary and section 10.3) and according to the state of primary menopause (see glossary) at the onset of the study.

Figure 4-1. Study Schema for Neoadjuvant Treatment

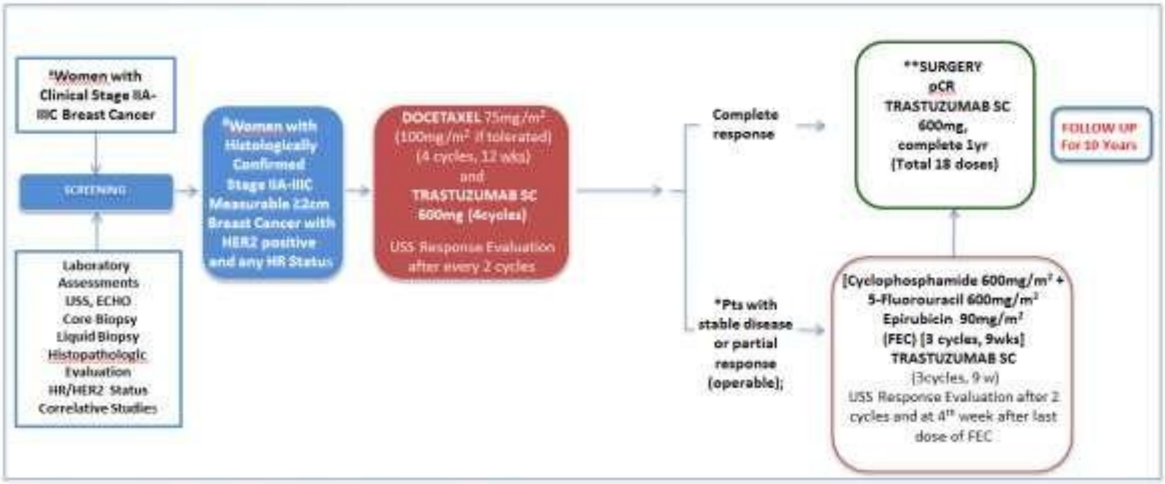

[a] Women must also meet other inclusion criteria listed in Sections 5.1 & 5.2

USS, Ultrasound Scanning; **ECHO**, Echocardiography

DOCETAXEL (T): total 4 three-weekly cycles to be administered with Trastuzumab

Cyclophosphamide, 5-Fluorouracil, Epirubicin (FEC): total 3 three-weekly cycles to be administered for partial responders or those with stable disease before surgery (see Section 6.2)

TRASTUZUMAB (Herceptin): total 4 three-weekly cycles with Docetaxel (or 7 doses for patients receiving FEC) preoperatively. * Inoperable patients and patients with progressive disease will be taken off study. **Patients with residual disease at surgery may receive additional chemotherapy at the discretion of treating oncologists if hormone receptor negative. Hormone receptor positive patients will receive hormonal therapy. After surgery, all patients will continue trastuzumab to complete 1 year of treatment

LHRH agonist GOSERILIN (Zoladex): 3.4 mg or 10.8mg SC for premenopausal patients at the onset of study treatment and every 3 months after chemotherapy for 2 years

Figure 4-2. Phase II Feasibility One- Stage Study Design

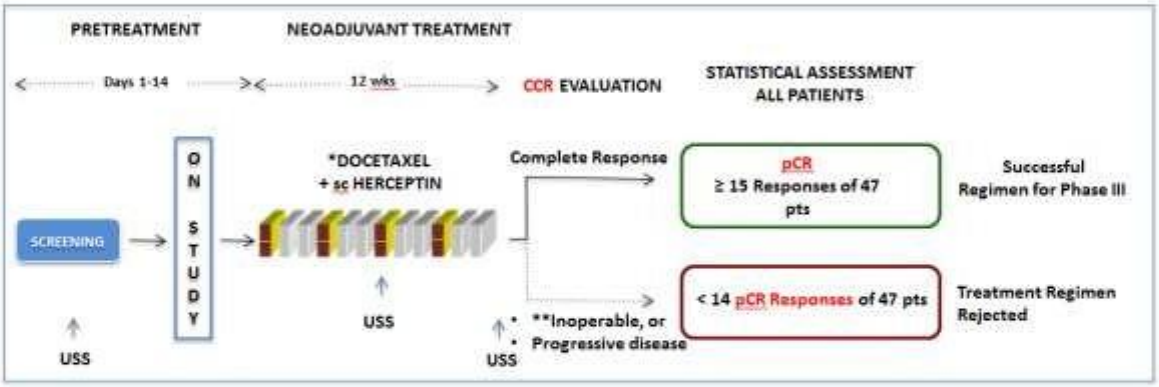

USS, Breast Ultrasound Scanning for Baseline and for Clinical Response Evaluation to be performed at **SCREENING**, after first 6 weeks of **TREATMENT** (2 cycles **DOCETAXEL and TRASTUZUMAB**) and after 12 weeks of TREATMENT (4 cycles **DOCETAXEL and TRASTUZUMAB**)

* Patients with breast cancer in Nigeria usually present with large tumors because there is no population screening. Partial responders or those with stable disease may need additional 3 Cycles of FEC before surgery and will be included in primary endpoint if they achieve pathCR

**Patients with progressive disease, inoperable or non-responders after 4 cycles of Docetaxel will go off study

pCR, pathological Complete Response

4.2 Definition of end of study

End of study Last Patient Last Visit (LPLV) will be upon completion of 10 years of follow up of last patient recruited into the study and collection of survival information.

Follow up visits will be carried out for all patients every 3 months for two years and thereafter every 6 months for the next 3 years and yearly for last 5 years (10 years total). During this period, patients will be monitored for duration of response/relapse (locoregional and distant) using clinical examination and blood tests as needed.

The first study report – Core Clinical Study Report (CCSR) will be done after all enrolled patients have either completed surgery or been deemed inoperable. Second report (Interim Clinical Study Report (ICSR) will be done after 5 years of follow up while the third report (Final Clinical Study Report, FCSR) will be done after 10 years of follow up.

4.3 Early study termination

The study can be terminated at any time for any reason by the investigators. Should this be necessary, study participants will be seen as soon as possible and the same assessments as for prematurely withdrawn patients will be conducted. The investigators will inform appropriate IRBs of the early termination of the trial.

5. Patient population

This study will be carried out among indigenous black women of all tribal groups in Nigeria. The investigator or designee must ensure that only patients who meet all the following inclusion and none of the exclusion criteria are enrolled and receive treatment in the study.

5.1 Inclusion criteria

Patients eligible for inclusion in this study should meet **all** of the following criteria:

Written informed consent must be obtained prior to any screening procedures

1. Women ages of 18 to 70 years old
2. Biopsy-accessible breast tumor of significant size for core needle biopsy/ultrasound measurable (≥ 2cm)
3. Patients with histologically confirmed carcinoma of the female breast with 3+ positive HER2 status by IHC
4. Clinical stages IIA –IIIC (AJCC 2009)
5. Chemotherapy-naïve patients (for this malignancy)
6. Performance status: ECOG performance status 0−1 (Appendix A)
7. Non-pregnant and not nursing. Women of childbearing potential must take the pregnancy test and must commit to receive LHRH agonist Zoladex (goserelin) for two years starting from the commencement of the study medications
8. Required Initial Laboratory Data. Adequate hematologic, renal and hepatic function, as defined by each of the following:
    1. Granulocyte ≥ 1,500/µL
    2. Platelet count ≥ 100,000/µL
    3. Absolute neutrophil count (ANC) ≥ 1500/µL
    4. Hemoglobin ≥ 10g/dL
    5. Bilirubin ≤ 1.5 x upper limit of normal
    6. SGOT and SGPT < 2.5 x upper limit of normal
    7. Creatinine within institutional normal limits or glomerular filtration rate ≥ 30 mL/min/1.73 m^2 by CKD EPI equation (see http://mdrd.com/ for calculator)
9. ECHO: Baseline left ventricular ejection fraction of ≥ 55%

5.2 Exclusion criteria

Patients eligible for this study must not meet **any** of the following criteria:

1. Pregnant or lactating women. Women of childbearing potential not using a reliable and appropriate contraceptive method. Postmenopausal women must have been amenorrheic for at least 12 months to be considered of non-childbearing potential. Patients of childbearing potential will agree to continue the use of

acceptable form of contraception for 24 months from the date of last Herceptin administration.

2. Patients with distant metastasis (brain and/or visceral metastasis)
3. Serious, uncontrolled, concurrent infection(s).
4. Treatment for other carcinomas within the last 5 years, except non-melanoma skin cancer and treated cervical carcinoma *in-situ* (CCIS)
5. Participation in any investigational drug study within 4 weeks preceding the start of study treatment
6. Other serious uncontrolled medical conditions that the investigator feels might compromise study participation including but not limited to chronic or active infection, HIV-positive patient, uncontrolled hypertension, symptomatic congestive heart failure, unstable angina pectoris, uncontrolled Diabetes mellitus, or psychiatric illness/social situations that would limit compliance with study requirements.
7. Patients with HER2-negative disease

5.3 Recruitment process

As the first investigator initiated biomarker based clinical trial in Nigeria, we have spent the past year developing a robust recruitment plan as detailed below:

1. Patient base: We have established a screening log at each site. In the past 6 months, we have refined our screening process and 35 potential patients were identified. Enrollment will not be competitive at each site because there is no ongoing study at any of the sites in Nigeria.

2. Extensive outreach efforts: We are also planning extensive outreach efforts to include
a) Media coverage at protocols launch. We will partner with the Nigerian Federal Ministry of Health, Committee of Chief Medical Directors, Breast Cancer Advocacy Groups and Community members to develop publicity around the launch so that Nigerian Citizens are aware of the availability of the Clinical trial in four sites in Nigeria. The trial will also be posted at the websites of all the participating institutions.
b) Information on availability of the trial will be sent to colleagues at nearby institutions using emails, text messages, and relevant professional group social media to direct eligible participants for the study.
c) Flyers and pamphlets containing information on the trial will be prepared and distributed to clinicians and health workers at meetings to provide information on the trial. The flyers will also be distributed to the public (sample of advert –appendix H) .
d) The trial will also be advertised on mass media such as radio and television and newspapers.
3. We have established multidisciplinary breast cancer Management Tumor Board at UCH Ibadan with plans to establish tumors Boards at the other collaborating Institutions where

eligible patients will be screened. At LUTH there is a breast cancer clinic where all the clinicians see patients jointly. Recruitment will be facilitated at this center.

4. Lastly, within our Translational research program, we are developing a robust recruitment and retention program to remove barriers to participation in clinical trials among Nigeria breast cancer patients.

6. Treatment

6.1 Study treatment

For this study, "Study treatment" refers to all combinations of antineoplastic drugs given during the course of the trial. All dosages prescribed and dispensed to the patient and all dose changes during the study must be recorded.

The investigator or responsible site personnel will instruct the patient to take the study drugs as per protocol (promote compliance). Drug accountability must be performed on a regular basis. The site personnel will ensure that the appropriate dose of each study drug (chemotherapy and anti HER 2 agents) is administered at each visit and will provide the patient with the correct amount of any oral medications.

We plan to treat patients with docetaxel + Herceptin SC three-weekly for a total of 12 weeks. Ultrasonography for response assessment will be done every 6 weeks and patients achieving complete clinical response after 4 cycles of docetaxel + Herceptin SC will undergo surgery. Each study site has a team of surgeons included in the study team. These surgeons will be participating in all aspects of the surgery related to the study protocol. Partial responders or patients with stable disease will be given 3 additional cycles of non-cross resistant FEC + Herceptin SC. If there is evidence of disease progression and inoperable stable disease, patients will be taken off study treatment.

1. The HER2 status will be detected in core biopsy for initial assignment and in resected tumors to re-assess HER2 status for treatment evaluation. Cases with HER2 IHC score 3+ and cases with score 2+ that are confirmed by FISH or CISH for gene amplification will be considered HER2+.
2. Patients will undergo surgery within 4- 6 weeks of last dose of chemotherapy regimen. Surgical tumor specimen will undergo routine institutional histo-pathological examinations. It will be used for confirmation of complete response in this study, i.e. whether clinical complete response corresponds to pathological complete response. ER/PR and HER2 statuses will be re-evaluated on any residual tumor. Residual tumor samples will be sent for genomic analysis to evaluate molecular markers of response..
3. Radiation therapy will be given after chemotherapy and surgery according to institutional practice. As a general guideline, radiation treatment will be started 6-8 weeks after completion of definitive surgery and chemotherapy. Patients undergoing breast conservation therapy will be treated to the intact breast with tangential fields at 180-200

cGy/fraction to a total dose of 5000-6600 cGy, including any boost. It is recognized that use of post mastectomy and regional nodal radiation is variable. Recommended guidelines are to treat the post-mastectomy chest wall and/or nodal regions in patients with 4 or more positive nodes in the axilla and in patients with tumor of 5 cm or more in size to 4500-5500cGy at 180-200 cGy/fraction. As there is no radiation question in this protocol, there will be no dosimetry or portal field review.

4. Adjuvant hormone therapy is recommended for 5-10 years for all ER+ or PgR+ patients after the completion of surgery and chemotherapy (ER+/PgR+ defined as ≥1% of stained nuclei/hpf). Hormone receptors status will be determined in initial core biopsy for assignment of targeted therapy with TAM or Aromatase Inhibitor of choice and it will be re-assessed in resected tumors.
5. Tamoxifen (20 mg orally once daily) or an aromatase inhibitor letrozole 2.5mg orally once daily (for documented postmenopausal women only) will be started within 12 weeks of completion of chemotherapy. This allows for the variable period needed for surgery to be completed. All premenopausal patients will receive LHRH agonist Zoladex (goserelin) 10.8mg (subcute) every 3 months for 2 years from the onset of study treatment.
6. Quality of life (QoL) assessment using EORTC QoL instrument (English or Yoruba language versions) will be administered for all participants before each chemotherapy session (Appendices B-D).

6.2 Dosing guidelines

Docetaxel will be given at 75 mg/m^2, increasing to 100 mg/m^2 from cycle 2 onwards if the 75mg starting dose was well tolerated (by i.v. infusion and diluted in NS or D5W) and given every three weeks for four cycles. Patients with CCR will have surgery within 4 weeks of last dose of docetaxel + Herceptin. Patients with partial response or stable disease will continue with 3 doses of FEC (three cycles of intravenous FEC (fluorouracil 600 mg/m^2, epirubicin 90 mg/m^2, and cyclophosphamide 600 mg/m^2- diluted in NS or D5W) + Herceptin every 3 weeks before surgery.

All patients will receive anti-HER-2 blockade Herceptin. Herceptin therapy at 600 mg subcute (fixed dose irrespective of patients' weight /body surface area) on day 1 of each cycle and thereafter every three weeks. A total of 18 doses will be given (one year). Herceptin dosing is not interrupted during surgery.

The injection site should be alternated between the left and right thigh and should be administered at a steady rate over 3 - 5 minutes using a hand-held syringe. New injections should be given at least 1 inch/2.5 cm from the old site in healthy skin and never into areas where the skin is red, bruised, tender, or hard. During the treatment course with Herceptin SC, other medications for SC administration should preferably be injected at different sites.

For all patients docetaxel will be administered after 30-60 minutes of trastuzumab administration.

Oral tamoxifen 20mg or Arimidex Img or letrozole 2.5mg or Exemesatane 25 my (with Zoladex as indicated) will be given daily to complete 5-10 years of adjuvant hormonal treatment. All premenopausal patients will receive SC LHRH agonist goseriline (Zoladex) 10.8mg on day 1 of chemotherapy and every 3 months after chemotherapy for 2 years.

6.3 Ultrasound evaluation of response

Ultrasonography of the breast for evaluation of response shall be done at baseline and every 6 weeks during neoadjuvant treatment by study radiologists at each site. Radiologist will report the results from ultrasonography as part of routine clinical care. Central review will be conducted by the Radiology Subcommitee of the clinical trial group.

6.4 Dosing regimen

Table 6-1 Dose and treatment schedule

Study treatment	Pharmaceutical form and route of administration	Dose	Frequency and/or Regimen
Docetaxel	Intravenous infusion (1 hr)	75 mg/m^2, then 100 mg/m^2 from cycle 2 onwards if tolerated	Day 1, 21 ---- (3-weekly) 4 doses
Epirubicin	Intravenous infusion (1 hr)	90mg/m^2	3-weekly cycle, 3 doses
5 Fluorouracil	Intravenous	600mg/m^2	3-weekly cycle, 3 doses
Cyclophosphamide	Intravenous	600mg/m2	3-weekly cycle, 3 doses
Trastuzumab (Herceptin)	Subcutaneous injection (2-5 mins)	600mg	Day 1 (21 day cycle) 18 doses
*Tamoxifen	Oral tablets	20 mg	Daily (continuous) after surgery for 10 years
*Letrozole	Oral tablets	2.5 mg	
*LHRH agonist goserelin (Zoladex)	Subcutaneous injection	10.8mg	Every 3 months for 2 years

*Tamoxifen/Letrozole/LHRH agonist only for HR+ pts; LHRH agonist goserelin (Zoladex) only for pre-menopausal pts

6.5 Treatment duration

The planned treatment duration for study and supportive drugs are as follows:

1. Docetaxel 4 cycles with Herceptin SC (12 weeks) as neoadjuvant therapy before surgery. Docetaxel should be administered after trastuzumab. An observation period of 60 minutes is recommended after the first dose of trastuzumab injection, thereafter an observation period of 30-60 minutes after subsequent injections before docetaxel.
2. FEC: 3 cycles + scH of 21-day cycles (9 weeks) for partial responders or stable disease patients to TscH starting after assessment of reponse at week 16. (This allows 3 weeks for the effect of the last dose of TscH to manifest before response evaluation).
3. Trastuzumab (Herceptin SC): 18 cycles every three weeks (52 weeks) for each patient starting at the first day of treatment with docetaxel. The injection site should be alternated between the left and right thigh.
4. Missed doses: If a dose of Herceptin SC is missed, it is recommended to administer the next 600 mg dose as soon as possible. The interval between subsequent Herceptin SC injections should not be less than three weeks. If the dose of docetaxel or FEC is missed, next dose can be administered within two weeks and patient will continue in the study. In cases of missed interval of more than two weeks, patients can receive next dose but will not be included in further response evaluation analysis.
5. Tamoxifen: 20mg daily for 10 years after surgery and chemotherapy/LHRH or Letrozole: 2.5 mg daily for 10 years after surgery/chemotherapy as an alternative to tamoxifen
6. LHRH agonist (goserelin) 3.4 mg or 10.8mg every 3 months for 2 years starting from the commencement of the study medications

6.6 Pretreatment considerations:

Docetaxel

1. Hydration: Patients should maintain adequate oral hydration (2 to 3 L/day)
2. Oral dexamethazone 8mg twice daily for 3 days starting 1 day prior to docetaxel administration
3. i.v. ondansetron 8mg, i.v. dexamethaxone 20mg, i.v. diphenhydramine 50mg, i.v. ranitidine 50mg

Infection prophylaxis: primary prophylaxis for GCSF is indicated for all patients using Docetaxel . Filgrastim should be given at least 24 hours after docetaxel

FEC

1. Hydration: Patients should maintain adequate oral hydration (2 to 3 L/day)
2. Hydration: Patients should maintain adequate oral hydration (2 to 3 L/day)
3. i.v. ondansetron 8mg, i.v. dexamethaxone 20mg, i.v. diphenhydramine 50mg, iv ranitidine 50mg.
4. Infection prophylaxis: Primary prophylaxis for GCSF is not indicated.

Trastuzumab

1. i.v. diphenhydramine 50mg with i.v. dexamethasone 8mg bolus (after docetaxel and FEC chemotherapy since diphenhydramine with dexamethasone will be part of premedication for docetaxel, and dexamethasone will be part of FEC chemotherapy)

6.7 Guidelines for continuation of treatment

All enrolled and responding patients will be treated with TH regimen for a total of 12 weeks before surgery. If there is evidence of stable disease (SD) or partial response after 12 weeks, such patients will be placed on FEC regimen for 3 cycles with Herceptin before reassessment. Patients showing clear progression at 12 weeks will be taken off study treatment and managed per treating physician. Among those receiving FEC, if after completion of FEC the patient has progressed or is deemed inoperable, they will be taken off study treatment and managed per treating physician.

6.8 Dose modification and dose delay

For patients who do not tolerate the protocol-specified dosing schedule, dose adjustments are permitted in order to allow the patient to continue the study treatment. These changes must be recorded on the Dosage Administration Record CRF. The following guidelines will be applied:

During neoadjuvant treatment, only two dose delays of up to 2 cycles each will be allowed. In addition, a patient must discontinue treatment with the study medications if after resumimg at a lower dose treatment, the toxicity recurs with the same or worse severity (Table 6.2). For adjuvant Herceptin, patients should not miss more than 3 months of adjuvant Herceptin.

If a patient requires a dose interruption for more than 2 consecutive cycles during the neoadjuvant phase, this patient will be discontinued from the study treatment. Patients who discontinue the study treatment for a treatment-related adverse event or an abnormal laboratory value will still be followed up as described in Table 6.2.

Toxicity due to docetaxel or FEC may be managed by symptomatic treatment, dose interruptions and adjustment of docetaxel /FEC dose. Doses of medications omitted for toxicity are not replaced or restored; instead the patient should resume the planned treatment cycles. The omitted doses will be noted as treatment interruptions. Patients who miss their

treatment doses for more than 2 consecutive weeks will be classified under treatment interruptions and will not be analyzed as per protocol treatment.

Dose modification for Docetaxel

1. Myelotoxicity: if the ANC ≤1500 cells/mm^3 or platelets ≤100,000/mm^3 on day 1 of each cycle, therapy will be delayed until counts recover.
2. In case the treatment had to be postponed for 1 week due to toxicity, the dosage of docetaxel will be reduced to 75 mg/m^2 if the patient was on 100mg/m^2 or to 60mg/m^2 if the patient was on 75mg/m^2.
3. Dose adjustment for diarrhea: withhold docetaxel for grade 2 or worse diarrhea and restart at a lower dose (75 mg/m^2 if the patient was on 100mg/m^2 or to 60mg/m^2 if the patient was on 75mg/m^2) after complete resolution.
4. Patients who develop grades 3 or 4 neuropathy for a week or longer will have their doses reduced to 75 mg/m^2 if the patient was on 100mg/m^2 or to 60mg/m^2 if the patient was on 75mg/m^2. Patients who develop ≥ grade 3 neuropathy while on 60mg/m^2 should have docetaxel treatment discontinued entirely (See Table 6-3).
5. Docetaxel infusions could be slowed down or interrupted for minor symptoms such as flushing or local cutaneous reactions and resumed once symptoms abate. Similar with FEC infusions.

Note: If there is a change in body weight of at least 10%, the dosage will be recalculated.

Dose modification for FEC

1. Myelotoxicity: treatment with FEC will be delayed until the absolute neutrophil count is greater than 1500/mm^3 and platelet count is greater than 100,000/mm^3
2. If a patient develops severe neutropenia (<500/mm^3) for a week or longer, or febrile neutropenia during the prior course, then the FEC doses will be reduced by 12.5% for subsequent courses. FEC doses will be decreased by 12.5% in patients whose platelet count nadir is <25,000/mm^3 for longer than five days.

Note: If there is a change in body weight of at least 10%, the dosage will be recalculated.

Dose modifications for Herceptin SC (trastuzumab)

No reductions in the dose of Herceptin SC (trastuzumab) will be made during this clinical trial unless due to symptomatic cardiac dysfunction. Patients may continue Herceptin SC therapy during periods of reversible, chemotherapy-induced myelosuppression, but they should be monitored carefully for complications of neutropenia during this time. The specific instructions to reduce or hold the dose of chemotherapy should be followed. Trastuzumab will be permanently discontinued if severe or life-threatening (based on CTCAE grading) administration reactions occur.

Monitor LVEF clinically every 3 weeks at every visit.

Infusion Reactions: The infusion rate will be decreased for subjects experiencing mild or moderate infusion reactions according to CTCAE grading. If a subject experiences dyspnea or clinically significant hypotension the infusion will be stopped. Herceptin will be permanently discontinued if severe or life-threatening infusion reactions (based on CTCAE grading) occur.

Herceptin injection can be slowed down if patient is feeling uncomfortable and continued slowly when the patient is fine.

Cardiomyopathy: We will assess for symptoms before each cycle and evaluate cardiac toxicity by Echocardiography every 12 weeks during the neoadjuvant treatment and every 3 months during adjuvant Herceptin. Treatment with trastuzumab will be stopped for a minimum of 3 weeks (one cycle) for any of the following:

- LVEF is below 50% and ≥ 15% points decrease from baseline

If LVEF returns to 50% or higher treatment can be resumed. If LVEF decrease persists or has declined further, or if clinically significant CHF has developed, treatment with Herceptin will be stopped.

If after a repeat assessment within approximately 3-7 weeks (2 cycles), the LVEF has not improved, or has declined further, trastuzumab should be discontinued, unless the benefits for the individual patient are deemed to outweigh the risks (see appendix E).

Table 6-2. Recommendations for dose modification for docetaxel or FEC by event

Toxicity	Dose modification*
Worst toxicity (CTCAE v5)	During a cycle of therapy
No toxicity	Maintain dose level
Investigations (Hematologic)<br>*Neutropenia (ANC)*	
Grade 1 (ANC < LLN - 1500/mm^3)	Maintain dose level
Grade 2 (ANC < 1500 - 1000/mm^3)	Maintain dose level (see Table 6-3)
Grade 3 (ANC < 1000 - 500/mm^3)	Omit dose until resolved to ≤ Grade 1, then ↓ 1 dose level (Table 6-3)
Grade 4 (ANC < 500/mm^3)	Omit dose until resolved to ≤ Grade 2, then ↓ 2 dose levels
Thrombocytopenia	
Grade 1 (PLT < LLN - 75,000/mm^3)	Maintain dose level
Grade 2 (PLT < 75,000 - 50,000/mm^3)	Maintain dose level
Grade 3 (PLT < 50,000 - 25,000/mm^3)	(Lower limit PLT+ 30,000/mm^3) Omit dose until resolved to ≤ Grade 1, then<br>If resolved in ≤ 7 days, maintain dose level<br>If resolved in > 7 days, ↓ 1 dose level
Grade 4 (PLT < 25,000/mm^3)	Omit dose until resolved to ≤ Grade 1, then ↓ 1 dose level
Febrile neutropenia<br>(ANC < 1.0 x 109/L, fever ≥ 38.5°C)	Omit dose until resolved, then ↓ 1 dose level
Investigations (Renal)<br>Serum creatinine	
Grade 1 (> ULN - 1.5 x ULN)	Maintain dose level
Grade 2 (> 1.5 - 3.0 x ULN)	Omit dose until resolved to ≤ Grade 1 or baseline, then maintain dose level
Grade 3 (> 3.0 - 6.0 x ULN)	Omit dose and discontinue patient from study drug treatment

Toxicity	Dose modification*
Worst toxicity (CTCAE v5)	During a cycle of therapy
Grade 4 (> 6.0 x ULN)	Omit dose and discontinue patient from study drug treatment
*Investigations (Hepatic)**	
Bilirubin	
Grade 1 (> ULN - 1.5 x ULN)<br>Grade 2 (> 1.5 - 3.0 x ULN)<br><br><br>Grade 3 (> 3.0 - 10.0 x ULN)<br><br><br>Grade 4 (> 10.0 x ULN)	Maintain dose level<br>Omit dose until resolved to ≤ Grade 1, then:<br>If resolved in ≤ 7 days, then maintain dose level<br>If resolved in > 7 days, then ↓ 1 dose level<br>Omit dose until resolved to ≤ Grade 1, then:<br>If resolved in ≤ 7 days, then ↓ 1 dose level<br>If resolved in > 7 days, then discontinue patient from study drug treatment<br>Omit dose and discontinue patient from study drug treatment
AST or ALT	
Grade 1 (> ULN - 2.5 x ULN)<br>Grade 2 (> 2.5 - 5.0 x ULN)<br>Grade 3 (> 5.0 - 20.0 x ULN)<br><br><br>Grade 4 (> 20.0 x ULN)	Maintain dose level<br>Maintain dose level<br>Omit dose until resolved to ≤ Grade 1 (or ≤ Grade 2 if liver metastases present), then:<br>If resolved in ≤ 7 days, maintain dose level<br>If resolved in > 7 days, ↓ 1 dose level<br>Omit dose and discontinue patient from study drug treatment
AST or ALT and Bilirubin<br>AST or ALT Grade 3 and ≥ Grade 2 blood bilirubin	Omit dose and discontinue patient from study drug treatment.
Diarrhea**	

Toxicity	Dose modification*
Worst toxicity (CTCAE v5)	During a cycle of therapy
Grade 1	Maintain dose level, but initiate anti-diarrhea treatment
Grade 2	Omit dose until resolved to ≤ grade 1, then maintain dose level.<br>If diarrhea returns to ≥ grade 2, then omit dose until resolved to ≤ grade 1, then ↓ 1 dose level
Grade 3	Omit dose until resolved to ≤ grade 1, then ↓ 1 dose level
Grade 4	Omit dose and discontinue patient from study drug treatment

*All dose modifications will be based on the worst preceding toxicity.

** Antidiarrhea medication is recommended at the first sign of abdominal cramping, loose stool or overt diarrhea

Table 6-3. Recommendations for dose modification by the appearance of the adverse drug reaction

**Adverse drug reaction**	**Grade 2**	**Grade 3**	**Grade 4**
1st appearance	Interrupt treatment until resolved to grade 0-1, then continue at 1 dose level* lower than the original docetaxel or FEC dose where possible	Interrupt treatment until resolved to grade 0-1, then continue at 1 dose level* lower than original docetaxel or FEC dose, with prophylaxis where possible	Interrupt treatment, if investigator considers it to be in the best interest of the patient, continue at 2 dose level docetaxel or FEC dose, once toxicity has resolved to grade 0-1
2nd appearance of the same toxicity	Interrupt treatment until resolved to grade 0-1, then continue at 2 dose levels** lower than the original docetaxel or FEC dose	Interrupt treatment until resolved to grade 0-1, then continue at 2 dose levels** lower than original docetaxel or FEC dose	
3rd appearance of the same toxicity	Discontinue study treatment	Discontinue study treatment	
*1 dose level: Docetaxel = 75mg/m^2 if on 100mg/m^2 or 60mg/m^2 if on 75mg/m^2<br>FEC = decrease by 10% from normal dose<br>**2 dose level: Docetaxel = 60mg/m^2 if on 100mg/m^2 or discontinuation if on 75mg/m^2<br>FEC= decrease by 15% from normal dose			

6.8.1 Guidelines for the management of anaemia

Participants with chemotherapy induced anaemia should be managed as follows
PCV 29-28 % Haematinics with or without erythropoietin and reassessed after one week.
Following repeat PCV if ≤28, continue with chemotherapy but monitor closely.
PCV ≤ 27%: Blood transfusion to correct the anaemia before further chemotherapy

6.9 Disease progression during study

Patients will be taken off study treatment at the time of clinical progression.

Toxicity will be classified (Table 6-3) according to the NCI CTCAE Version 5.

6.10 Follow-up for toxicities

Patients whose treatment is interrupted or permanently discontinued due to an adverse event or clinically significant abnormal laboratory value must be followed up at least once a week (or more frequently if required by institutional practices, or if clinically indicated) for 4 weeks, and subsequently at approximately 4-week intervals, until resolution or stabilization of the event, whichever comes first. Appropriate clinical experts such as ophthalmologist, endocrinologist, dermatologist, psychiatrist etc. will be consulted as deemed necessary. All patients must be followed up for adverse events and serious adverse events for 30 days after receiving the last dose of any study agent.

6.11 Anticipated risks and safety concerns of the study treatments

The study medications have previously been in clinical use with good safety profile. In the proposed study, these medications are given in routine dose regimens and known toxicities are anticipated. These include: myelosupression, mucositis, diarrhea and vomiting, neuropathy and cardiotoxicity.

The following measures will be adopted to ensure safety of the participants:

1. Patients will be given 3 phone numbers (study nurse, PI and clinic emergency) to contact in case of complaints.
2. Clinical reviews before chemotherapy will be carried out since the patients will be seen three-weekly for chemotherapy.
3. Phone calls by the study nurse will be conducted regularly to check for complaints during the follow up period.
4. Appropriate clinical management would be given to patients with complaints.

6.12 Concomitant medications

Medications required to treat AEs, to manage cancer symptoms, concurrent diseases and supportive care agents such as packed red blood cell agents, pain medications, anti-emetics, short courses of steroids, topical treatments for stomatitis, anti-diarrheal and focal radiotherapy for palliation (not including the primary site of interest) are allowed. Biphosphonate therapy for prevention of bone metastasis, is allowed.

If biphosphonate therapy is initiated after commencement of protocol therapy, the reason will be clearly stated.

6.13 Prohibited concomitant therapy

Hormonal contraceptive agents are not indicated while on study medications.

Docetaxel therapy

Concomitant use of aminoglycosides should be avoided during treatment with docetaxel.

FEC

Medications with strong risk of cardiotoxicity should be administered with caution.

Trastuzumab (Herceptin SC) therapy

Medications that carry strong risk of cardiotoxicity should be administered with caution. If possible, physicians should avoid anthracycline-based therapy for up to 7 months after stopping Herceptin. If anthracyclines are used, the patient's cardiac function should be monitored carefully.

6.14 Patient numbering

6.14.1 Patient numbering

Each patient is identified in the study by a Subject Number (Subject No.) assigned when the patient is first enrolled for screening and is retained as the primary identifier for the patient throughout her entire participation in the trial. The Subject No. consists of the Center Number (Center No.) as assigned by HLF office to the investigation site with a sequential Subject (patient) number suffixed to it, so that each subject is numbered uniquely across the entire database. Upon signing the informed consent form, the patient is assigned to the next sequential Subject No. available.

6.14.2 Treatment assignment

N/A

6.14.3 Treatment blinding

This is an open label study.

6.15 Study drug preparation and dispensation

Study drugs are given intravenously or subcutaneously as described above and will be administered to the patients by study site authorized personnel only. All dosages prescribed to the patient and all dose changes during the study must be recorded on the Dosage Administration Record CRF.

6.16 Drug supply and storage

Study drugs will be received by designated personnel at the study site, handled and stored safely and properly, and kept in a secured location to which only the investigator and designated site personnel have access. Upon receipt, the study medication will be stored according to the instructions specified on the drug labels and in the Investigators' Brochure .

6.17 Study drug accountability and compliance

The investigator or designee must maintain an accurate record of the shipment and dispensing of study medication in a drug accountability log. Drug accountability will be noted by the field monitor during site visits and at the completion of the study. At the time of study close-out and, if appropriate during the course of the study, the investigator will return all used and unused study drugs, packaging, drug labels, and a copy of the completed drug accountability log to the Study PI or Study Monitor.

7. Study flow and visits schedule

Table 7-1 lists all of the assessments; the visits indicated with an "X" when they are performed. All data obtained from these assessments must be supported by record in the patient's source documentation.

For all visits, there is a ± 3 days window on assessments to take into account scheduling over public or religious holidays if not explicitly specified otherwise.

Laboratory assessments that were completed within 3 days prior to Cycle 1 Day 1 (C1D1) do not need to be repeated. For efficacy assessments, there is a ±7 day window (refer to Section 7.2.1). Screening USS, chest x-ray and pre-treatment tumor biopsy will be completed within 14 days before first dose of study drug administration.

All data obtained from these assessments will be supported by the record in the patient's source documentation. No CRF will be used as a source document.

7.1 Screening

The approved Informed Consent Form (ICF) will be signed and dated before any screening procedures are performed (procedures that are part of the clinical routine during the initial diagnostic work-up of the patient may be performed before signing the ICF). A copy of the ICF will be given to the patient or to the person signing the form. The date when the study informed consent was signed will be registered in the medical record of the patient by investigator or designee.

Patient's eligibility will be evaluated by study inclusion and exclusion criteria and safety assessments.

7.2 Details of assessment

For details of assessments, refer to Table 7-1. All screening assessments will be completed within 14 days prior to the first dose.

Table 7-1 Visit evaluation schedule (Neo-Adjuvant). Protocol Section 7.2

**Assessment**	**Screening**	**Part 1 and 2: 3-Weekly visit**										
**Sequential visit number**	**Visit 1**[1]	**V2**	**V3**	**V4**	**V5**	**V6**	**V7**	**V8**	**V9**	**V10**	**V11**	**V12**
**Calendar visit day**		**1**	**22**	**43**	**64**	**85**	**106**	**127**	**148**	**169**	**190**	**211**
		**+/- 3 days**										
Obtain informed consent	X											
Patient history and epidemiological data	X											
Demographics	X											
Inclusion/exclusion criteria	X											
Relevant medical history/current medical conditions	X											
Diagnosis and extent of cancer	X											
Prior antineoplastic therapy	X											
Prior/concomitant medications	X											
Physical examination	X	X	X	X	X	X	X	X	X	X	X	X
Performance status	X											
Height	X											
Weight	X	X	X	X	X	X	X	X	X	X	X	X
Vital signs	X	X	X	X	X	X	X	X	X	X	X	X

**Assessment**	**Screening**	**Part 1 and 2: 3-Weekly visit**										
**Sequential visit number**	**Visit 1**[1]	**V2**	**V3**	**V4**	**V5**	**V6**	**V7**	**V8**	**V9**	**V10**	**V11**	**V12**
**Calendar visit day**		**1**	**22**	**43**	**64**	**85**	**106**	**127**	**148**	**169**	**190**	**211**
		**+/- 3 days**										
Hematology	X	X	X	X	X	X	X	X	X	X	X	X
Chemistry	X	X	X	X	X	X	X	X	X			
Urinalysis	X											
Pregnancy test	X											
Imaging												
Breast Ultrasound	X			X		X[2]		X	X[3]			
Chest x-ray	X											
Ultrasound (abdomen/pelvis)	X											
Bone scan	X	As clinically indicated										
CT scan of brain	As clinically indicated											
CT scan of chest, abdomen, pelvis	As clinically indicated											
ECHO every 12 weeks while on neoadjuvant therapy	X					X				X		
EKG	X											
Adverse events	Continuous assessment											

**Assessment**	**Screening**	**Part 1 and 2: 3-Weekly visit**										
**Sequential visit number**	**Visit 1**[1]	**V2**	**V3**	**V4**	**V5**	**V6**	**V7**	**V8**	**V9**	**V10**	**V11**	**V12**
**Calendar visit day**		**1**	**22**	**43**	**64**	**85**	**106**	**127**	**148**	**169**	**190**	**211**
		**+/- 3 days**										
Blood for "Omics biomarkers"	X					X				X		
Blood for PK studies (See SOP for PK studies	X		X	X	X		X				X	X
Biomarkers (ER, PgR, HER-2)	X											
Docetaxel IV every three weeks		X	X	X	X							
Herceptin SC		X	X	X	X	X[2]	X[2]	X[2]				
FEC (as indicated)[2]						X	X	X				
LHRH agonist every 3 months for 2 yrs (HR+ premenopausal pts)		X										
Daily TAM or LET for HR+pts after surgery (or LHRH agonist) x 10 yrs.									X			
QoL	X	X	X	X	X	X	X	X				

**Assessment**	**Screening**	**Part 1 and 2: 3-Weekly visit**										
**Sequential visit number**	**Visit 1**[1]	**V2**	**V3**	**V4**	**V5**	**V6**	**V7**	**V8**	**V9**	**V10**	**V11**	**V12**
**Calendar visit day**		**1**	**22**	**43**	**64**	**85**	**106**	**127**	**148**	**169**	**190**	**211**
		**+/- 3 days**										

[1] All screening procedures must be done within 14 days of first dose of study therapy.

[2] Patients with USS CR (operable) will have surgery within 4 weeks of last dose of Docetaxel+Herceptin. Patients with USS PR/SD (operable) will get salvage therapy with FEC+H for 3 cycles. Patients with inoperable SD will also get salvage therapy with FEC +H for 3 cycles, but will be considered non-responders for statistical analysis purposes. Patients showing clear progression will be taken off study treatment and managed per treating physician.

[3] Patients with USS PR/SD (operable) will have surgery within 4 weeks of last dose of FEC+H. Patients with SD (inoperable) or PD during/after FEC treatment will be taken off study treatment, managed per treating physician and scheduled for End of Treatment visit, but will have continued follow-up unless the patient withdraws from the study.

Table 7-2: Visit evaluation schedule (Adjuvant Therapy) for Complete/Partial Responders/SD (operable)

Assessment	Category	Adjuvant 3-weekly visits for SC Herceptin									
**Sequential visit number**		**1**	**2**	**3**	**4**	**5**	**6**	**7**	**8**	**9**	**10**
**Calendar Visit Day**		**1**	**22**	**43**	**64**	**85**	**106**	**127**	**148**	**169**	**190**
Vital signs		X	X	X	X	X	X	X	X	X	X
Hematology		X	X	X	X	X	X	X	X	X	X
Chemistry		X	X	X	X	X	X	X	X	X	X
Surgery		X									
Collection of fresh tumor and FFPE (at baseline and after surgery)		X									
Blood for PK studies (See SOP for PK studies)		X	X	X	X	X	X	X	X	X	X
Radiotherapy (Estimated 4-6 weeks after surgery)				X							
ECHO every 3 months while on adjuvant Herceptin		X			X			X			X
Herceptin SC every 3 wks to complete 18 doses		X	X	X	X	X	X	X	X	X	X
LHRH agonist every 3 months for 2 yrs (HR+ premenopausal pts)		X			X			X			X
Safety assessment based on ECHO results for patients on Herceptin every 12 wks.		X		X		X		X		X	
Adverse events		Continuous monitoring									
Bone scan		As clinically indicated									

CT scan of brain		As clinically indicated						
CT scan of chest, abdomen, pelvis		As clinically indicated						
Tumor pathologic response evaluation based on histopathology of resected tumor		X						

Table 7-3 Visit evaluation schedule- End of treatment visit and follow up

	Follow-up Period (10 Years Total)						
	Safety Follow-up		Survival Follow-up				
	60 Days		Years 1-2		Years 3-5	Years 6-10	
**Assessment**	**End of Study Treatment (EoT) (+/- 3 days)**	**30 Days post EoT (+/- 3 days)**	**Every 3 months[1]**	**Every 6 months**	**Every 6 months**	**Every 6 months**	**Every 12 months**
Physical Examination	X	X	X		X	X	
Vital Signs	X	X	X		X	X	
Laboratory Assessments							
Hematology	X	X	X		X	X	
Chemistry	X	X	X		X	X	
Blood for "Omics biomarkers"	X	X	X		X	X	
Blood for PK studies (See SOP for PK studies	X	X	X	X	X	X	
ECHO	X			X	X	X	
Imaging							
Bone scan	As clinically indicated						
CT scan of brain	As clinically indicated						
CT scan of chest, abdomen, pelvis	As clinically indicated						
Adverse Events	X	X					
QoL		X		X		X	
Daily TAM/LET for HR+ patients after surgery (or LHRH agnoist) for 10 years	X -- X						
Survival Follow-up			X		X		X

1. First Survival contact is at Month 3 post EoT

7.1.1.1 General Guidelines

Prior to registration and any study-specific evaluations being performed, all patients must have given written informed consent for the study and must have completed the pre-treatment evaluations. Patients must meet all of the eligibility requirements listed in Section 3.

Eligible patients will be registered on study centrally by HLF staff.

Following registration, patients should begin protocol treatment within 7 business days. Issues that would cause treatment delays should be discussed with the Lead Principal Investigator. If a patient does not receive protocol therapy following registration, the patient's registration on the study will be canceled. The study coordinator/CRA should be notified of cancellations as soon as possible.

7.1.1.2 Registration Process

When a potential patient has been identified, enrolling site must notify HLF staff by calling 234-805-660-2274. Reservations for potential subjects will only be held for subjects who have signed consent for that particular study.

The following information will be included:

- Study title
- Name of study site where patient is being treated
- Name of treating physician
- Name of site PI
- Patient's initials
- Patient's date of birth
- Patient's height in centimeters, weight in kilograms and ECOG performance status
- Proposed treatment start date
- Date of signed consent
- Eligibility criteria met (no, yes)

A confirmation of registration will be issued by HLF staff after eligibility is confirmed.

7.1.2 HLF Data Submission

Data reporting will be performed utilizing the REDCap electronic data capture system. The University of Chicago staff will provide applicable user registration information.

Each screened (consented) patient is to be entered into REDCap within 48 hours of patient registration. In addition to direct data entry, you may be required to provide supporting source documentation. Source records are original documents, data, and records (e.g., medical records, raw data collection forms, pharmacy dispensing records, recorded data from automated instruments, laboratory data) that are relevant to the clinical trial. Each site

will prepare and maintain adequate and accurate source documents. These documents are designed to record all observations and other pertinent data for each subject enrolled in this clinical trial. Source records must be adequate to reconstruct all data transcribed onto the case report forms.

All required data must be recorded in the REDCap database at the completion of each cycle. AEs (including SAEs) are to be entered in real time. SAEs are to be sent via email (preferred) or fax to the University of Chicago (PhaseIICRA@medicine.bsd.uchicago.edu and qaccto@bsd.uchicago.edu; Fax: 773-702-4889) within 24 hours of HLF's knowledge of the event.
Forms will be submitted by sites to the HLF Data Management Center (DMC) in compliance with the Data Submission schedule below for entry into REDCap. There are two options for submitting forms.

- The forms may be faxed to the DMC at (234)-2-234-2-8101371. Copies must be 100% of the original form size
- The forms may be mailed/faxed to the HLF:

  Healthy Life For All Foundation (HLF) Office

  P.O. Box 30175 Secretariat, Ibadan, Oyo State, Nigeria
  Tel/Fax: 234-8056602274

  E-mail Dayo Adepoju: dayoadepoju@yahoo.com

Data Submission Schedule

Form	***Submission schedule***
On-Study Form-Pathology and biopsy reports	Within one week of registration
Tissue Block with Pathology Reports	Submit with paraffin block to HLF LAB and a copy to the HLF DMC.
Visit form (Neo-adjuvant Treatment Summary Form)	Submit once at the end of each protocol specific chemotherapy for each patient within 48 hours
Adverse Events Form	Submit for each patient on same day of detection.
Quality of Life Assessment Form	Submit at the end of each clinic visit for study drug administration for all patients within 48 hours. Thereafter every 3 months during follow up visits.
Follow-up Form	On completion or discontinuation of protocol therapy, submit every 3 months for 2 years. In addition, submit at any significant clinical event.

7.1.4 Patient demographics and other baseline characteristics

Data to be collected will include general patient demographics, relevant medical history and details of prior anti-neoplastic treatments, current medical conditions, concomitant medications, prior cancer therapies, diagnosis, extent of tumor and tumor histopathology including hormonal and HER2 statuses.

7.2 Treatment period

During the treatment period, the patient must follow the Investigators instructions with regards to contraception, concomitant medications, and dosing regimen (see Section 6.1).

If a patient remains on study after a dose interruption for > 14 days, such patient will not be assessed as per protocol treatment. For details of the frequency of the visits and assessments during the treatment period, refer to Tables 7-1 & 7-2.

7.3 End of treatment visit including study completion and premature withdrawal

Patients who discontinue study treatment will be scheduled for an End of Treatment (EOT) visit as soon as possible and within 14 days after the decision to discontinue has been made, at which time all of the assessments listed for the EOT visit will be performed. An EOT CRF page will be completed. The date and primary reason for stopping the study treatment will be recorded. At a minimum, all patients who discontinue study treatment, including those who refuse to return for a final visit, will be contacted for safety evaluations during the 30 days following the last dose of study treatment.

Patient who discontinue study treatment will also return for 30-day safety F/U (see Section 7.5.1), and survival F/U assessments at the visits according toTable 7-3 and will not be considered withdrawn from the study. If patient refuses to return for these visits or is unable to do so, every effort will be made to contact this patient or a knowledgeable informant by telephone to obtain the F/U information.

If a patient discontinues study treatment, but continues study assessments in the F/U period, the patient remains on a study until completion of the protocol criteria for ending study assessments.

7.4 Criteria for premature patient withdrawal

Patient may voluntarily withdraw from the study or be dropped from the study at the discretion of the investigator at any time. Patient may be withdrawn from the study if any of the following occur:

- Any life threatening adverse event (Section 8.2)
- Delay in dosing for > 2 cycles
- Patient withdrawal of consent
- Lost to follow-up
- Disease progression
- Initiation of a different cancer therapy.

7.4.1 Replacement policy

Patients will not be replaced on the study. However, if a subject is considered as non-evaluable for the neoadjuvant treatment response assessment, enrollment of a new subject as a replacement will be considered to meet the required number of evaluable subjects for statistical validity. Enrollment of new subjects may be considered until the required number of evaluable subjects on neoadjuvant regimen is achieved.

7.5 Follow up period

Patient lost to follow up will be recorded as such on the CRF. For patient who is lost to follow-up, the investigator will show "due diligence" by documenting in the source documents steps taken to contact the patient, e.g., dates of telephone calls, home visits if feasible, etc.

7.5.1 30 - day safety follow up period

All patients will have safety evaluations at 30 days after the last dose of study treatment. Information related to AEs (including concomitant medication taken for ongoing AEs) and ongoing anti-neoplastic treatments will be collected at 30 days after the last dose of study drug. All AEs suspected to be related to study treatment should be followed up weekly, or as clinically indicated, until resolution or stabilization.

7.5.2 Survival follow up period

All patients enrolled in the study will be followed for survival every 3 months by clinic appointments or by phone call for 2 years and thereafter every 6 months for next 3 years, and yearly for 5 years (10 years total), or until lost to follow-up or withdrawal of consent, whichever occurs first.

7.5.3 Participants retention and follow up compliance

To ensure retention and follow up, we have developed a robust infrastructure including trained nurses, social workers and psychologists as members of the multidisciplinary breast cancer team. While patients will receive free treatment on this clinical trial, we must avoid inducements that can be interpreted as coercion. Nonetheless, we appreciate hardship on patients without resources to come to clinic appointments. We will provide funds for transport and will give recharge cards not to exceed N3, 000 (~$10) after each clinic visit so that we can actively monitor patients and follow up for complications. In addition to regular phone calls, we will also engage patient navigators who will monitor the patients closely for clinic compliance and undertake home visits if necessary. In addition, we will integrate psychosocial counseling and behavioral interventions by psycho-oncologists and counsellors. These measures will ensure optimal compliance to treatment and follow up of more than 80%. These measures and projections are made following a meta-analysis of compliance figures in clinical trials in patients with tuberculosis (TB) in sub-Sahara Africa. In that report, TB treatment compliance and hence the outcome improved to more than 80% when patient education, healthcare provider education, incentives and enablers, psychological interventions, reminders and tracers were employed (Alipanah et al., 2018). We will incorporate these strategies to improve compliance.

7.6 Assessment types

7.6.1 Efficacy assessments

Tumor response will be evaluated locally by the investigator based on the adapted RECIST criteria (see Appendix B). The largest breast tumor lesion with the largest involved axillary lymph node will be assessed at baseline by ultrasound technique. The following assessments are required at screening/baseline (within 14 days of the start of treatment):

1. Breast ultrasound, abdomen and pelvis ultrasound
2. If bone metastases are suspected, localized or skeletal survey by plain x-ray imaging will be acquired for all skeletal lesions suspected clinically

Subsequent tumor evaluations will be performed per the schedule outlined in Table 7-1 or as clinically indicated. If the last prior tumor evaluation was within 28 days of EOT or objective

evidence of progressive disease has already been documented, the tumor evaluation does not need to be repeated at EOT.

Tumor evaluations after the screening assessment will include evaluation of all sites of disease identified at baseline, using the same technique that was used at screening. If there was no evidence of disease in a body region at baseline, this region does not need to be imaged at subsequent assessments, unless there is clinical concern for a new lesion in that body region.

7.6.2 Safety and tolerability assessments

Safety will be monitored by assessing the procedures listed below as well as collecting of the adverse events at every visit. All safety assessments will be performed pre-dose unless specified otherwise.

Physical examination

A complete physical examination that evaluates all major organ systems will be performed at a time of screening/baseline. Subsequent physical exams will be done if indicated and may be limited to focusing on sites of disease to evaluate clinical signs and symptoms.

Significant findings that were present prior to the signing of informed consent must be included in the Relevant Medical History/Current Medical Conditions page on the patient's CRF. Significant new findings that begin or worsen after informed consent must be recorded on the Adverse Event page of the patient's CRF.

Physical exams to be performed are outlined in Tables 7-1, 7-2 & 7-3.

Vital signs

Vital signs (heart rate, blood pressure and temperature) will be obtained in the sitting position, or as appropriate prior to any blood collection.

Vital signs will be performed as outlined in Tables 7-1, 7-2 & 7-3.

Height and weight

Height in centimeters (cm) and body weight (approximated to 0.1 kilogram (kg) in indoor clothing, but without shoes) will be measured.

Height and weight to be performed as outlined in Tables 7-1, 7-2 & 7-3.

Performance status

The ECOG Performance Status will be assessed at screening and regularly throughout the whole study period irrespective of the time of dosing (refer to Appendices B, C).

Performance status will be assessed as outlined in Tables 7-1, 7-2 & 7-3.

Laboratory evaluations

Study sites will use local laboratories for the analysis of all safety lab samples collected at the time points as indicated in Tables 7-1, 7-2 & 7-3. More frequent assessments may be performed if clinically indicated, or at the investigator's discretion, and these will be recorded on the Unscheduled Visit CRF page.

An abnormal laboratory value that is clinically relevant (e.g., requires an interruption or delay in study treatment, or leads to clinical symptoms, or requires therapeutic intervention) must be documented in the Adverse Event CRF. If any abnormal laboratory value constitutes an AE, it must be recorded on the Adverse Event CRF.

Hematology

The following will be assessed:

Complete blood count (CBC) and differential blood count (DBC) including white blood count (WBC), neutrophil count (including bands), lymphocyte, monocyte, eosinophil, and basophil counts, hemoglobin, and platelet.

Clinical chemistry

The following will be assessed:

Sodium, potassium, chloride, bicarbonate, urea or BUN, creatinine, AST (SGOT), ALT (SGPT) and total bilirubin. If a total bilirubin elevation of ≥ Grade 2 occurs, direct and indirect bilirubin will be measured. Clinical chemistry collections will be performed as outlined in Tables 7-1, 7-2 & 7-3.

Urinalysis

A macroscopic urinalysis (dipstick or other equivalent method according to local practice), including leukocytes, protein, glucose and blood will be performed.

Abnormal findings will be followed up with a microscopic evaluation and/or additional assessments as clinically indicated.

Urine collection will be performed as outlined in Table 7-1.

Pregnancy and fertility assessments

When effective contraception is required, the pregnancy testing is recommended at screening and/or pre-dose and at the end of the trial. At screening (or baseline), urine pregnancy test should be performed, while at the end of trial the urinary pregnancy tests are sufficient.

Echocardiogram (ECHO)

Echocardiogram will be performed at the time of screening and/or baseline, then every 6 weeks and continue every 3 months while patient is on Herceptin alone. Thereafter ECHO should be performed every 6 months for the first 2 years after Herceptin treatment.

ECHO will be performed by a qualified physician and documented on the ECHO CRF page. Each ECHO result will be labeled with the study number, patient initials (per regulations

permit), patient ID number and date. These records will be kept in the source documents at the study site. Clinically significant abnormalities existing at the time of the patient signing the informed consent will be reported on the Medical History CRF page. Clinically significant findings will be discussed with the Steering Committee Chairman and Internal Monitor Breast Cancer Group prior to enrolling the patient in the study. New or worsened clinically significant findings occurring after signing the informed consent will be recorded on the Adverse Events CRF page.

7.7 Patient reported outcomes

7.7.1 The EORTC Quality of Life questionnaire

Questionnaires of The EORTC general quality of life QLQ-C30 version 3.0 and Breast Cancer Module QLQ-BR23 English Language and Yoruba Language versions will be used in the study (Appendices B and D).

It is composed of scales that assess physical functioning, role functioning as well as emotional, cognitive, social and global health statuses assessment. There are also scales to assess various symptoms. The breast cancer module has items to assess outcomes peculiar to breast cancer and treatment such as body image, sexual functioning and future perspective. The questionnaires will be completed by the patient/or with the assistance of the study nurse while waiting to receive their chemotherapy. The study nurses and the investigators will be available for questions about the study and completion of the forms.

8. Safety monitoring and reporting

8.1 Adverse events

8.1.1 Definitions

An adverse event is defined as the appearance of (or worsening of any pre-existing) undesirable sign(s), symptom(s), or medical condition(s) that occur after patient's signed informed consent has been obtained.

Abnormal laboratory values or test results occurring after informed consent has been signed constitute adverse events only if they induce clinical signs or symptoms, are considered clinically significant, require therapy (e.g., hematologic abnormality that requires transfusion or hematological stem cell support), or require changes in study medication(s).

Adverse events that begin or worsen after informed consent will be recorded in the Adverse Events CRF. Conditions that were already present at the time of informed consent will be recorded in the Medical History page of the patient's CRF. Adverse event monitoring will be continued for at least 30 days following the last dose of study treatment. Adverse events (including lab abnormalities that constitute AEs) will be described using a diagnosis whenever possible, rather than individual underlying signs and symptoms. When a clear diagnosis cannot be identified, each sign or symptom will be reported as a separate Adverse Event.

Adverse events will be assessed according to the Common Terminology Criteria for Adverse Events (CTCAE) version 5.0

If CTCAE grading does not exist for an adverse event, the severity of mild, moderate, severe, and life-threatening, corresponding to Grades 1 - 4, will be used. CTCAE Grade 5 (death) will not be used in this study; rather, information about deaths will be collected through a Death Form.

The occurrence of adverse events will be sought by non-directive questioning of the patient (subject) during the screening process after signing informed consent and at each visit during the study. Adverse events also may be detected when they are voluntarily reported by patient (subject) during the screening process or between visits, or through physical examination, laboratory test, or other assessments.

Each adverse event will be evaluated to determine:

1. Severity grade (CTCAE Grade 1-4)
2. Duration (start and end dates) and association with study therapy (not related, unlikely, possible, probable, definite)
3. Action taken with respect to study treatment: none, dose adjusted, temporarily interrupted, permanently discontinued, unknown, not applicable.
4. Whether medication or therapy was given: no concomitant medication/non-drug therapy, concomitant medication/non-drug therapy.
5. Outcome: not recovered/not resolved, recovered/resolved, recovering/resolving, recovered/resolved with sequalae, fatal, unknown.
6. Whether it is serious or not. AE or SAE is defined in Section 8.2.1.

All adverse events will be treated appropriately. Any concomitant medication or non-drug therapy given to the patient will be recorded on the Adverse Event CRF.

Once an adverse event is detected, it will be followed up until its resolution or until it is judged to be permanent, and assessment will be made at each visit (or more frequently, if necessary) of any changes in severity, the suspected relationship to the study treatment, the interventions required to treat it, and the outcome.

Safety reports and actions taken will be made by the study site Co-PI to study PI and HLF simultaneously. The study PI will inform the Steering Committee Chairman.

Progression of malignancy (including fatal outcome) will be documented using the appropriate method (for example, according to the adapted RECIST criteria for solid tumors). Adverse events separate from the progression of malignancy (for example, deep vein thrombosis at the time of progression or hemoptysis concurrent with finding of disease progression) will be reported according to standard guidelines developed for such events with proper attribution regarding relatedness to the drug.

8.2 Serious adverse event

8.2.1 Definition

Serious adverse event (SAE) is defined as one of the following:

1. It is fatal or immediately life-threatening
2. Results in persistent or significant disability/incapacity
3. Constitutes a congenital anomaly/birth defect
4. It is medically significant, i.e., defined as an event that jeopardizes the patient or may require medical or surgical intervention to prevent one of the outcomes listed above
5. Requires inpatient hospitalization or prolongation of existing hospitalization.

Note that hospitalization for the following reasons should not be reported as SAE:

1. Routine treatment or monitoring of the studied indication, not associated with any deterioration in condition, e.g. treatment for disease progression
2. Elective or pre-planned treatment for a pre-existing condition that is unrelated to the indication under study and has not worsened since signing the informed consent
3. Social reasons and respite care in the absence of any deterioration in the patient's general condition

Note that treatment on an emergency outpatient basis that does not result in hospital admission lasting greater than 24 hours or involves an event not fulfilling any of the definitions of a SAE listed above is not a SAE.

8.2.2 Reporting

All events meeting SAE definitions above require reporting regardless of attribution. SAEs will be followed until resolution or until clinically relevant improvement or stabilization. SAE collection ends 30 days after the last dose of study agent(s).

To ensure patient safety, every SAE, regardless of suspected causality, occurring after the patient has provided informed consent and until at least 30 days after the patient has stopped the study treatment must be reported as described in section 8.5.

Any SAE experienced after this 30 day SAE follow-up period will only be reported if the investigator suspects a causal relationship to the study treatment.

Recurrent episodes, complications, or progression of the initial SAE must be reported as a follow-up to the original episode as described in Section 8.5.

Any event meeting SAE criteria and occurring at a different time interval or otherwise considered to be completely unrelated to a previously reported one will be reported as a new event.

Information about all SAEs is collected and recorded on the Adverse Event Report Form; all applicable sections of the form must be completed in order to provide a clinically thorough

report. The investigator must assess and record the relationship of each SAE to each specific part of the study treatment.

Suspected Unexpected Serious Adverse Reactions (SUSARs) will be collected and reported to the Nigerian National Agency for Food and Drug Administration (NAFDAC) by the Country-PI, Dr Ntekim as follows:

Life threatening SUSARs or death: within 7 calendar days after first knowledge by the sponsor. Detailed report will follow within additional 8 calendar days.

All other SUSARs that are not fatal or life threatening: Within 15 calendar days after first knowledge by medical PI

Follow up information will be actively sought and submitted as it becomes available.

8.3 Pregnancies

Pregnancy is not encouraged during the study period. Barrier method of contraception or intrauterine contraceptive devices (IUCDs) must be taken by every patient at risk of pregnancy. Every patient will be educated on importance and obligation to use contraception during the study treatment and 7 months after discontinuation of Herceptin study treatment.

To ensure patient safety, each pregnancy occurring while the patient is on study treatment or within 7 months from last dose of Herceptin during follow-up period must be reported to the investigator/treating physician. Site staff are responsible for reporting the pregnancy to HLF within 72 hours of learning of this event. HLF will report the pregnacy to the Lead PI and University of Chicago PCCC and Cancer Center staff as described in section 8.5.

The pregnancy will be followed up to determine outcome, including spontaneous or voluntary termination, details of the birth, and the presence or absence of any birth defects, congenital abnormalities, or maternal and/or newborn complications.

Pregnancy follow-up will be recorded in the study database and will include an assessment of the possible relationship to the study treatment and pregnancy outcome. Any SAE experienced during pregnancy will be reported as described as described in Section 8.5.

8.4 Routine Adverse Event Reporting

All Adverse Events **must** be reported in routine study data submissions. **AEs reported using the Serious Event Reporting Form and/or MedWatch Form discussed below must also be reported in routine study data submissions.**

All adverse events (except grade 1 and 2 laboratory abnormalities that do not require an intervention), regardless of causal relationship, are to be recorded in the case report form and source documentation. The Investigator must determine the intensity of any adverse events according to the NCI Common Terminology Criteria for Adverse Events (CTCAE) Version 4.0 and their causal relationship.

8.4.1 Reporting of Study-Specific Adverse Events and Serious Adverse Events

Heart failure

Symptomatic left ventricular systolic dysfunction (otherwise referred to as heart failure) should be reported as an SAE. If the diagnosis is heart failure it should be reported as such and not as individual signs and symptoms thereof. In the eCRF, signs and symptoms should be recorded. Heart failure should be graded according to NCI-CTCAE v 4.0 for "heart failure" (Grade 2, 3, 4 or 5) and in addition the New York Heart Association (NYHA) classification (Appendix F). 'Left ventricular systolic dysfunction' should not be used to describe symptomatic dysfunction as per NCI –CTCAE v.5.0. Heart failure occurring during the study and up to 10 years after last administration of study medications must be reported irrespective of causal relationship and followed until one of the following occurs: resolution or improvement to baseline status, no further improvement can be expected, or death.

Asymptomatic Left Ventricular Systolic Dysfunction

Asymptomatic declines in LVEF should not be reported as AEs since LVEF data are collected separately in the eCRF. Exceptions to this rule are as follows:

• An asymptomatic decline in LVEF ≥10 percentage-points from baseline to an LVEF

< 50% must be reported as an adverse event with the term of 'ejection fractiondecreased' as per NCI-CTCAE v5.0 and, in addition, a comment in the AE comments field should confirm that this was asymptomatic.

• An asymptomatic decline in LVEF requiring treatment or leading to discontinuation of pertuzumab/placebo and trastuzumab must also be reported.

Please note that this AE should also be captured as a non-serious event of special interest on the SAE form.

Table 8.1 summarizes the reporting conventions for left ventricular systolic dysfunction**.**

Table 8.1: Reporting Conventions for Left Ventricular Systolic Dysfunction/Heart Failure

Observation	How to Report	Term to be Reported	Grading
Asymptomatic decline in LVEF < 10%-points from baseline or to an LVEF≥ 50%	No additional reporting required, LVEF results to be reported on eCRF	N/A	N/A

Asymptomatic decline in LVEF ≥ 10%-points from baseline to an LVEF < 50%	AE (eCRF AE e-form)	ejection fraction decreased (a)	NCI CTCAE for “ejection fraction decreased”
Asymptomatic decline in LVEF requiring treatment or leading to discontinuation ofstudy drug	AE (eCRF AE e-form) and complete SAE form and indicate as AE of special interest	ejection fraction decreased (a)	NCI CTCAE for “ejection fraction decreased”
Heart failure (symptomatic left ventricular systolic dysfunction)	AE (eCRF AE e-form) and SAE (SAE form)	“Heart failure”	NCI CTCAE for "heart failure" and NYHA Class

Any symptomatic Left Ventricular Systolic Dysfunction event must be reported as “heart failure”

[a] Report the status “asymptomatic” and the LVEF value in the comments field as appropriate

Please refer to the algorithm to assist the decision as to whether to initiate (after completion of anti-HER2 therapy), continue or discontinue anti-HER2 study medication based on LVEF assessment in asymptomatic patients (see Appendix E).

8.5 Serious Adverse Event Reporting to the Coordinating Center

Use the UC CCC protocol number and the protocol-specific patient ID assigned during trial registration on all reports.
All serious adverse events (as defined in section 8.2.1) and pregnancy (as described in section 8.3) occurring on this study require expedited reporting to the Lead PI and University of Chicago Comprehensive Cancer Center (UC CCC).

Responsible site staff must report the event to HLF within 24 hours of site knowledge of the event. HLF will be responsible for reporting the event to the Lead PI and UC CCC as described below within 24 hours of receiving the report from the site.

Reports must be sent by HLF to the following:
PhaseIICRA@medicine.bsd.uchicago.edu Fax: 773-702-4889 AND
qaccto@bsd.uchicago.edu

All serious adverse events should also be reported to the local IRB of record according to their policies and procedures.

8.6 Serious and Unexpected Adverse Event reporting by the Coordinating Center

HLF staff will notify sites of all significant safety updates occurring during the study.

8.7 Warnings and precautions

No evidence available at the time of the development of this study protocol indicated that special warnings or precautions are required, other than those noted in this protocol and in the individual drug information leaflets provided by the manufacturers. Additional safety information collected throughout the study period will be included in the patient informed consent form and will be discussed with the patient during the study as needed.

9. Statistical methods and data analysis

The data from all participating centers in this protocol will be combined. The primary analysis of study data will be conducted at the time when all patients have completed surgery, or discontinued treatment. These data will be summarized in the primary Clinical Study Report (CSR). The final analysis of study data will be conducted at the end of the study. All available data from all patients will be analyzed and reported.

9.1 Analysis sets

9.1.1 Full Analysis Set (FAS)

The FAS will include all patients who received at least one dose of docetaxel with Herceptin as well as appropriate hormonal agentswith the exception that patients who miss their treatment doses for more than 2 consecutive weeks will be classified as non-evaluable and will not be included in the FAS.

Patients who were screened but never started treatment will be listed.

Unless otherwise specified, the FAS will be the default analysis set used for all efficacy analyses, including the primary analysis.

9.1.2 Safety Set

The Safety Set (SS) will include all patients who received at least one dose of docetaxel and have at least one valid post-baseline safety assessment. The statement in AE CRF that a patient has no AE constitutes a valid safety assessment. The SS will be used for all toxicity analyses.

9.2 Patient demographics and other baseline characteristics

Demographics and other baseline data will be summarized descriptively in tables and charts in the FAS and SS.

Categorical data will be presented as frequencies and percentages. For continuous data, mean, standard deviation, median, 25th and 75th percentiles, minimum, and maximum will be recorded.

9.3 Treatment (study treatment, concomitant therapies, compliance)

The actual dose and duration in days of TscH plus FEC + scH treatment as well as the dose intensity (computed as the ratio of total dose received to actual duration) will be listed and summarized for all patients. In other words, if patient misses some doses or received lower doses than planned, the actual dose and duration will be different from those that were planned.

Concomitant medications and significant non-drug therapies prior to and after the start of the study treatment will be summarized for all patients.

9.4 Primary objective

The primary objective of this trial is to evaluate the efficacy of every three-weekly docetaxel + Herceptin SC for four cycles, possibly supplemented by FEC. Patients achieving complete clinical response after docetaxel + Herceptin SC will go to surgery while patients with stable disease or partial response will receive 3 additional cycles of FEC + Herceptin before surgery. Inoperable, as well as progressing patients after 4 cycles of T+scH followed by 3 cycles of FEC will be taken off study treatment. Pathological complete response is defined as the absence of invasive and in-situ neoplastic cells at microscopic examination of the primary tumor and lymph nodes at surgery. Any remaining in-situ lesions are permissible.

9.5 Primary Outcome Measure

The primary statistic used to evaluate the efficacy of the study regimen is the overall pathologic complete response rate (pCR), defined as the proportion of patients with a pCR as defined in section 9.4 above. Patients who achieve pCR with the addition of FEC + scH will be included in the primary end point analysis and counted as successes, whereas those who are declared inoperable or who have progressed will be counted as failures.

Statistical hypothesis, model, and method of analysis

The primary efficacy analysis will be performed using the FAS. A one-stage design will be used. Let p_0 be the response proportion (pCR rate) that, if true, clearly implies that the treatment does not warrant further study and let p_1 be the response proportion that, if true, would imply that the treatment has sufficient efficacy to warrant further trials. The statistical analysis of a single-arm phase II trial tests the null hypothesis H_0 : pCR $\leq p_0$ against the alternative hypothesis H_1 : pCR $\geq p_1$. We choose a design given a predetermined level of power ($1 - \beta$), significance level (α), and sample size (N). We decide in favor of the hypothesis corresponding to p_0 or p_1 based on the success rate observed by using an appropriate cut-off (R) between $p_0 \times N$ and $p_1 \times N$. If *R* or fewer responses occur in the *N* patients, the drug is rejected.

For this study we expect an inefficient treatment to elicit 20% or fewer positive responses ($p_0 = 0.20$) and a successful treatment regimen to have at least 40% positive responses ($p_1 = 0.40$). This is based on a reported pCR of 46.8% and 56.3% for stages III and II Brazilian breast

cancer patients treated with neoadjuvant trastuzumab and chemotherapy (Buzatto et al., 2017). To achieve 90% power at a one-sided α=0.05, we will need to evaluate 47 patients, and if 14 or fewer responses are observed the treatment regimen will be rejected, whereas 15 or more would warrant further study of the treatment, potentially in a phase III trial. To allow for a 10% non-evaluable rate, we will enroll up to N=53 patients.

Interim safety analysis will occur after 20 patients have gone to surgery. An interim report will be generated for review by the DSMB to ensure that there are no logistical or safety issues to warrant stopping the protocol, e.g. an unacceptable rate of non-compliance with study protocol or adverse events.

In addition to the above hypothesis testing, the pathological complete response rate will be estimated and 90% confidence interval given.

9.6 Handling the missing values; censoring and discontinuations

In the primary CSR the continuing events (e.g. adverse events, concomitant therapies, etc.) will be summarized and recorded using the cut-off date as the date of completion with an indication within listings that the event is continuing.

For the patient who discontinued the study with ongoing events, the discontinuation date will be used as the completion date of the event with the appropriate censoring as described below.

The reason for discontinuation from the study will be summarized and listed, along with dates of first and last study treatment, duration of exposure to study treatment and the date of discontinuation for each patient.

Other missing data will be noted as missing on appropriate tables/listings.

Patients who have disease progression and continue receiving non-study treatment after progression will qualify for progressive disease at the time of progression and will be counted as PD in ORR and other efficacy calculations.

9.7 Secondary objectives

All secondary efficacy assessments (DOR, iDFS) will be analyzed according to the investigator's assessment as contained in the CRFs. All secondary efficacy analyses will be performed based on the FAS, unless otherwise specified.

No adjustment for multiple testing will be made.

9.8 Secondary efficacy objectives

9.8.1 Invasive disease-free survival (iDFS)

iDFS will be defined as the time from enrollment (initiation of treatment) to the first documentation of recurrence of ipsilateral invasive breast tumor, recurrence of ipsilateral locoregional invasive disease, distant recurrence, contralateral invasive breast cancer or death

from any cause. A patient who has not recurred or died at the date of the analysis will be censored at the time of the last follow up evaluation. Follow up will be by simple clinical evaluation and blood tests every 3 months with use of ultrasound (abdomen/pelvis), chest xray, CT scan or bone scan as indicated to evaluate symptoms. By default, if disease recurrence or death is documented after one single missing follow up evaluation, the actual event date of disease recurrence/death will be used for the iDFS event date. If disease recurrence or death is documented after two or more missing clinical evaluations, the iDFS time for these patients will be censored at the date of the last adequate clinical evaluation.

iDFS will be estimated using the Kaplan-Meier method (Rich et al., 2010) and appropriate summary statistics. Cox regression models will be fit to evaluate the impact of baseline variables on iDFS (Case et al., 2002).

9.8.2 Subgroup analysis

The proportions of pCR and partial responses in subgroups of patients according to demographic and tumour characteristics, namely age (< 50, ≥ 50 years), stage of disease, grade, histological type (invasive ductal, lobular, others) and ER, PrR statuses will be compared in univariate analyses and tested for statistical significance using x^2 or Fisher's exact test at alpha level of 0.05.

9.9 Safety objectives

9.9.1 Analysis set and grouping for the analysis

For all safety analyses, the safety set (SS) will be used. All listings and tables will be presented for all patients with or without full THP treatment.

The overall observation period will be divided into three mutually exclusive segments:

1. Pre-treatment period: from the day of patient's informed consent to the day before first dose of study medication
2. On-treatment period: from the day of first dose of study medication to 30 days after last dose of study medication
3. Post-treatment period: starting at the day 30+1 after last dose of Herceptin.

9.9.2 Adverse events (AEs)

Summary tables for AEs include only AEs that started or worsened during the on-treatment period, e.g. the ***treatment-emergent*** AEs. However, all safety data (including those from the pre- and post-treatment periods) will be listed, and data collected during the pre-treatment and post-treatment period are to be flagged.

The incidence of treatment-emergent AEs (new or worsening from baseline) will be summarized by system organ class and or preferred term, by the severity (based on CTCAE grades version 5), type of AE, and relation to study treatment.

Death reportable as SAE and non-fatal SAEs will be recorded for each patient and tabulated by the type of AE.

Specific safety event categories (SEC) will be considered. Such categories consist of one or more well-defined safety events that are similar in nature and for which there is a specific clinical interest in connection with the study treatment(s).

For each specified SEC, number and percentage of patients with at least one event as part of the SEC will be reported.

9.9.3 Laboratory abnormalities

For laboratory tests covered by the Common Terminology Criteria for Adverse Events (CTCAE, version 5.) the study's reporting team will grade laboratory data accordingly. For laboratory tests covered by CTCAE, a Grade 0 will be assigned for all non-missing values not graded as 1 or higher. Grade 5 will not be used.

For some tests (e.g. white blood cell differentials) the lower limits of normal ranges used in CTCAE definition have to be replaced by clinically meaningful limits expressed in absolute counts.

For laboratory tests where grades are not defined by CTCAE, results will be graded by the low/normal/high classification based on laboratory normal ranges.

The following by-treatment summaries will be generated separately for hematology, biochemistry and urinary laboratory tests:

1. Frequency table for newly occurring on-treatment grades 3 or 4 (see below for details);
2. Frequency table of low/normal/high/(or low and high) grades if for the given laboratory test the CTCAE grades were not defined;
3. Baseline concentration of Herceptin will be stated. The average and range of serum concentrations (µg/mL) pre-dose at cycles 2, 5 and 8 of Herceptin will be stated. In addition, the overall serum Herceptin exposure assessed by AUC at cycles 5 and 8 will be summarized as well as Cmax at cycles 5 and 8.

9.9.4 Other safety data

Other safety data collected will be listed and summarized using descriptive statistics as appropriate. Notable values may be flagged. Notable/abnormal values for safety data will be used for shift tables.
ECHO

1. Shift table of baseline to worst on-treatment results for overall assessments;
2. List ECHO evaluations for all patients with at least one abnormality.

Additional analyses for secondary objectives

Other secondary binary outcomes, such as clinical response rates, progressions during neoadjuvant therapy, etc. will be reported along with confidence intervals. Duration of response will be analyzed using the Kaplan-Meier method.

9.10 Patient-reported outcomes

The EORTC QLQ-C30 questionnaire along with breast module (BR23) will be used to collect patient QoL data. The raw QoL data will be scored according to the EORTC scoring manual (Fayers, 2001). The global health status/global QoL domain sub-scale score is identified as the primary QoL variable of interest. Physical functioning, social functioning, emotional functioning, nausea and vomiting, diarrhea, fatigue, and appetite loss sub-scale scores are identified as secondary QoL variables of interest. The symptom scales will be presented only for the treatment period.

All results will be presented for two patient groups. For on-treatment QoL, results will be presented for all patients, and patients switching to FEC. For follow-up QoL after treatment period, results will be presented for patients completing 4 doses of docetaxel and those completing 3 cycles of FEC, respectively.

Descriptive statistics (N, mean/proportion median, SD, Q1, Q3) will be used to summarize the individual items and scores using sub-scale scores of QoL data at each scheduled assessment time point. Patients will be included if they completed at least one questionnaire item. Additionally, deviations from screening in the domain scores at the time of each assessment will be summarized. Patients with an evaluable screening score and at least one evaluable post baseline score during the treatment period will be included in the analysis of changes from baseline using paired t-tests (assessments after disease progression will be excluded). Additionally, the baseline and follow-up values will be plotted longitudinally over time and analyzed using mixed effects regression modeling.

The compliance to the schedule of administration of validated EORTC QLQ-C30+BR23 will be summarized by patient group for each visit. Furthermore, data on the number of patients filling out the PRO/QoL data and the number of patients with the missing PRO/QoL assessments will be summarized by patient group for each visit.

The statistical analysis will comprise the estimation of the treatment effects in terms of changes in the primary and secondary QoL variables of interest during the treatment period and at the end of follow-up.

10 Biospecimens management and biomarker analysis

10.1 Biospecimens

All study subjects will provide written informed consent to allow their data and biospecimen materials to be used in proposed trial and for future translational research. The consent form will specifically state that samples will be shared with other ongoing and future studies by Nigerian researches and by the University of Chicago investigators on breast cancer in Nigerian women. The ongoing studies are: **"Identifying Genetic Alterations in Breast Cancer Samples from Nigerian Women" (GABCNW, UCMC IRB#12-2173),** with the **West African Breast Cancer Study (WABCS, collaborative study with NIBR)** and **The Nigerian Breast Cancer Study "Genetics of Breast Cancer in Blacks" (NBCS, GBCB, UCMC IRB#13304B)**. As is customary, all patients will be consented such that any sample

type taken and the resulting nucleic acid and genetic and genomic data will be available for sharing with commercial entities for research only and open to additional research studies later. The study protocol will be approved by the Institutional Review Boards/Independent Ethics Committees/Research Ethics Boards (IRB/IEC/REB) of the study sites including University Of Ibadan College of Medicine, the Lagos State University Teaching Hospital, and the Roche's legal department and the University of Chicago.

Samples acquisition, processing and shipping procedures will be performed in timely manner and in compliance with the partner Protocol "Identifying Genetic Alterations in Breast Cancer Samples from Nigerian Women" (GABCNW, UCMC IRB#12-2173) and according to NCI standards. Biospecimens from patients participating in the proposed will concurrently be eligible for the GABCNW genomic study. GABCNW protocol describes the details of sample management and biomarker analysis and is available upon request. The proposed ARETTA Protocol will share data and biospecimen material with this and another on-going case-control study "The Nigerian Breast Cancer Study" (NBCS, UCMC IRB#13304B) conducted in Ibadan, Nigeria. Subjects will therefore sign only one consent for this study to enable the samples to be shared with the above studies and future collaborative research between Nigerian investigators and UC CGH.

Biospecimen material includes FFPE tissue samples, frozen tissue and blood samples and will be collected at various time points as indicated in the trial scheme (Tables 7.1-7.3). Sampling will be performed by each investigation center and sufficient biopsies and residual tissues will be taken as part of patient's diagnostic evaluation and for further biomarker analyses. Fixed tissues will be prepared from cases with an adequate tumor volume. Each laboratory involved in the analysis of samples will be responsible for entering appropriate assay results.

10.2 Pathology consideration

As standard of care, a diagnostic pretreatment biopsy core and residual tumor tissue will be evaluated by a local pathologist according to local histopathologic standards. This includes a hematoxylin and eosin (H&E) stain and IHC assessment for ER, PgR, and HER2 according to FDA-cleared/approved protocols and ASCO/CAP recommendations. The assessment of additional markers such as Ki67 and others is not excluded. Histologic confirmation of disease will be the responsibility of each investigation site independently. Central review of IHC slides will be conducted by the Pathology Sub-commitee of the clinical trial group. A copy of the pathology reports confirming diagnosis is to be sent to the Coordinating Center HLF and Sponsor UC CGH.

10.3 Biomarkers, correlative and special studies

IHC markers based on community standards and according to the cleared/approved tests at each site's participating pathology laboratory will be collected from a participant's diagnostic breast core biopsy and residual tissues as part of the standard of care. These markers include but not restricted by the expression of tumor ER, PgR, and HER2. ER, PgR and HER2 IHC

procedures and antibodies will be used as described in UCMC Protocols IRB#12-2173 and IRB#13304B. For each marker, the original scoring parameters, e.g. intensity staining and percentage of cells stained positive will be recorded. The scoring system recommended by ASCO CAP will be applied, e.g., tumor is HRs+ if >1% nuclei stained. The cut off point described in UCMC Protocols IRB#12-2173 and IRB#13304B, e.g., tumor is HR+ if ≥ 10% cell nuclei are immunoreactive, will also be recorded. Tumor is HER2+ if weak to moderate (2+) complete membrane staining in > 10% of the tumor cells; or strong complete membrane staining (3+) observed in > 10% of the tumor cells. HER2 status will be complemented by HER2 FISH or CISH assays when appropriate. FISH/CISH testing is required for HER2 IHC 2+ cases as indicated by FDA and ASCO-CAP guidelines. See sections "Glossary of Terms and MOP. FISH/CISH assays will be performed at the UC CGH Sponsor site. Latest ASCO-CAP recommendations on HRs and HER2 statuses will also be explored as described in sections "Glossary of Terms" and MOP.

Tissue obtained during trial will be used to define molecular subtypes in core biopsy and in resected tumors to re-assess HRs/HER2 status for treatment evaluation and for assignment of adjuvant targeted therapy (tamoxifen). Tissue collected under this study will also be used by the University of Chicago investigators to generate further biomarkers and molecular data on next-generation technology platforms for ongoing **(**IRB12-2173 and 13304B) and future studies on breast cancer in Nigerian women. These molecular characteristics (biomarkers/pathways) will be used for better stratifying tumor type and to correlate with pathologic, imaging, or response to treatment measures. During the course of the trial, standard and exploratory biomarkers will be tested for their ability to predict tumor response to specific classes of investigational targeted therapeutics.

Microbiome study: To characterize the stool microbial composition, diversity, and structure of patients diagnosed with aggressive molecular subtypes of breast cancer using 16S rRNA amplicon sequencing. In addition, to explore whether there are differences in the microbiota of patients diagnosed with aggressive molecular subtypes of breast cancer based on race, diet and SCFA composition, using 16S rRNA amplicon sequencing and gas chromatography.

In the clinic, patients will be asked to provide a rectal swab the same day. Patients will then be asked if they would like to volunteer their whole stool for a more in-depth study.
Each participant will be given a sample kit in the clinic and asked to provide a sample of his/her microbiota using a non-invasive bio-collector. Sample instruction sheets will be provided and training in stool sample acquisition will be provided.

Collectors should be sealed completely and returned in the provided plastic bag. For SCFA analysis.

11 STUDY MANAGEMENT AND REGULATORY AFFAIRS

11.1 Multicenter Guidelines

The specific responsibilities of the Principal Investigator and the Coordinating Center are presented in Appendix A. Clinical studies coordinated by The University of Chicago must be

conducted in accordance with the ethical principles that are consistent with Good Clinical Practices (GCP) and in compliance with other applicable regulatory requirements

The Study Lead PI/Coordinating Center is responsible for distributing all official protocols, amendments, and IND Action Letters or Safety Reports to all participating institutions for submission to their individual IRBs for action as required.

11.2 Institutional Review Board (IRB) Approval and Consent

Unless otherwise specified, each participating institution must obtain its own IRB approval. It is expected that the IRB will have the proper representation and function in accordance with federally mandated regulations. The IRB should approve the consent form and protocol.

In obtaining and documenting informed consent, the investigator should comply with the applicable regulatory requirement(s), and should adhere to Good Clinical Practice (GCP) and to ethical principles that have their origin in the Declaration of Helsinki.
Before recruitment and enrollment onto this study, the patient will be given a full explanation of the study and will be given the opportunity to review the consent form. Each consent form must include all the relevant elements currently required by the FDA Regulations and local or state regulations. Once this essential information has been provided to the patient and the investigator is assured that the patient understands the implications of participating in the study, the patient will be asked to give consent to participate in the study by signing an IRB-approved consent form.
Prior to a patient's participation in the trial, the written informed consent form should be signed and personally dated by the patient and by the person who conducted the informed consent discussion.

11.3 Required Documentation

Before the study can be initiated at any site, the following documentation must be provided to the Cancer Clinical Trials Office (CCTO) at the University of Chicago Comprehensive Cancer Center.

- A copy of the official IRB approval letter for the protocol and informed consent
- IRB membership list
- CVs and medical licensure for the principal investigator and any sub-investigators who will be involved in the study.
- Investigational drug accountability standard operating procedures
- Additionally, before the study can be initiated at any site, the required executed research contract/subcontract must be on file.

11.4 Data and Safety Monitoring

This is a phase II feasibility and capacity building study. While the Nigerian sites' PIs are well equipped to treat cancer patients, none has served as PI on a cancer treatment trial. The University of Chicago as Sponsor will perform close monitoring of each site. We plan to start initial enrollment in Ibadan followed by LASUTH and LUTH and OAUTH. We plan to start accrual slowly but we should be able to recruit 2 patients per month at each site (4- 8 eligible patients get on study) for up to 24 months to demonstrate complete accrual. Thus, the sample size of 53 patients will be obtained in approximately 24 months or less if all sites come on board immediately as this is a capacity building study. The 1st site will be Ibadan and we will get the patients through the 1st 12 weeks before other Centers open. We anticipate bringing in additional Centers every 12 weeks. Interim analysis will occur after 20 patients have gone to surgery primarily to evaluate the response rate and SAE rate. The follow-up period will be the time on treatment up until surgery. At the time of interim analysis, study conduct and data collection, patient treatment adherence, and adverse events in aggregate will be assessed.

The most worrisome cardiac event is heart failure. After the first 20 patients have been enrolled and completed all chemotherapy till surgery, the DSMB will perform a planned

review of heart failure data. If a 6% or higher incidence of heart failure NYHA Classes III to IV or definite or probable cardiac death is observed (i.e., an absolute difference of more than 3% from the expected 3% rate of heart failure on trastuzumab plus standard of care chemotherapy) the DSMB can recommend amending or stopping the trial.

Stopping of accrual will occur if it is determined that either a) infrastructure has not developed well enough to support this study, b) patient treatment adherence is insufficient to obtain a realistic estimate of efficacy, c) adverse events are unexpectedly more frequent than noted with previous clinical experience using docetaxel with or without FEC.

HLF staff will be responsible for monitoring each site according to local regulations.
Prior to subject recruitment, and unless otherwise specified, a participating site will undergo a Site Initiation Teleconference to be conducted by the designated HLF staff and/or University of Chicago research team. The site's principal investigator and his or her study staff must attend the site initiation meeting.
Monitoring will be conducted to verify the following:

- Adherence to the protocol
- Completeness and accuracy of study data and samples collected
- Compliance with regulations
- Submission of required source documents

Participating sites will also undergo a site close-out teleconference upon completion, termination or cancellation of a study to ensure fulfillment of study obligations during the conduct of the study, and to ensure that the site Investigator is aware of his/her ongoing responsibilities.
This protocol will undergo weekly review at the University of Chicago's multi-institutional data and safety monitoring teleconference as per procedures specified by the UC CCC NCI-approved Data and Safety Monitoring Plan. The conference will review:

- Enrollment rate relative to expectations, characteristics of participants
- Safety of study participants (Serious Adverse Event & Adverse Event reporting)
- Adherence to protocol (protocol deviations)
- Completeness, validity and integrity of study data
- Retention of study participants

Protocol deviations are to be documented using the Protocol Deviation Form and sent via email to HLF. HLF will be responsible for reporting to Sponsor at PhaseIICRA@medicine.bsd.uchicago.edu within 2 business days of receipt from site. Deviations that are considered major because they impact subject safety or alter the risk/benefit ratio, compromise the integrity of the study data, and/or affect subjects' willingness to participate in the study must be reported to HLF within 7 days. All major protocol deviations should also be reported to the local IRB of record according to their policies and procedures.

11.5 Data Safety Monitoring Board

A Data Safety and Monitoring Board (DSMB) - an external seven-member board to enhance the safety of study subjects and scientific integrity of the study will be constituted. The conduct and data generated from the study will be monitored as defined in 11.4. The DSMB will meet at annually during the trial at a time determined by members. The board will review the protocol for any major concerns prior to implementation. During the trial, the DSMB should review cumulative study data to evaluate safety, study conduct, and scientific validity of the trial. The committee will check to ensure the safety of the study participants and consistency of the data generated at each of the four study sites. Consideration of adverse events rates including individual events of particular concern (e.g. SUSARs) will also be done. The committee will also monitor rates of recruitment, ineligibility, noncompliance, protocol violations and dropouts, overall and by study site with completeness and timeliness of data. Midway into the study, the board will issue an interim monitoring report (IMR) of the trial making recommendations on continuation of the study as designed, continuation of the study with minor or major modifications of protocol/informed consent, early study termination or temporary suspension of enrollment and/or study intervention until some uncertainty is resolved. They can also recommend extension of the study treatment period for participants.

11.6 Auditing

Audits provide assurance that trials are conducted and study data are collected, documented and reported in compliance with the protocol. Further, quality assurance audits ensure that study data are collected, documented and reported in compliance with Good Clinical Practices (GCP) Guidelines and regulatory requirements.
The audit will review subjects enrolled in accordance with audit procedures specified in the UC CCC Data and Safety Monitoring plan.
A regulatory authority (e.g. FDA) may also wish to conduct an inspection of the study, during its conduct or even after its completion. If an inspection has been requested by a regulatory authority, the site investigator must immediately inform HLF that such a request has been made. HLF will notify the sponsor and University of Chicago staff.

11.7 Amendments to the Protocol

All modifications to the protocol, consent form, and/or questionnaires will be submitted to the University of Chicago IRB for review and approval. A list of the proposed modifications or amendments to the protocol and/or an explanation of the need of these modifications will be submitted, along with a revised protocol incorporating the modifications. Only the Study Lead PI can authorize any modifications, amendments, or termination of the protocol. Once a protocol amendment has been approved by the University of Chicago IRB, the Regulatory Manager will send the amended protocol and consent form (if applicable) t o HLF electronically. HLF will distribute the amended protocol to all sites. Upon receipt of the packet the affiliate institution is expected to do the following:

- The amendment should be submitted to the affiliate institution's IRB as soon as possible after receipt. The amendment **must** be IRB approved by the institution **within 3 months** from the date that it was received.

- **The University of Chicago version date and/or amendment number must appear on the affiliate consent form and on the affiliate IRB approval letter.** The version dates can be found on the footer of every page of the protocol and consent form. The amendment number can be found on the University of Chicago IRB amendment approval letter that is sent with the protocol/amendment mailing.

- The IRB approval for the amendment and the amended consent form (if amended consent is necessary) for the affiliate institution must be sent to HLF staff as soon as it is received.

11.8 Annual IRB Renewals, Continuing Review and Final Reports

A continuing review of the protocol will be completed by the University of Chicago IRB and the participating institutions' IRBs at least once a year for the duration of the study. The annual IRB renewal approvals for participating institutions should be forwarded promptly to HLF staff. If the institution's IRB requires a new version of the consent form with the annual renewal, the consent form should be included with the renewal letter.

11.9 Record Retention

Study documentation includes all CRFs, data correction forms or queries, source documents, Sponsor-Investigator correspondence, monitoring logs/letters, and regulatory documents (e.g., protocol and amendments, IRB correspondence and approval, signed patient consent forms). Source documents include all recordings of observations or notations of clinical activities and all reports and records necessary for the evaluation and reconstruction of the clinical research study.
Government agency regulations and directives require that all study documentation pertaining to the conduct of a clinical trial must be retained by the study investigator. In the case of a study with a drug seeking regulatory approval and marketing, these documents shall be retained for at least two years after the last approval of marketing application in an International Conference on Harmonization (ICH) region. In all other cases, study documents should be kept on file until three years after the completion and final study report of this investigational study.

11.10 Obligations of Study Site Investigators

The Study Site Principal Investigator is responsible for the conduct of the clinical trial at the site in accordance with Title 21 of the Code of Federal Regulations and/or the Declaration of Helsinki. The Study Site Principal Investigator is responsible for personally overseeing the

treatment of all study patients. He/she must assure that all study site personnel, including sub-investigators and other study staff members, adhere to the study protocol and all FDA/GCP/NCI regulations and guidelines regarding clinical trials both during and after study completion.
The Study Site Principal Investigator at each institution or site will be responsible for assuring that all the required data will be collected and entered into the CRFs. Periodically, monitoring visits or audits will be conducted and he/she must provide access to original records to permit verification of proper entry of data.

11.11 Study finance and sponsorship

UC CGH is taking the responsibility to secure funding for the clinical trial and associated translational research. Funding will be primarily from the Breast Cancer Research Foundation (BCRF) and the NIH if we are able to obtain funding for correlative studies. Roche Global will supply study drugs.

Costs charged to patient: all routine clinical care, surgery and radiotherapy. Patients who might not be able to fully cover the expenses for these aspects of their treatment may receive some assistance depending on the availability of clinical trial funds. Recommendations for such assistance will be given by the medical social worker of the study site and by the Study site PI.

The cost of the collection and shipment of research samples and biomarker/global molecular pathways analysis using emerging technologies and instrumentation for high throughout genome analysis will be funded by existing research grants from Novartis Institute for Biomedical Research (NIBR).

Participating sites/institutions are responsible for other local costs associated with specimen collection, processing, and shipment.

11.12 Steering Committee

A Steering Committee appointed by the collaborating institutions will monitor trial progress and conduct and advice on scientific credibility of trial at each investigational site/institution and as a whole. The Steering Committee will have a total of five (5) members and will be chaired by study PI, study sponsor University of Chicago CGH.

The Steering Committee will meet face-to-face or via teleconference on a schedule to be determined. The Steering Committee regularly reviews the overall trial accrual and may request information about a trial's performance to detect barriers or issues. The Steering Committee will identify additional specific communication activities to meet the needs of the proposed protocol and to provide the overall supervision of the trial. The Steering Committee ultimately carries the responsibility for deciding whether a trial needs to be stopped on grounds of safety or efficacy.

11.13 Coordinating Center and Sponsor

This study will use the infrastructure of Healthy Life for All Foundation (HLF, http://www.hlfonline.org) to coordinate and connect all study-sites together. HLF in Nigeria will provide additional support for this study by handling all logistical activities related to this trial in Nigeria.

HLF is responsible for the following activities for all sites:

a. Patient registration/check for eligibility
b. Data entry
c. Monitoring: HLF will be responsible for monitoring each site according to local regulations
d. Training: HLF will be responsible for arranging a site initiation visit (SIV) and training each site on all aspects of the protocol including data entry, SAE reporting, etc.
e. SAE/SUSAR Reporting: HLF will be responsible for reporting SAEs and SUSARs according to local regulations. Additionally, they will report SUSARs to UC via the US CRA.
f. Auditing Sites, if necessary

In addition, HLF will submit all IRB related documents to the site IRB(s) and forward to the UC CGH as requested.

The UC CGH sponsor, in collaboration with NIBR/Roche and in collaboration with HLF, will oversee this multicenter-controlled clinical trial. These Centers are helping with identifying breast cancer burden in Nigerian women, in designing, implementation, and oversight of the study protocol and procedures, including the collection, management, reporting, and interpretation of study findings. UC CGH will provide meeting and workshops organization, training and regulatory guidance. In addition, UC CGH will perform those core lab analyses that would not be available at the study sites and conduct translational part of the study.

The University of Chicago Personalized Cancer Care Consortium (PCCC) will provide infrastructure for overall monitoring and oversight of the trial.

12 References (hard copy available upon request)

Adebamowo CA, Ajayi OO. Breast cancer in Nigeria. West Afr J Med. 2000;19(3):179-91.

Aebi S, Davidson T, Gruber G, Cardoso F, and the ESMO Guidelines Working Group. Primary breast cancer: ESMO clinical practice guidelines for diagnosis, treatment and follow-up. *Ann Oncol* 2011; **22** (suppl 6)**:** 12–24.

Agboola AO, Banjo AA, Anunobi CC, Ayoade BA, Deji-Agboola AM, Musa AA, Abdel-Fatah T, Nolan CC, Rakha EA, Ellis IO, Green AR. Molecular profiling of breast cancer in

Nigerian women identifies an altered p53 pathway as a major mechanism underlying its poor prognosis compared with British counterpart. Malays J Pathol. 2014 Apr;36(1):3-17.

Anderson BO, Cazap E, El Saghir NS, Yip CH, Khaled HM, Otero IV, et al. Optimisation of breast cancer management in low-resource and middle-resource countries: executive summary of the Breast Health Global Initiative consensus, 2010. Lancet Oncol. 2011; 12(4):387-98.

Andre F, Pusztai L. Molecular classification of breast cancer: implications for selection of adjuvant chemotherapy. Nat Clin Pract Oncol. 2006; 3(11):621-32.

Anyanwu SNC. Temporal trends in breast cancer presentation in the third world. J Exp Clin Cancer Res. 2008, 27(1) 17-25

Arowolo, O. a, Njiaju, U. O., Ogundiran, T. O., Abidoye, O., Lawal, O. O., Obajimi, M., Adetiloye, A. V., et al. (2013). Neo-adjuvant Capecitabine Chemotherapy in Women with Newly Diagnosed Locally Advanced Breast Cancer in a Resource-poor Setting (Nigeria): Efficacy and Safety in a Phase II Feasibility Study. Breast J, 19(5), 470-477.

Baker, S. D., Verweij, J., Cusatis, G. a, van Schaik, R. H., Marsh, S., Orwick, S. J., … Sparreboom, a. (2009). Pharmacogenetic pathway analysis of docetaxel elimination. *Clinical Pharmacology and Therapeutics*, *85*(2), 155–163. https://doi.org/10.1038/clpt.2008.95

Baselga, J., & Swain, S. M. (2010). CLEOPATRA: a phase III evaluation of pertuzumab and trastuzumab for HER2-positive metastatic breast cancer. *Clinical Breast Cancer*, *10*(6), 489–91. http://doi.org/10.3816/CBC.2010.n.065

Baselga, J., Cortés, J., Kim, S.-B., Im, S.-A., Hegg, R., Im, Y.-H., … CLEOPATRA Study Group. (2012). Pertuzumab plus trastuzumab plus docetaxel for metastatic breast cancer. *The New England Journal of Medicine*, *366*(2), 109–19. http://doi.org/10.1056/NEJMoa1113216

Bauer KR, Brown M, Cress RD, et al. Descriptive analysis of estrogen receptor (ER)-negative, progesterone receptor (PR)-negative, and HER2-negative invasive breast cancer, the so-called triple-negative phenotype: a population-based study hrom the California Cancer Registry. Cancer 2007; 109:1721-1728

Beuselinck B., Wildiers, H., Wynendaele, W., Dirix, L., Kains, J. P., & Paridaens, R. Weekly paclitaxel versus weekly docetaxel in elderly or frail patients with metastatic breast carcinoma: A randomized phase-II study of the Belgian Society of Medical Oncology. Crit Rev Oncol Hematol 2010; 75(1):70-7

Breast Health Global Initiative (BHGI) 2007 in http://portal.bhgi.org/Pages/Default.aspx)

Burris III., Yardley, D., Jones, S., Houston, G., Broome, C., Thompson, D., Greco, F. A., et al. Phase II trial of trastuzumab followed by weekly paclitaxel/carboplatin as first-line treatment for patients with metastatic breast cancer. J Clin Oncol. 2004. 22; (9): 1621-1629

Busolo D & Woodgate R. Cancer prevention in Africa: a review of the literature. Glob Health Promot. 2015 Jun;22(2):31-9.

Buzatto, I. P. C., Ribeiro-Silva, A., Andrade, J. M., Carrara, H. H. A., Silveira, W. A., & Tiezzi, D. G. (2017). Neoadjuvant chemotherapy with trastuzumab in HER2-positive breast cancer: pathologic complete response rate, predictive and prognostic factors. *50*(2), e5674.

Buzdar AU, Suman VJ, Meric-Bernstam F, Leitch AM, Ellis MJ et al. Fluorouracil, epirubicin, and cyclophosphamide (FEC-75) followed by paclitaxel plus trastuzumab versus paclitaxel plus trastuzumab followed by FEC-75 plus trastuzumab as neoadjuvant treatment for patients with HER2-positive breast cancer (Z1041): a randomised, controlled, phase 3 trial. Lancet Oncol. 2013 (13):1317-25.

Case L, Kimmick G, Paskett E et al., Interpreting Measures of Treatment Effect in Cancer Clinical Trials. The Oncologist June 2002 vol. 7 no. 3 181-187

Carey L, et al., Race, Breast Cancer Subtypes, and Survival in the Carolina Breast Cancer Study. JAMA 2006, 295: pp. 2492-2502.

Caudle AS, Yu TK, Tucker SL, Bedrosian I, Litton JK, Gonzalez-Angulo AM, et al. Local-regional control according to surrogate markers of breast cancer subtypes and response to neoadjuvant chemotherapy in breast cancer patients undergoing breast conserving therapy. Breast Cancer Res. 2012;14(3):R83.

DeSantis CE, Fedewa SA, Goding Sauer A, Kramer JL, Smith RA, Jemal A. Breast cancer statistics, 2015: Convergence of incidence rates between black and white women. CA Cancer J Clin. 2015 Oct 29. doi: 10.3322/caac.21320.

El Saghir NS, Adebamowo CA, Anderson BO, Carlson RW, Bird PA, Corbex M, et al. Breast cancer management in low resource countries (LRCs): consensus statement from the Breast Health Global Initiative. Breast. 2011 Apr;20 Suppl 2:S3-11. doi: 10.1016/j.breast.2011.02.006.

Fayers PM. Interpreting quality of life data: population-based reference data for the EORTC QLQ-C30. Eur J Cancer. 2001 Jul;37(11):1331-4.

Ferlay J, Soerjomataram I, Dikshit R, Eser S, Mathers C, Rebelo M et al. Cancer incidence and mortality worldwide: sources, methods and major patterns in GLOBOCAN 2012. Int J Cancer. 2015 Mar 1;136(5):E359-86

Forae G, Nwachokor F, Igbe A. Histopathological profile of breast cancer in an African population. Ann Med Health Sci Res. 2014 May;4(3):369-73.

Francis PA, Regan MM, Fleming GF et al. Adjuvant ovarian suppression in premenopausal breast cancer. N Engl J Med. 2015;372(5):436-46

Gianni, L., Pienkowski, T., Im, Y.-H., Roman, L., Tseng, L.-M., Liu, M.-C., … Valagussa, P. (2012). Efficacy and safety of neoadjuvant pertuzumab and trastuzumab in women with locally advanced, inflammatory, or early HER2-positive breast cancer (NeoSphere): a randomised multicentre, open-label, phase 2 trial. *The Lancet. Oncology*, *13*(1), 25–32. http://doi.org/10.1016/S1470-2045(11)70336-9

Gligorov, J., Ataseven, B., Verrill, M., De Laurentiis, M., Jung, K. H., Azim, H. A., … Van Thuan, T. (2017). Safety and tolerability of subcutaneous trastuzumab for the adjuvant treatment of human epidermal growth factor receptor 2-positive early breast cancer: SafeHer phase III study's primary analysis of 2573 patients. *European Journal of Cancer*, *82*, 237–246. http://doi.org/10.1016/j.ejca.2017.05.010

GLOBOCAN 2012: Estimated Cancer Incidence, Mortality and Prevalence Worldwide in 2012. GLOBOCAN database version 1.1 released November 2014. http://globocan.iarc.fr/Pages/fact_sheets_cancer.aspx

Gnant M, Thomssen C, Harbeck N.St. Gallen/Vienna 2015: A Brief Summary of the Consensus Discussion. Breast Care (Basel). 2015 Apr;10(2):124-30.

Hourcade-Potelleret, F., Lemenuel-Diot, a, McIntyre, C., Brewster, M., Lum, B., & Bittner, B. Use of a population pharmacokinetic approach for the clinical development of a fixed-dose subcutaneous formulation of trastuzumab. *CPT: pharmacometrics & systems pharmacology*, 2014 *3*; e87

Huber KE, Carey LA, Wazer DE. Breast cancer molecular subtypes in patients with locally advanced disease: impact on prognosis, patterns of recurrence, and response to therapy. Semin Radiat Oncol. 2009;19(4):204-10.

Huo, D., Ikpatt, F., Khramtsov, A., Dangou, J. M., Nanda, R., Dignam, J., Zhang, B., et al. Population differences in breast cancer: Survey in indigenous african women reveals over-representation of triple-negative breast cancer. J Clin Oncol, 2009, *27*(27), 4515-4521

Ihekwaba FN. Breast cancer in Nigerian women. Br J Surg. 1992;79(8):771-5.

Ismael, G., Hegg, R., Muehlbauer, S., Heinzmann, D., Lum, B., Kim, S. B., Pienkowski, T., et al. Subcutaneous versus intravenous administration of (neo) adjuvant trastuzumab in patients with HER2-positive, clinical stage I-III breast cancer (HannaH study): A phase 3, open-label, multicentre, randomised trial. Lancet Oncol 2012; 13(9):869-878.

Kaufmann M, von Minckwitz G, Mamounas EP, Cameron D, Carey LA, Cristofanilli M et al. Recommendations from an international consensus conference on the current status and future of neoadjuvant systemic therapy in primary breast cancer. Ann Surg Oncol. 2012 May;19(5):1508-16. doi: 10.1245/s10434-011-2108-2.

Kim SI, Sohn J, Koo JS, Park SH, Park HS, Park BW. Molecular subtypes and tumor response to neoadjuvant chemotherapy in patients with locally advanced breast cancer. Oncol. 2010;79(5-6):324-30.

Levêque D, Gigou L, Bergerat JP. Clinical pharmacology of trastuzumab. Curr Clin Pharmacol. 2008 Jan;3(1):51-5.

Lodge M, Corbex M. Establishing an evidence-base for breast cancer control in developing countries. Breast. 2011;20 Suppl 2:S65-9.

Mauri, D., Pavlidis, N., & Ioannidis, J. P. Neoadjuvant versus adjuvant systemic treatment in breast cancer: A meta-analysis. J Natl Cancer Inst 2005, *97*(3), 188-194

Mayer IA, Abramson VG, Lehmann BD, Pietenpol JA New strategies for triple-negative breast cancer--deciphering the heterogeneity. Clin Cancer Res. 2014 Feb 15;20(4):782-90.

von Minckwitz, G., Procter, M., de Azambuja, E., Zardavas, D., Benyunes, M., Viale, G., … Baselga, J. (2017). Adjuvant Pertuzumab and Trastuzumab in Early HER2-Positive Breast Cancer. *New England Journal of Medicine*, *377*(2), 122–131. http://doi.org/10.1056/NEJMoa1703643

NCCN Clinical Practice Guidelines in Oncology for Breast Cancer v.1 2016. http://www.nccn.org/ (Accessed Feb 1, 2016)

Nwafor CC, Keshinro SO. Pattern of hormone receptors and human epidermal growth factor receptor 2 status in sub-Saharan breast cancer cases: Private practice experience. Niger J Clin Pract. 2015 Jul-Aug;18(4):553-8.

Odusanya OO. Breast cancer: knowledge, attitudes, and practices of female schoolteachers in Lagos, Nigeria. Breast J. 2001;7(3):171-5.

Olopade OI, Grushko TA, Nanda R, Huo D. Advances in breast cancer: pathways to personalized medicine. Clin Cancer Res. 2008 Dec 15;14(24):7988-99.

Pagani et al. Adjuvant exemestane with ovarian suppression in premenopausal breast cancer. N Engl J Med. 2014;371(2):107-18.

Parkin DM, Fernández LMG. Use of Statistics to Assess the Global Burden of Breast Cancer. Breast J. 2006;12:S70-S80.

Partridge AH, Rumble RB, Carey LA, Come SE, Davidson NE, Di Leo A et al. Chemotherapy and targeted therapy for women with human epidermal growth factor receptor 2-negative (or unknown) advanced breast cancer: American Society of Clinical Oncology Clinical Practice Guideline. J Clin Oncol. 2014 Oct 10;32(29):3307-29.

Perou CM, Sorlie T, Eisen MB, van de Rijn M, Jeffrey SS, Rees CA, et al. Molecular portraits of human breast tumours. Nature. 2000;406(6797):747-52.

Piccart-Gebhart MJ, Procter M, Leyland-Jones B, Goldhirsch A, Untch M, Smith I, Gianni L, Baselga J, et al.. Trastuzumab after adjuvant chemotherapy in HER2-positive breast cancer. N Engl J Med. 2005;353(16):1659-72.

Pitt Jason J, Markus Riester, Yonglan Zheng, (----------) Olufunmilayo I. Olopade, Jordi Barretina. Characterization of Nigerian Breast Cancer Reveals Prevalent Homologous Recombination Deficiency and Aggressive Molecular Features. *Nat. Commun.* 2018; 10.1038/s41467-018-06616-0

Pivot, X., Gligorov, J., Müller, V., Barrett-Lee, P., Verma, S., Knoop, A., Fallowfield, L. (2013). Preference for subcutaneous or intravenous administration of trastuzumab in patients with HER2-positive early breast cancer (PrefHer): An open-label randomised study. *The Lancet Oncology*, *14*(10), 962–970. http://doi.org/10.1016/S1470-2045(13)70383-8

Pruitt, L., Mumuni, T., Raikhel, E., Ademola, A., Ogundiran, T., Adenipekun, A., Morhason-Bello, I., et al. Social barriers to diagnosis and treatment of breast cancer in patients presenting at a teaching hospital in Ibadan, Nigeria. Global Public Health 2014, 10(3), 331-344.

RAND Probing the Barriers to Conducting Clinical Research in Sub-Saharan Africa http://www.rand.org/blog/2014/09/probing-the-barriers-to-conducting-clinical-research.html (accessed Feb 7, 2016)

Rastogi P, Anderson SJ, Bear HD, Geyer CE, Kahlenberg MS, Robidoux A, et al. Preoperative chemotherapy: updates of National Surgical Adjuvant Breast and Bowel Project Protocols B-18 and B-27. J Clin Oncol [Internet]. 2008 Feb 10;26(5):778–85. Available from: http://www.ncbi.nlm.nih.gov/pubmed/18258986

Rich J, Neely J, Paniello R et al. A practical guide to understanding Kaplan-Meier curves. Otolaryngol Head Neck Surg 143(3): 331–336

Ringel I, Horwitz SB. Studies with RP 56976 (taxotere): a semisynthetic analogue of taxol. J Natl Cancer Inst. 1991;83(4):288-91.

Roche Inc. Subcute Herceptin. Data Sheet SC 150701. Roche, 2015 http://www.medsafe.govt.nz/profs/Datasheet/h/herceptinscinj.pdf

Roche 2012 A randomized open-label study to compare the pharmacokinetics, efficacy and safety of subcutaneous (SC) Herceptin (trastuzumab) with intravenous (IV) Herceptin (trastuzumab) administered in women with HER2-positive early breast cancer. Roche Clinical Trial Result Information; Protocol number: BO22227

Schneeweiss, A., Chia, S., Hickish, T., Harvey, V., Eniu, A., Hegg, R., … Cortés, J. (2013). Pertuzumab plus trastuzumab in combination with standard neoadjuvant anthracycline-containing and anthracycline-free chemotherapy regimens in patients with HER2-positive early breast cancer: a randomized phase II cardiac safety study (TRYPHAENA). *Annals of Oncology : Official Journal of the European Society for Medical Oncology*, *24*(9), 2278-84. http://doi.org/10.1093/annonc/mdt182

Seidman AD, Hudis CA, Albanell J, Tong W, Tepler I, Currie V, Moynahan ME, Theodoulou M, Gollub M, Baselga J, Norton L. Dose-dense therapy with weekly 1-hour paclitaxel infusions in the treatment of metastatic breast cancer. J Clin Oncol. 1998;16(10):3353-61.

Sherman ME, Piemonte M, Mai PL, et al. Pathologic Findings at Risk-Reducing Salpingo-Oophorectomy: Primary Results From Gynecologic Oncology Group Trial GOG-0199. JCO. 2014;32(29): 3275-3283

Soon D, Paradies E, Lee P et al. (2008). *HER2* Status and Benefit from Adjuvant Trastuzumab in Breast Cancer. N Eng J Med 358: (13); 1409-1414

Swain SM, Baselga J, Kim SB, et al. Pertuzumab, trastuzumab, and docetaxel in HER2-positive metastatic breast cancer. *N Engl J Med* 2015; 372: 724–34.

Taylor WC, Muss HB. Adjuvant chemotherapy of breast cancer in the older patient. Oncology. 2010;24(7):608-13.

WHO. Women's Health Fact Sheet. (Internet) 2009 (cited 2013 April 5, 2013); Available from: http://www.who.int/mediacentre/factsheets/fs334/en/index.html. Nigeria. Breast J. 2001; 7(3):171-5.

APPENDICES

APPENDIX A. ECOG Performance Status

**Grade**	**ECOG***
0	Fully active, able to carry on all pre-disease performance without restriction
1	Restricted in physically strenuous activity but ambulatory and able to carry out work of a light or sedentary nature, e.g., light house work, office work
2	Ambulatory and capable of all self-care but unable to carry out any work activities. Up and about more than 50% of waking hours
3	Capable of only limited self-care, confined to bed or chair more than 50% of waking hours
4	Completely disabled. Cannot carry on any self-care. Totally confined to bed or chair
5	Dead
*As published in Am. J. Clin. Oncol.:Oken, M.M., Creech, R.H., Tormey, D.C., Horton, J., Davis, T.E., McFadden, E.T., Carbone, P.P.: Toxicity And Response Criteria Of The Eastern Cooperative Oncology Group. Am J Clin Oncol 5:649-655, 1982.	

APPENDIX B. Response Evaluation Criteria In Solid Tumors, RECIST (adapted from Version 1.1)

Baseline documentation on "target" lesion

Two measurable lesions per breast made up of primary lesion in the breasts and one axillary lymph node will be identified as target lesions and recorded and measured as baseline.

Target lesion will be selected on the basis of its size (lesion with the longest diameter) and its suitability for accurate repeated measurements (by breast ultrasonography).

The longest diameter (LD) for all target lesions will be reported as the baseline LD. The baseline LD will be used as reference to characterize the objective tumor response.

All other lesions (or sites of disease) will be identified as non-target lesions and will also be recorded at baseline. Measurements of these lesions are not required, but the presence or absence of each will be noted throughout follow-up.

Response Criteria Evaluation of target lesions

Complete Response (CR): Disappearance of all target lesions

Partial Response (PR): At least a 30% decrease in the sum of the LD of target lesions, taking as reference the baseline LD

Stable Disease (SD): Neither sufficient shrinkage to qualify for PR nor sufficient increase to qualify for PD, taking as reference the smallest LD since the treatment started

Progressive Disease (PD): At least a 20% increase in the LD of target lesions, taking as reference the smallest LD recorded since the treatment started or the appearance of one or more new lesions

Evaluation of non-target lesions

Complete Response (CR): Disappearance of all non-target lesions.

Stable Disease (SD): Persistence of one or more non-target lesion(s) or/and maintenance of tumor marker level above the normal limits

Progressive Disease (PD): Appearance of one or more new lesions and/or unequivocal progression of existing non-target lesions

Ultrasound measurements:

The measurement of breast tumour size will be in 3 -dimensions, measured in 2 planes.
In the longitudinal plane, widest Longitudinal dimension is measured (LS). In the Transverse plane, widest transverse dimension (TS) and anteroposterior dimension (AP) / height is measured.

Volumetric assessment of tumours will be done Manually by multiplying LS X TS X AP X 0.523 (CONSTANT)

Lymph nodes will be scanned in 2-planes and measured in 3-dimensions (LS X AP X TS). Volume will be calculated by multiplying LS X TS X AP X 0.523 (CONSTANT)

Lymph node measurement will be separate from the primary tumour assessment at baseline and follow-up.

APPENDIX C. Quality of Life (QoL) Assessment

ENGLISH

EORTC QLQ-C30 (version 3)

We are interested in some things about you and your health. Please answer all of the questions yourself by circling the number that best applies to you. There are no "right" or "wrong" answers. The information that you provide will remain strictly confidential.

Please fill in your initials: |_|_|_|_|
Your birthdate (Day, Month, Year): |_|_|_|_|_|_|_|_|
Today's date (Day, Month, Year): 31 |_|_|_|_|_|_|_|_|

	Not at All	A Little	Quite a Bit	Very Much
1. Do you have any trouble doing strenuous activities, like carrying a heavy shopping bag or a suitcase?	1	2	3	4
2. Do you have any trouble taking a long walk?	1	2	3	4
3. Do you have any trouble taking a short walk outside of the house?	1	2	3	4
4. Do you need to stay in bed or a chair during the day?	1	2	3	4
5. Do you need help with eating, dressing, washing yourself or using the toilet?	1	2	3	4

During the past week:	Not at All	A Little	Quite a Bit	Very Much
6. Were you limited in doing either your work or other daily activities?	1	2	3	4
7. Were you limited in pursuing your hobbies or other leisure time activities?	1	2	3	4
8. Were you short of breath?	1	2	3	4
9. Have you had pain?	1	2	3	4
10. Did you need to rest?	1	2	3	4
11. Have you had trouble sleeping?	1	2	3	4
12. Have you felt weak?	1	2	3	4
13. Have you lacked appetite?	1	2	3	4
14. Have you felt nauseated?	1	2	3	4
15. Have you vomited?	1	2	3	4
16. Have you been constipated?	1	2	3	4

Please go on to the next page

ENGLISH

EORTC QLQ - BR23

Patients sometimes report that they have the following symptoms or problems. Please indicate the extent to which you have experienced these symptoms or problems during the past week.

During the past week:	Not at All	A Little	Quite a Bit	Very Much
31. Did you have a dry mouth?	1	2	3	4
32. Did food and drink taste different than usual?	1	2	3	4
33. Were your eyes painful, irritated or watery?	1	2	3	4
34. Have you lost any hair?	1	2	3	4
35. Answer this question only if you had any hair loss: Were you upset by the loss of your hair?	1	2	3	4
36. Did you feel ill or unwell?	1	2	3	4
37. Did you have hot flushes?	1	2	3	4
38. Did you have headaches?	1	2	3	4
39. Have you felt physically less attractive as a result of your disease or treatment?	1	2	3	4
40. Have you been feeling less feminine as a result of your disease or treatment?	1	2	3	4
41. Did you find it difficult to look at yourself naked?	1	2	3	4
42. Have you been dissatisfied with your body?	1	2	3	4
43. Were you worried about your health in the future?	1	2	3	4

During the past <u>four</u> weeks:	Not at All	A Little	Quite a Bit	Very Much
44. To what extent were you interested in sex?	1	2	3	4
45. To what extent were you sexually active? (with or without intercourse)	1	2	3	4
46. Answer this question only if you have been sexually active: To what extent was sex enjoyable for you?	1	2	3	4

Please go on to the next page

YORUBA

EORTC QLQ - BR23

Awon ti o ngba itoju a ma so wipe awon ma n ni irufe awon iyonu ti a ko si isale yi leekokan. E jowo, e toka si eyikeyi ninu awon iyonu yi ti o se yin larin ose to koja lo.

Laarin ose ti o koja:	Rara O	Die	Pupo die	Pupo gan-an
31 Nje enu yin ngbe?	1	2	3	4
32 Nje ounje ati ohun mimu yato lenu yin si ti tele?	1	2	3	4
33 Nje oju n dun yin, o n gun yin tabi o n wami?	1	2	3	4
34 Nje irun yin ti n ja?	1	2	3	4
35 E dahun ibeere ti o kan yi ti irun yin ba ti ja ri: Nje irun yin ti oja mu iporuru okan tabi ibanuje ba yin?	1	2	3	4
36 Nje o n reyin tabi ara yin ko da?	1	2	3	4
37 Nje ara yin ma n sadede gbona lati inu wa?	1	2	3	4
38 Nje eni efo ri?	1	2	3	4
39 Nje e ro pe ewa yin dinku nitori aisan ti o n se yin tabi itoju ti e ngba?	1	2	3	4
40 Nje e lero wipe wi wa bi obinrin yin ti dinku nipase aisan yin tabi itoju ti e ngba?	1	2	3	4
41 Nje oju n ti yin tabi o je inira fun yin lati wo ara yin nigbati e ba wa ni ihoho?	1	2	3	4
42 Nje ara yin ko teyin lorun mo?	1	2	3	4
43 Nje e n ronu nipa ilera yin lojo iwaju?	1	2	3	4

Laarin ose merin ti o koja seyin:	Rara O	Die	Pupo die	Pupo gan-an
44 Ba wo ni ibalopo ti se n wun yin to?	1	2	3	4
45 Bawo ni ara yin se ya gaga si nipa ibalopo? (pelu tabi lai si ibalopo)	1	2	3	4
46 E dahun ibeere yi ti e ba ni ibalopo: Bawo ni e se gbadun ibalopo si?	1	2	3	4

E lo si oju iwe keji

YORUBA

EORTC QLQ - BR23

Awon ti o ngba itoju a ma so wipe awon ma n ni irufe awon iyonu ti a ko si isale yi leekokan. E jowo, e toka si eyikeyi ninu awon iyonu yi ti o se yin larin ose to koja lo.

Laarin ose ti o koja:	Rara O	Die	Pupo die	Pupo gan-an
31 Nje enu yin ngbe?	1	2	3	4
32 Nje ounje ati ohun mimu yato lenu yin si ti tele?	1	2	3	4
33 Nje oju n dun yin, o n gun yin tabi o n wami?	1	2	3	4
34 Nje irun yin ti n ja?	1	2	3	4
35 E dahun ibeere ti o kan yi ti irun yin ba ti ja ri: Nje irun yin ti oja mu iporuru okan tabi ibanuje ba yin?	1	2	3	4
36 Nje o n reyin tabi ara yin ko da?	1	2	3	4
37 Nje ara yin ma n sadede gbona lati inu wa?	1	2	3	4
38 Nje eni efo ri?	1	2	3	4
39 Nje e ro pe ewa yin dinku nitori aisan ti o n se yin tabi itoju ti e ngba?	1	2	3	4
40 Nje e lero wipe wi wa bi obinrin yin ti dinku nipase aisan yin tabi itoju ti e ngba?	1	2	3	4
41 Nje oju n ti yin tabi o je inira fun yin lati wo ara yin nigbati e ba wa ni ihoho?	1	2	3	4
42 Nje ara yin ko teyin lorun mo?	1	2	3	4
43 Nje e n ronu nipa ilera yin lojo iwaju?	1	2	3	4

Laarin ose merin ti o koja seyin:	Rara O	Die	Pupo die	Pupo gan-an
44 Ba wo ni ibalopo ti se n wun yin to?	1	2	3	4
45 Bawo ni ara yin se ya gaga si nipa ibalopo? (pelu tabi lai si ibalopo)	1	2	3	4
46 E dahun ibeere yi ti e ba ni ibalopo: Bawo ni e se gbadun ibalopo si?	1	2	3	4

E lo si oju iwe keji

YORUBA

EORTC QLQ - BR23

Awon ti o ngba itoju a ma so wipe awon ma n ni irufe awon iyonu ti a ko si isale yi leekokan. E jowo, e toka si eyikeyi ninu awon iyonu yi ti o se yin larin ose to koja lo.

Laarin ose ti o koja:	Rara O	Die	Pupo die	Pupo gan-an
31 Nje enu yin ngbe?	1	2	3	4
32 Nje ounje ati ohun mimu yato lenu yin si ti tele?	1	2	3	4
33 Nje oju n dun yin, o n gun yin tabi o n wami?	1	2	3	4
34 Nje irun yin ti n ja?	1	2	3	4
35 E dahun ibeere ti o kan yi ti irun yin ba ti ja ri: Nje irun yin ti oja mu iporuru okan tabi ibanuje ba yin?	1	2	3	4
36 Nje o n reyin tabi ara yin ko da?	1	2	3	4
37 Nje ara yin ma n sadede gbona lati inu wa?	1	2	3	4
38 Nje eni efo ri?	1	2	3	4
39 Nje e ro pe ewa yin dinku nitori aisan ti o n se yin tabi itoju ti e ngba?	1	2	3	4
40 Nje e lero wipe wi wa bi obinrin yin ti dinku nipase aisan yin tabi itoju ti e ngba?	1	2	3	4
41 Nje oju n ti yin tabi o je inira fun yin lati wo ara yin nigbati e ba wa ni ihoho?	1	2	3	4
42 Nje ara yin ko teyin lorun mo?	1	2	3	4
43 Nje e n ronu nipa ilera yin lojo iwaju?	1	2	3	4

Laarin ose merin ti o koja seyin:	Rara O	Die	Pupo die	Pupo gan-an
44 Ba wo ni ibalopo ti se n wun yin to?	1	2	3	4
45 Bawo ni ara yin se ya gaga si nipa ibalopo? (pelu tabi lai si ibalopo)	1	2	3	4
46 E dahun ibeere yi ti e ba ni ibalopo: Bawo ni e se gbadun ibalopo si?	1	2	3	4

E lo si oju iwe keji

APPENDIX D: Dietary and Lifestyle Questionnaire

12. APPENDICES

APPENDIX A

GENERAL QUESTIONNAIRE

A pilot study to characterize the microbiome of triple-negative breast cancer patients

You may decline to answer any question.

Personal and Contact Information

Name: Today's Date:

Gender: Birth Date: (MM/YY)

Height: Weight:

Country of birth: Current ZIP code:

1.) How would you classify your diet?
a. Omnivore (you eat everything)
b. Omnivore but do not eat red meat
c. Vegetarian
d. Vegetarian but eat seafood
e. Vegan
2.) Are you taking a daily multivitamin?
a. Yes
b. No
3.) How frequently do you take a probiotic?
a. Never
b. Rarely (a few times/month)
c. Occasionally (1-2 times/week)
d. Regularly (3-5 times/week)
e. Daily
4.) How frequently do you take Vitamin B complex, folate or folic acid?
a. Never
b. Rarely (a few times/month)
c. Occasionally (1-2 times/week)
d. Regularly (3-5 times/week)
e. Daily
5.) How frequently do you take a Vitamin D supplement?
a. Never
b. Rarely (a few times/month)
c. Occasionally (1-2 times/week)
d. Regularly (3-5 times/week)

e. Daily

6.) Are you taking any other nutritional/herbal supplements?

a. Yes** Please indicate what you are taking in the space below

b. No

7.) Do you eat a paleo, modified paleo, primal, FODMAP, Weston-Price, or other low-grain, low processed food diet?

a. Yes

b. No

8.) Do you eat meat/dairy products from animals treated with antibiotics?

a. Yes

b. No

c. Not Sure

9.) Do you follow any other special diet restrictions other than those indicated above?

a. Yes** Please explain in the space below

b. No

10.) What is your drinking water source at home (circle all that apply)?

a. City/Tap

b. Well

c. Bottled

d. Filtered

e. Not sure

General Information

1.) What is your race/ethnicity?

a. Caucasian/White

b. Asian or Pacific Islander

c. African American/Black

d. Hispanic

e. Other ____

2.) When did you move to your current state of residence?

a. Within the past month

b. Within the past 3 months

c. Within the past 6 months

d. Within the past year

e. I have lived in my current state of residence for more than a year.

3.) I have traveled outside of the United States in the past _________. (**If you answered a, b, or c, please list the countries you have been to in the space below)

a. Month**

b. 3 months**

c. 6 months**

d. 1 year

e. I have not been outside of the United States in the past year.

4.) How many non-family roommates do you have?
a. None
b. One
c. Two
d. Three
e. More than three
5.) Do you have a dog(s)?
a. Yes**
b. No
6.) Do you have a cat(s)?
a. Yes**
b. No
7.) Do you have any other pet(s)?
a. Yes** Please list them in the space below
b. No

8.) Which is your dominant hand?
a. I am right handed
b. I am left handed
c. I am ambidextrous
9.) What is your highest level of education?
a. Did not complete high school
b. High School or GED equivalent
c. Some college or technical school
d. Associate’s degree
e. Bachelor’s degree
f. Some graduate school or professional
g. Graduate or Professional degree

General Lifestyle and Hygiene Information

1.) How often do you exercise?
a. Daily
b. Regularly (3-5 times/week)
c. Occasionally (1-2 times/week)
d. Rarely (few times/month)
e. Never
2.) Do you generally exercise indoors or outdoors?
a. Indoors
b. Outdoors
c. Both
d. Depends on the season

e. None of the above

3.) Do you bite your fingernails?

a. Yes
b. No

4.) How often do you use a swimming pool/hot tub?

a. Daily
b. Regularly (3-5 times/week)
c. Occasionally (1-2 times/week)
d. Rarely (few times/month)
e. Never

5.) How often do you smoke cigarettes?

a. Daily
b. Regularly (3-5 times/week)
c. Occasionally (1-2 times/week)
d. Rarely (few times/month)
e. Never

6.) How often do you drink alcohol?

a. Daily
b. Regularly (3-5 times/week)
c. Occasionally (1-2 times/week)
d. Rarely (few times/month)
e. Never

7.) How many drinks at a time do you consume when you do drink alcohol?

a. Not applicable
b. 1-2 servings
c. 3-6
d. More than 6

8.) What type(s) of alcohol do you typically consume (select all that apply)?

a. Beer/Cider
b. Sour beers
c. White wine
d. Red wine
e. Spirits/hard alcohol

9.) How often do you brush your teeth?

a. 2 + times/day
b. 1-2 times/day
c. Once a day
d. Never

10.) How often do you floss your teeth?

a. Daily
b. Regularly (3-5 times/week)
c. Occasionally (1-2 times/week)
d. Rarely (few times/month)
e. Never

11.) How often do you wear facial cosmetics?

a. Daily
b. Regularly (3-5 times/week)
c. Occasionally (1-2 times/week)
d. Rarely (few times/month)

12.) Do you use deodorant or antiperspirant (antiperspirants generally contain aluminum)?

a. I use deodorant
b. I use an antiperspirant
c. Not sure, but I use some form of deodorant/antiperspirant
d. I do not use deodorant or an antiperspirant

13.) Approximately how many hours of sleep to you get in an average night?
a. Less than 5 hours
b. 5-6 hours
c. 6-7 hours
d. 7-8 hours
e. 8 or more hours

14.) Do you use fabric softener when drying your clothes?
a. Yes
b. No

General Health Information

15.) How many times do you have a bowel movement in an average day?
a. Less than one
b. One
c. Two
d. Three
e. Four
f. Five or more

16.) Are you currently taking a laxative regularly?
a. Yes
b. No

17.) If you are not currently taking a laxative regularly, have you taken one in the past 6 months?
a. Yes
b. No

18.) Describe the quality of your bowel movements:
a. I tend to be constipated (have difficulty passing stool) – Type 1 and 2;
b. I tend to have diarrhea (watery stool) – Type 5, 6 and 7;
c. I tend to have normal formed stool – Type 3 and 4.
Use the chart below as a reference:

19.) I have taken antibiotics in the last ____________. (If you answered a or b, please indicate which antibiotic you took and what you were treating in the space below)
a. Week**
b. Month**
c. 6 months
d. Year
e. I have not taken antibiotics in the past year.

20.) I have received a flu vaccine in the last ____________.
a. Week
b. Month
c. 6 months

d. Year
e. I have not gotten the flu vaccine in the past year.
21.) My weight has _________ within the last 6 months.
a. Increased more than 10 pounds
b. Decreased more than 10 pounds
c. Remained stable
22.) Have you had your tonsils removed?
a. Yes
b. No
c. Not sure
23.) Have you had your appendix removed?
a. Yes
b. No
c. Not sure
24.) Have you had chickenpox?
a. Yes
b. No
c. Not sure
25.) Do you currently take prescription medication for facial acne?
a. Yes
b. No

26.) Do you use over the counter products to control facial acne?
a. Yes
b. No
27.) Do you currently take over the counter or prescription medications?
a. Yes** Please list the medications that you take daily in the space below
b. No

28.) Were you born via caesarean section (C-section)?
a. Yes
b. No
c. Not sure
29.) How were you fed as an infant?
a. Primarily breast milk
b. Primarily infant formula
c. A mixture of breast milk and formula
d. Not sure
30.) Have you been diagnosed with ADD/ADHD?
a. I do not have this condition
b. Diagnosed by a medical professional (doctor, physician assistant)
c. Diagnosed by an alternative medicine practitioner
d. Self-diagnosed
31.) Have you been diagnosed with Alzheimer's Disease/Dementia

a. I do not have this condition
b. Diagnosed by a medical professional (doctor, physician assistant)
c. Diagnosed by an alternative medicine practitioner
d. Self-diagnosed

32.) Have you been diagnosed with Asthma, Cystic fibrosis, or COPD (chronic obstructive pulmonary disease)?
a. I do not have this condition
b. Diagnosed by a medical professional (doctor, physician assistant)
c. Diagnosed by an alternative medicine practitioner
d. Self-diagnosed

33.) Have you been diagnosed with Autism or Autism Spectrum Disorder?
a. I do not have this condition
b. Diagnosed by a medical professional (doctor, physician assistant)
c. Diagnosed by an alternative medicine practitioner
d. Self-diagnosed

34.) Have you been diagnosed with autoimmune disease such as Lupus (systemic lupus erythematosus), R.A. (rheumatoid arthritis), MS (multiple sclerosis), Hashimoto's thyroiditis
a. I do not have this condition
b. Diagnosed by a medical professional (doctor, physician assistant)
c. Diagnosed by an alternative medicine practitioner
d. Self-diagnosed

35.) Have you ever been diagnosed with Candida or fungal overgrowth in the gut?
a. I do not have this condition
b. Diagnosed by a medical professional (doctor, physician assistant)
c. Diagnosed by an alternative medicine practitioner
d. Self-diagnosed

36.) Have you ever been diagnosed with Clostridium difficile (C. diff) infection?
a. I do not have this condition
b. Diagnosed by a medical professional (doctor, physician assistant)
c. Diagnosed by an alternative medicine practitioner
d. Self-diagnosed

37.) Have you ever been diagnosed with coronary artery disease, heart disease, or suffered a heart attack and/or stroke?
a. I do not have this condition
b. Diagnosed by a medical professional (doctor, physician assistant)
c. Diagnosed by an alternative medicine practitioner
d. Self-diagnosed

38.) Have you ever been diagnosed mental health illness?
a. No
b. Yes

39.) If you responded yes to question 38.) please select which disorder/s from the following list:
a. Depression
b. Bipolar disorder
c. PTSD (post-traumatic stress disorder)
d. Schizophrenia
e. Anorexia nervosa
f. Bulimia nervosa
g. Substance abuse

40.) Have you ever been diagnosed with diabetes?
a. I do not have this condition

b. Diagnosed by a medical professional (doctor, physician assistant)
c. Diagnosed by an alternative medicine practitioner
d. Self-diagnosed

41.) If you responded yes to question 38), which type of diabetes?
a. Prediabetes
b. Type I diabetes
c. Type II diabetes
d. Gestational diabetes

42.) Have you ever been diagnosed with epilepsy or seizure disorder?
a. I do not have this condition
b. Diagnosed by a medical professional (doctor, physician assistant)
c. Diagnosed by an alternative medicine practitioner
d. Self-diagnosed

43.) Have you ever been diagnosed with irritable bowel syndrome (IBS)?
a. I do not have this condition
b. Diagnosed by a medical professional (doctor, physician assistant)
c. Diagnosed by an alternative medicine practitioner
d. Self-diagnosed

44.) Have you ever been diagnosed with inflammatory bowel disease (IBD)?
a. I do not have this condition
b. Diagnosed by a medical professional (doctor, physician assistant)
c. Diagnosed by an alternative medicine practitioner
d. Self-diagnosed

45.) Have you ever been diagnosed with migraines?
a. I do not have this condition
b. Diagnosed by a medical professional (doctor, physician assistant)
c. Diagnosed by an alternative medicine practitioner
d. Self-diagnosed

46.) Have you ever been diagnosed with kidney disease?
a. I do not have this condition
b. Diagnosed by a medical professional (doctor, physician assistant)
c. Diagnosed by an alternative medicine practitioner
d. Self-diagnosed

47.) Have you ever been diagnosed with liver disease?
a. I do not have this condition
b. Diagnosed by a medical professional (doctor, physician assistant)
c. Diagnosed by an alternative medicine practitioner
d. Self-diagnosed

48.) Have you ever been diagnosed with phenylketonuria?
a. I do not have this condition
b. Diagnosed by a medical professional (doctor, physician assistant)
c. Diagnosed by an alternative medicine practitioner
d. Self-diagnosed

49.) Have you ever been diagnosed with small intestinal bacterial overgrowth (SIBO)?
a. I do not have this condition
b. Diagnosed by a medical professional (doctor, physician assistant)
c. Diagnosed by an alternative medicine practitioner
d. Self-diagnosed

50.) Have you ever been diagnosed with a skin condition?
a. I do not have this condition

b. Diagnosed by a medical professional (doctor, physician assistant)
c. Diagnosed by an alternative medicine practitioner
d. Self-diagnosed

51.) Have you ever been diagnosed with thyroid disease?
a. I do not have this condition
b. Diagnosed by a medical professional (doctor, physician assistant)
c. Diagnosed by an alternative medicine practitioner
d. Self-diagnosed

52.) Have you ever been diagnosed with acid reflux or GERD (gastro-esophageal reflux disease?
a. I do not have this condition
b. Diagnosed by a medical professional (doctor, physician assistant)
c. Diagnosed by an alternative medicine practitioner
d. Self-diagnosed

53.) Have you ever been diagnosed with thyroid disease?
a. I do not have this condition
b. Diagnosed by a medical professional (doctor, physician assistant)
c. Diagnosed by an alternative medicine practitioner
d. Self-diagnosed

54.) Have you ever been diagnosed with any other relevant medical condition not listed above? ** Please list the condition(s) in the space below

55.) Do you have seasonal allergies?
a. Yes
b. No

56.) Do you have any of the following non-food allergies? (check all that apply)
a. Drug (e.g. Penicillin)
b. Pet dander
c. Bee stings
d. Poison ivy/oak
e. Sun

57.) Are you lactose-intolerant?
a. Yes
b. No

58.) Are you gluten-intolerant?
a. I was diagnosed with celiac disease
b. I was diagnosed with gluten allergy (anti-gluten IgG), but not celiac disease
c. I do not eat gluten because it makes me feel bad
d. No

59.) I am allergic to __________ (mark all that apply)
a. Peanuts
b. Tree nuts
c. Shellfish
d. Other ** Please list your other food allergies in the space below
e. I have no food allergies that I know of.

Detailed Dietary information

1. Do you receive most (more than 75% of daily calories) of your nutrition from adult nutritional

shakes (i.e. Ensure)?
a. Yes
b. No
2. In an average week, how often do you consume meat/eggs?
a. Never
b. Rarely (less than once/week)
c. Occasionally (1-2 times/week)
d Regularly (3-5 times/week)
e. Daily
3. In an average week, how often do you cook and consume home cooked meals? (Exclude ready-to-eat meals like boxed macaroni and cheese, ramen noodles, lean cuisine)
a. Never
b. Rarely (less than once/week)
c. Occasionally (1-2 times/week)
d. Regularly (3-5 times/week)
e. Daily
4. In an average week, how often do you consume ready-to-eat meals (i.e. macaroni and cheese, ramen noodles, lean cuisine)?
a. Never
b. Rarely (less than once/week)
c. Occasionally (1-2 times/week)
d. Regularly (3-5 times/week)
e. Daily
5. In an average week, how often do you eat food prepared at a restaurant, including carry-out/take-out?
a. Never
b. Rarely (less than once/week)
c. Occasionally (1-2 times/week)
d. Regularly (3-5 times/week)
e. Daily

Grains/Fiber

6. In an average week, how often do you eat at least 2 servings of whole grains in a day?
(1 serving = 1 slice of 100% whole grain bread; 1 cup whole grain cereal like Shredded Wheat, Wheaties, Grape Nuts, high fiber cereals, or oatmeal; 3-4 whole grain crackers; ½ cup brown rice or whole wheat pasta)
a. Never
b. Rarely (less than once/week)
c. Occasionally (1-2 times/week)
d. Regularly (3-5 times/week)
e. Daily

Fruits and Vegetables

7. In an average week, how often to you consume at least 2-3 servings of fruit in a day? (1 serving = ½ cup fruit; 1 medium sized fruit; 4 oz. 100% fruit juice.)
a. Never
b. Rarely (less than once/week)
c. Occasionally (1-2 times/week)

d. Regularly (3-5 times/week)
e. Daily

8. In an average week, how often do you consume at least 2-3 servings of vegetables, including potatoes in a day? (1 serving = ½ cup vegetables/potatoes; 1 cup leafy raw vegetables)

a. Never
b. Rarely (less than once/week)
c. Occasionally (1-2 times/week)
d. Regularly (3-5 times/week)
e. Daily

9.) In an average week, how many different plants do you eat?

a. <5
b. 6-10
c. 11-20
d. 21-30
e. >30

e.g. If you consume a can of soup that contains carrots, potatoes and onion, you can count this as 3 different plants. If you consume multi-grain bread, each different grain counts as a plant.

Fermented Foods

10.) How often do you consume one or more servings of fermented vegetables in or plant products a day in an average week? (1 serving = ½ cup sauerkraut, kimchi or fermented vegetable or 1 cup of kombucha)

a. Never
b. Rarely (less than once/week)
c. Occasionally (1-2 times/week)
d. Regularly (3-5 times/week)
e. Daily

Dairy

11.) In an average week, how often do you consume at least 2 servings of milk or cheese a day? (1 serving = 1 cup milk or yogurt; 1½ - 2 ounces cheese)

a. Never
b. Rarely (less than once/week)
c. Occasionally (1-2 times/week)
d. Regularly (3-5 times/week)
e. Daily

12.) In an average week, how often do you consume milk substitutes (soy milk, lactose free milk, almond milk, etc.)?

a. Never
b. Rarely (less than once/week)
c. Occasionally (1-2 times/week)
d. Regularly (3-5 times/week)
e. Daily

13.) How often do you eat frozen desserts (ice cream/gelato/milkshakes, sherbet/sorbet, frozen yogurt, etc.)?

a. Never
b. Rarely (less than once/week)
c. Occasionally (1-2 times/week)
d. Regularly (3-5 times/week)

e. Daily

Meat/Seafood

14.) In an average week, how often do you eat red meat?
a. Never
b. Rarely (less than once/week)
c. Occasionally (1-2 times/week)
d. Regularly (3-5 times/week)
e. Daily

15.) In an average week, how often do you consume higher fat red meats like prime rib, T-bone steak, hamburger, ribs, bacon, etc.?
a. Never
b. Rarely (less than once/week)
c. Occasionally (1-2 times/week)
d. Regularly (3-5 times/week)
e. Daily

16.) How many days in an average week do you consume poultry (chicken, turkey, etc.) at least once a day?
a. Never
b. Rarely (less than once/week)
c. Occasionally (1-2 times/week)
d. Regularly (3-5 times/week)
e. Daily

17.) How many days in an average week do you consume seafood (fish, shrimp, lobster, crab, etc.)?
a. Never
b. Rarely (less than once/week)
c. Occasionally (1-2 times/week)
d. Regularly (3-5 times/week)
e. Daily

18.) How many days in an average week do you consume salted snacks (potato chips, nacho chips, corn chips, popcorn with butter, french fries etc.)?
a. Never
b. Rarely (less than once/week)
c. Occasionally (1-2 times/week)
d. Regularly (3-5 times/week)
e. Daily

19.) How many days in an average week do you consume sugary sweets (cake, cookies, pastries, donuts, muffins, chocolate etc.) at least once a day?
a. Never
b. Rarely (less than once/week)
c. Occasionally (1-2 times/week)
d. Regularly (3-5 times/week)
e. Daily

20.) How many days in an average week do you cook with olive oil (including salad dressing)?
a. Never
b. Rarely (less than once/week)
c. Occasionally (1-2 times/week)
d. Regularly (3-5 times/week)

e. Daily

21.) Consume whole eggs (does not include egg beaters or just egg whites).

a. Never

b. Rarely (less than once/week)

c. Occasionally (1-2 times/week)

d. Regularly (3-5 times/week)

e. Daily

22.) Drink 16 ounces or more of a sugar sweetened beverage such as non-diet soda or fruit drink/punch (however, not including 100 % fruit juice) in a day?
(1 can of soda = 12 ounces)

a. Never

b. Rarely (less than once/week)

c. Occasionally (1-2 times/week)

d. Regularly (3-5 times/week)

e. Daily

23.) Consume diet beverages with artificial sweeteners?

a. Never

b. Rarely (less than once/week)

c. Occasionally (1-2 times/week)

d. Regularly (3-5 times/week)

e. Daily

24.) Consume at least 1L (~32 ounces) of water in a day?

a. Never

b. Rarely (less than once/week)

c. Occasionally (1-2 times/week)

d. Regularly (3-5 times/week)

e. Daily

Supplemental Answer Section:

Please use this and any additional space necessary to describe additional data you would wish us to know to aid with interpretation.

APPENDIX E. Responsibilities of the Protocol Steering Committee Chair (Protocol Chair) and Coordinating Center

Responsibility of the Protocol Chair

1. The Protocol Chair is responsible for the coordination, development, submission, and approval of the protocol as well as its subsequent amendments. The protocol will not be rewritten or modified by anyone other than the Protocol Chair. There will be only one version of the protocol, and each participating institution will use that document. The Protocol Chair is responsible for assuring that all participating institutions are using the correct version of the protocol.
2. The Protocol Chair is responsible for the overall conduct of the study at all participating sites and for monitoring its progress. All reporting requirements will be the responsibility of the Protocol Chair.
3. The Protocol Chair will be responsible for the timely review of Adverse Events (AE) to assure safety of the patients.
4. The Protocol Chair will be responsible for the review of and timely submission of data for study analysis.

Responsibility of HLF and the Coordinating Center

1. Each participating study site will have an appropriate assurance on file with the Office for Human Research Protection (OHRP), NIH. The HLF will be responsible for assuring that each participating institution has an OHRP assurance and will maintain copies of IRB approvals from each participating site.
2. Prior to the activation of the protocol at each participating institution, an OHRP form 310 (documentation of IRB approval) will be submitted.
3. HLF is responsible for central patient registration. HLF is responsible for assuring that IRB approval has been obtained at each participating site prior to the first patient registration from that site.
4. The Coordinating Center is responsible for the preparation of all submitted data for review by the Protocol Chair.
5. The Coordinating Center will maintain documentation of AE reports. Participating institutions report AEs directly to HLF and submits AE data to the Coordinating Center via REDCap. The Coordinating Center will submit AE reports to the Protocol Chair for timely review.

Audits may be accomplished in one of two ways: (1) source documents and research records for selected patients are brought from participating sites to the HLF for audit, or (2) selected patient records may be audited on-site at participating sites. If the NCI chooses to have an audit at the Coordinating Center, then the Coordinating Center is responsible for having all source documents, research records, all IRB approval documents, NCI Drug Accountability Record forms, patient registration lists, response assessments scans, x-rays, etc. available for the audit.The UC can send representative to examine source documents on-site.

APPENDIX F. Asymptomatic decline in LVEF: Algorithm for Continuation and Discontinuation of HER2-Targeted Study Medication

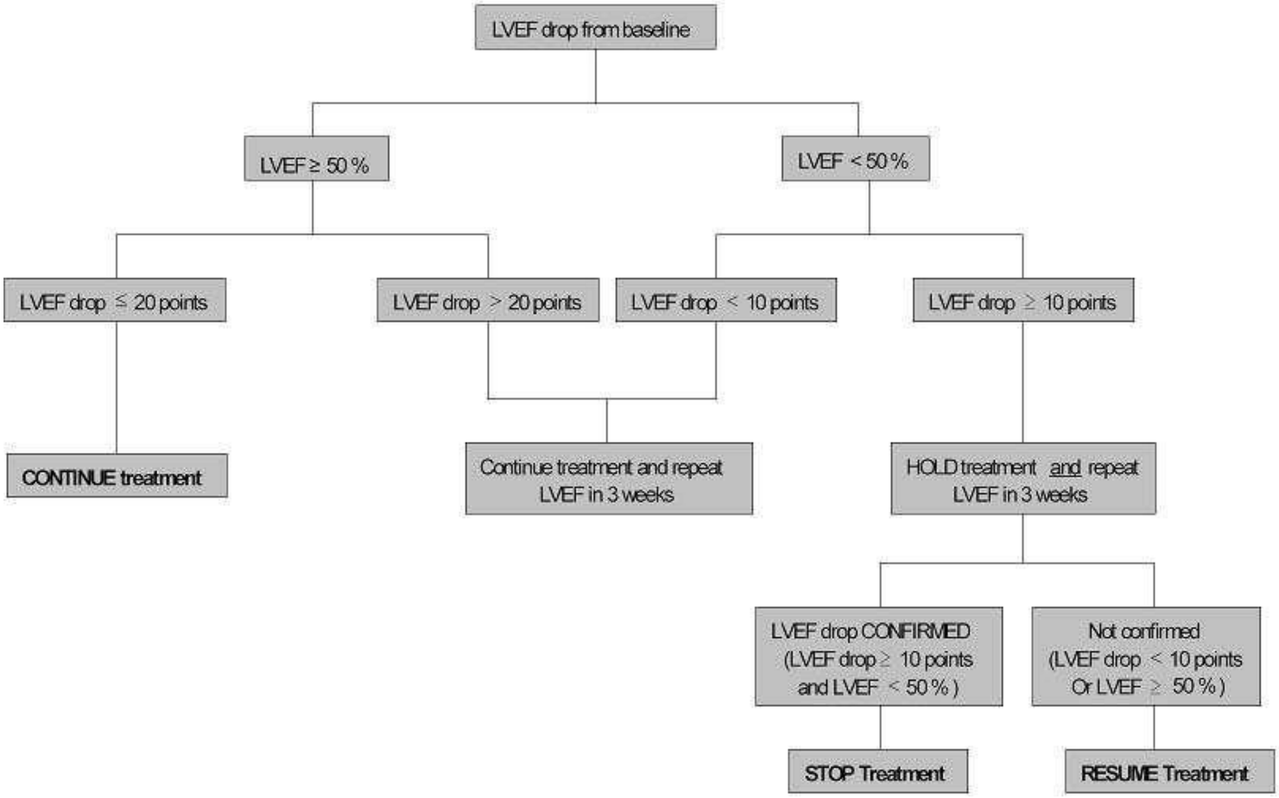

APPENDIX G. Criteria for New York Heart Association Functional Classification

Functional capacity (four classes)

Class I:	No limitation of physical activity (asymptomatic)<br><br>Ordinary physical activity does not cause undue fatigue, palpitation or dyspnea
Class II:	Slight limitation of physical activity<br><br>Comfortable at rest, but ordinary physical activity results in fatigue, palpitation or dyspnea
Class III:	Marked limitation of physical activity<br><br>Comfortable at rest, but less than ordinary activity causes fatigue, palpitation or dyspnea
Class IV:	Unable to carry on any physical activity without discomfort. Symptoms of cardiac insufficiency may be present even at rest. If any physical activity is undertaken, discomfort is increased

APPENDIX G. Nomogram for the Determination of Body Surface Area

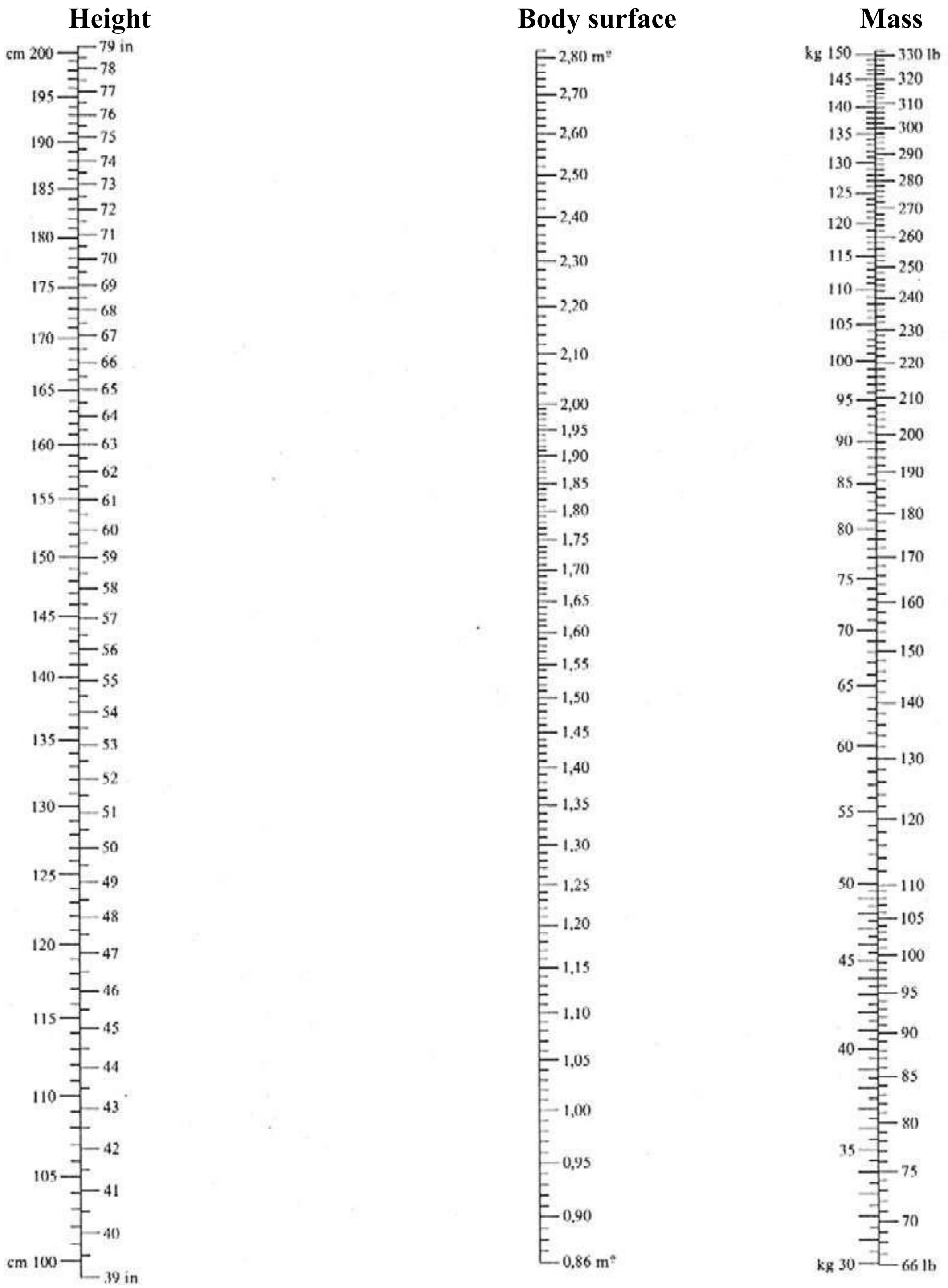

Based on the Formula from Du Bois and Du Bois, Arch intern Med, 17, 863 (1916): *0 = M0 425*x*L0 725*+x71,84 resp log*0* =log*M*x0,425+log*L*x0,725+1,8564 (0: Body surface [in cm2], M; Body mass [in kg]: L: Body length [in cm]
